# Bacterial cGAS senses a viral RNA to initiate immunity

**DOI:** 10.1038/s41586-023-06743-9

**Published:** 2023-11-15

**Authors:** Dalton V. Banh, Cameron G. Roberts, Adrian Morales-Amador, Brandon A. Berryhill, Waqas Chaudhry, Bruce R. Levin, Sean F. Brady, Luciano A. Marraffini

**Affiliations:** 1 Laboratory of Bacteriology, The Rockefeller University, New York, NY USA; 2 Weill Cornell/Rockefeller/Sloan Kettering Tri-Institutional MD-PhD Program, New York, NY USA; 3 Laboratory of Genetically Encoded Small Molecules, The Rockefeller University, New York, NY USA; 4 Department of Biology, Emory University, Atlanta, GA USA; 5 Howard Hughes Medical Institute, The Rockefeller University, New York, NY USA

**Keywords:** Bacteria, Bacteriophages

## Abstract

Cyclic oligonucleotide-based antiphage signalling systems (CBASS) protect prokaryotes from viral (phage) attack through the production of cyclic oligonucleotides, which activate effector proteins that trigger the death of the infected host^1,2^. How bacterial cyclases recognize phage infection is not known. Here we show that staphylococcal phages produce a structured RNA transcribed from the terminase subunit genes, termed CBASS-activating bacteriophage RNA (cabRNA), which binds to a positively charged surface of the CdnE03 cyclase and promotes the synthesis of the cyclic dinucleotide cGAMP to activate the CBASS immune response. Phages that escape the CBASS defence harbour mutations that lead to the generation of a longer form of the cabRNA that cannot activate CdnE03. Since mammalian oligoadenylate synthetases also bind viral double-stranded RNA during the interferon response, our results reveal a conserved mechanism for the activation of innate antiviral defence pathways.

## Abstract (web summary)

*Staphylococcus* CdnE03 cyclase recognizes structured RNA molecules produced by staphylococcal phages, triggering cyclic oligonucleotide production and, thereby, bacterial cell death—a mechanism of antiviral defence conserved across domains of life.

## Main

Many recently discovered antiviral systems in bacteria share structural and functional homology to components of metazoan innate immunity^3,4^. One example of this ancestral connection includes CBASS in bacteria^1,2^, which are analogous to the cyclic GMP-AMP synthase (cGAS)-stimulator of interferon genes (STING) antiviral pathway in metazoans^5,6^. CBASS contain two core components: a cGAS/DncV-like cyclic nucleotidyltransferase (CD-NTase, also known as Cdn) enzyme that generates cyclic nucleotides in response to viral (bacteriophage) infection^7,8^, and an effector protein that binds the cyclic nucleotides to trigger the death or growth arrest of the host and thus inhibit viral propagation^1,2,8^. In addition to the cyclase and effector genes, CBASS operons can encode accessory proteins^9–11^ that are used for their classification into four major types, I–IV^2^.

A central aspect of cyclic nucleotide-based immunity is the mechanism of activation of the cyclase—that is, how the enzyme senses viral infection to begin the synthesis of the second messenger. For human cGAS, this is achieved through direct interaction with viral double-stranded DNA present in the cytosol^6,12–15^. Other cGAS homologues present in animals, however, can sense RNA instead of DNA^16–20^. However, the mechanisms behind cyclase activation during the bacterial CBASS response are poorly understood. Biochemical analyses of bacterial cyclases from a variety of divergent CBASS operons have demonstrated that some of these enzymes are constitutively active in vitro^8^, suggesting that their activity in vivo is negatively regulated and only unleashed upon phage recognition^21^. Conversely, there are also many examples of CBASS cyclases that are inactive in vitro, and must therefore require a mechanism of activation to initiate the immune response^11^. Here we investigated the mechanism of activation of type I CBASS cyclases in staphylococci.

## CBASS neutralizes staphylococcal phages

**Fig. 1:**
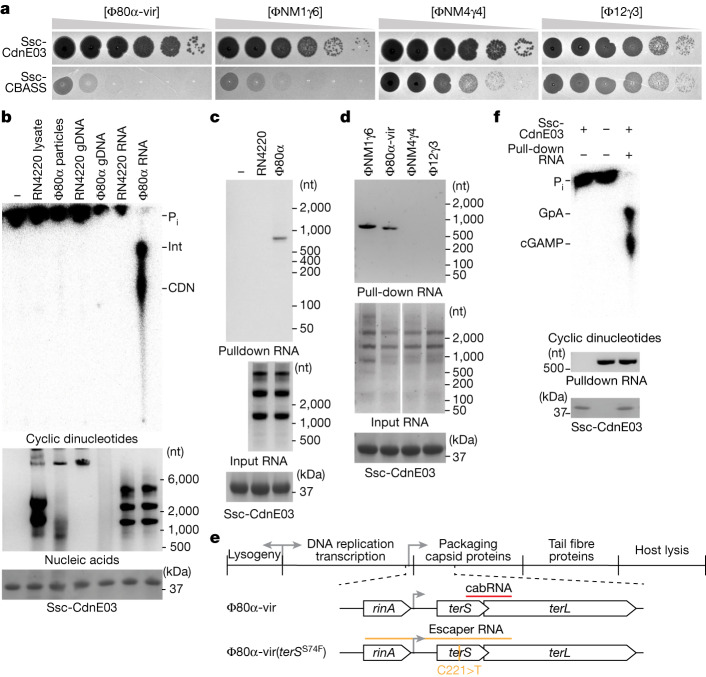
A viral RNA produced during Φ80α-vir infection activates Ssc-CdnE03 in vitro. **a**, Detection of phage propagation after spotting tenfold dilutions of the lytic DNA phages Φ80α-vir, ΦNM1γ6, ΦNM4γ4 and Φ12γ3 onto lawns of *S. aureus* RN4220 harbouring a plasmid expressing an incomplete (Ssc-CdnE03 alone) or intact Ssc-CBASS operon. **b**, Thin-layer chromatography analysis of Ssc-CdnE03 products in the presence of *S. aureus* RN4220 crude lysate, whole purified Φ80α-vir particles, host genomic DNA (RN4220 gDNA), phage genomic DNA, and total RNA from *S. aureus* RN4220 in the presence or absence of Φ80α-vir infection (before the completion of one lytic cycle). An agarose gel stained with ethidium bromide (middle) and SDS–PAGE stained with Coomassie blue (bottom) are shown as loading controls. P_i_, free phosphates; int, intermediate cyclase product; CDN, cyclic dinucleotide. Data are representative of three independent experiments. In all main figures, size in nucleotides (nt) is with reference to a single-strand RNA (ssRNA) ladder. **c**, Agarose gel electrophoresis of the input and output RNA obtained after incubation of Ssc-CdnE03 with no RNA, total RNA extracted from uninfected staphylococci (RN4220) or from cells infected with Φ80α-vir phage. SDS–PAGE stained with Coomassie blue (bottom) is shown as a loading control. Data are representative of three independent experiments. **d**,As in **c**, but with input RNA extracted from staphylococci infected with ΦNM1γ6, Φ80α-vir, ΦNM4γ4 or Φ12γ3 phages. Data are representative of three independent experiments. **e**, Diagram of Φ80α-vir and Φ80α-vir(*terS*^S74F^) genomes with localization of the cabRNA and escaper RNA sequences, respectively. The location of the escaper mutation, C221>T, is shown. **f**, As in **b**, but using cabRNA isolated from a pull-down assay. Data are representative of three independent experiments.

**Extended Data Fig. 1:**
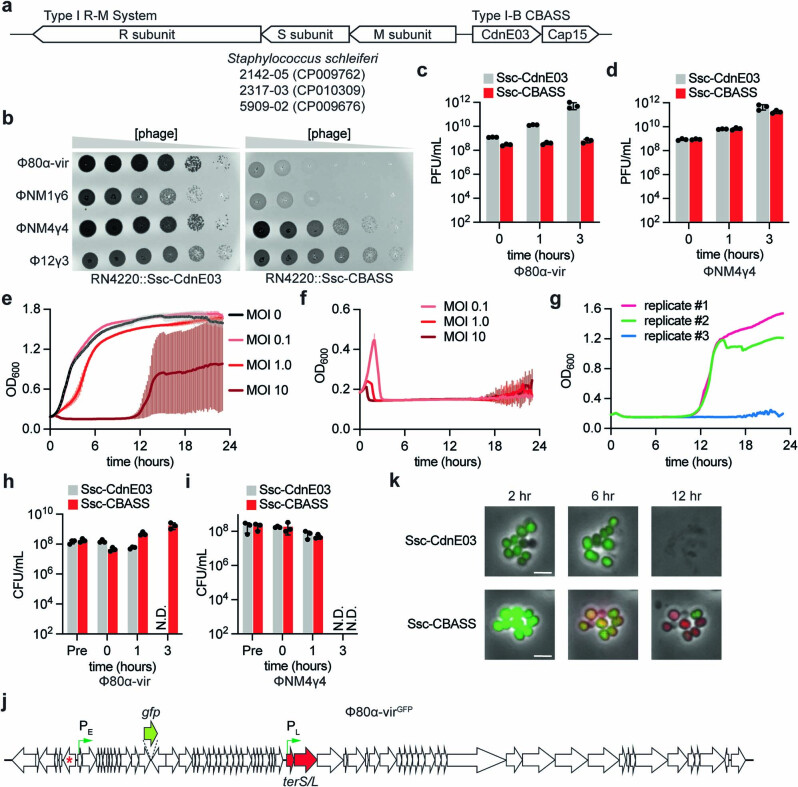
Type I-B CBASS confers anti-bacteriophage immunity in staphylococci. (**a**) Schematic of the type I-B CBASS operon present in *Staphylococcus schleiferi* (*Ssc*) 2142-05, 2317-03, and 5909-02 genomes, flanked by a type I restriction-modification system. The CBASS operon consists of two genes encoding a cyclase belonging to the E clade, cluster 3 (Ssc-CdnE03) and a two-transmembrane domain-containing effector, Cap15. (**b**) Detection of phage propagation after spotting ten-fold dilutions of the lytic DNA phages, Φ80α-vir, ΦNM1γ6, ΦNM4γ4, and Φ12γ3 onto lawns of *S. aureus* RN4220 harboring either an incomplete (Ssc-CdnE03 alone) or intact Ssc-CBASS operon integrated in its genome. (**c**) Enumeration of plaque-forming units (PFU) from cultures harboring Ssc-CdnE03 alone or Ssc-CBASS immediately before infection (Pre), after initial absorption of the phage (0 h), after one lytic cycle (1 h), and after complete culture lysis (3 h) by Φ80α-vir at MOI 5. Mean ± SEM of three biological replicates is reported. (**d**) Same as (**c**) but using ΦNM4γ4. Mean ± SEM of three biological replicates is reported. (**e**) Growth of staphylococci harboring a Ssc-CBASS operon measured by optical density at 600 nm after the addition of Φ80α-vir at a multiplicity of infection (MOI) of 0, 0.1, 1, or 10. The mean of three biological replicates ± SD is reported. (**f**) Same as (**e**) but following the growth of staphylococci expressing only the Ssc-CdnE03 cyclase. The mean of three biological replicates ± SD is reported. (**g**) Replicates of the infection shown in panel (**e**), at MOI 10. The re-growth of two of the curves at ~11 h after infection is presumably due to the selection of non-CBASS resistant bacteria, which are present at a very low frequency in the population. (**h**) Enumeration of colony-forming units (CFU) from cultures harboring Ssc-CdnE03 alone or Ssc-CBASS immediately before infection (Pre), after initial absorption of the phage (0 h), after one lytic cycle (1 h), and after complete culture lysis (3 h) by Φ80α-vir at MOI 5. Mean ± SEM of three biological replicates is reported. (**i**) Same as (**h**) but using ΦNM4γ4. Mean ± SEM of three biological replicates is reported. (**j**) Genome of the Φ80α-vir^GFP^ phage, showing the site of insertion of the *gfp* gene, the early (P_E_) and late (P_L_) promoters activated during the lytic cycle of the phage (green arrows), the gene inactivated to prevent the lysogenic cycle of Φ80α (cI-like repressor, red star), and the terminase subunit genes (*terS/L*, in red). (**k**) Fluorescence microscopy of staphylococci harboring Ssc-CdnE03 alone or the full Ssc-CBASS operon at different times after infection with Φ80α-vir^GFP^ phage, in the presence of propidium iodide. Green cells indicate a successful infection due to the expression of the viral GFP. Red cells indicate activation of the Cap15 CBASS effector, which causes membrane disruption and enables the internalization of the dye. Scale bar corresponding to 2 μm is displayed. Data are representative of three independent experiments.

Bioinformatic analyses have previously uncovered more than 100 CBASS operons in diverse *Staphylococcus* strains^2^. We characterized a type I-B CBASS present in *Staphylococcus schleiferi* strains 2142-05, 2317-03 and 5909-02^22^, hereafter designated Ssc-CBASS (Extended Data Fig. 1a). This system consists of a two-gene operon harbouring a Cdn belonging to the E clade, cluster 3^8^ (Ssc-CdnE03) and a transmembrane effector, Cap15, which was recently demonstrated to limit phage propagation by disrupting the host membrane^23^. Since we were unable to identify a phage that infects this organism, we cloned Ssc-CBASS, as well as the cyclase gene alone as a control, into the staphylococcal vector pC194^24^ for expression in the laboratory strain *Staphylococcus aureus* RN4220^25^. The resulting strain was infected with four lytic phages on soft-agar plates to quantify plaque formation. We found that Ssc-CBASS, but not Ssc-CdnE03 alone, strongly reduced the propagation of Φ80α-vir (a lytic derivative of the temperate phage Φ80α^26^ created for this study) and ΦNM1γ6^27^, but not for ΦNM4γ4^28^ (although plaque size was reduced) or Φ12γ3^29^ (Fig. 1a and Supplementary Fig. 1, which provides all the unedited images presented in this study). Similar results were obtained using a chromosomally expressed Ssc-CBASS (Extended Data Fig. 1b). Quantification of plaque-forming units (PFU) over time corroborated these results (Extended Data Fig. 1c,d). Consistent with previous reports^1^, infection of liquid cultures with Φ80α-vir at different multiplicity of infection (MOI) showed that Ssc-CBASS, but not Ssc-CdnE03 alone, enables a complete recovery of the bacterial population at low phage concentrations (Extended Data Fig. 1e–g). These results were corroborated by counting colony-forming units (CFU) (Extended Data Fig. 1h,i). Finally, we used fluorescence microscopy to analyse the Ssc-CBASS response in more detail. We infected cells with a modified Φ80α-vir that expresses GFP (Φ80α-vir^GFP^; Extended Data Fig. 1j), in the presence of propidium iodide in the growth medium. This dye displays red fluorescence upon being incorporated into the cytoplasm of bacteria with a damaged membrane. We observed that staphylococci harbouring the Ssc-CBASS system displayed green fluorescence after infection and then red fluorescence, without lysing (Extended Data Fig. 1k). By contrast, in the absence of Cap15, cells emit green—but not red—fluorescence, and lyse. Together, these data show that, similar to other species, Ssc-CBASS protects staphylococcal populations by triggering membrane disruption without lysis to prevent the growth of infected hosts and limit viral propagation.

## A phage RNA activates Ssc-CdnE03

**Extended Data Fig. 2:**
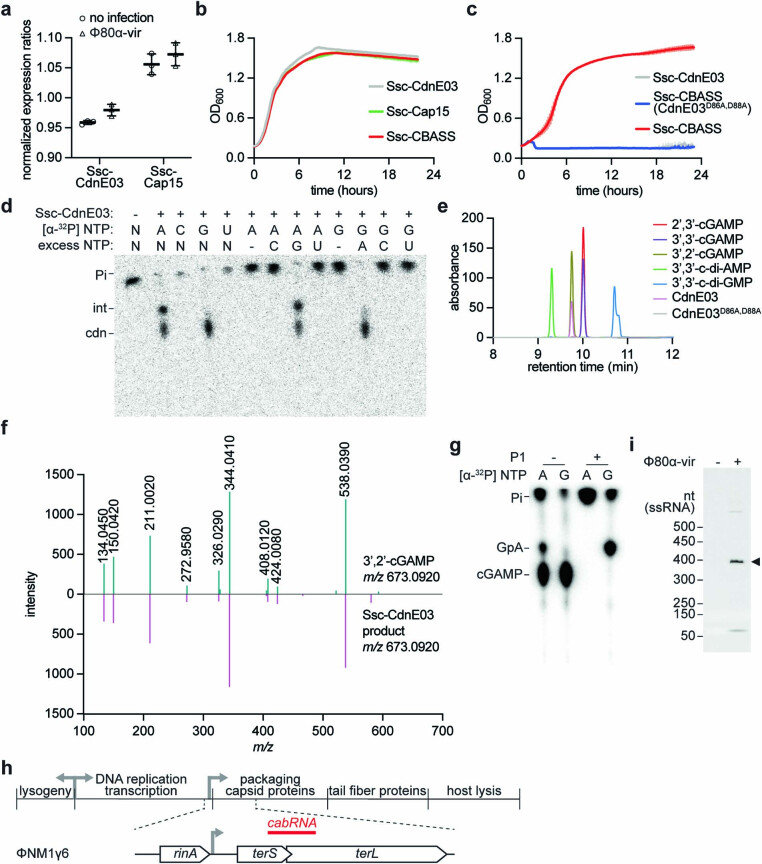
Activation of Ssc-CdnE03 in vivo and in vitro. (**a**) Regulation of Ssc-CBASS operon. Expression of the Ssc-CdnE03 and Ssc-Cap15 effector genes during log-phase growth of *S. aureus* RN4220::Ssc*-*CBASS in the presence or absence of infection by Φ80α-vir measured by RT-qPCR. For each condition, expression ratios were determined by normalizing Cq values for Ssc-CBASS genes to Cq values for the housekeeping gene *glcC*. The mean of three biological replicates ± SEM is reported. No significant differences were observed between values obtained in the presence or absence of phage, suggesting that there is not transcriptional regulation of the Ssc-CBASS operon upon infection. (**b**) Growth of staphylococci harboring an overexpression plasmid containing either the Ssc-CdnE03 alone, Ssc-Cap15 alone, or the intact Ssc-CBASS operon under the transcriptional control of a P-*spac* promoter, measured by optical density at 600 nm after the addition of IPTG. The mean of three biological replicates ± SD is reported. (**c**) Growth of staphylococci harboring either an incomplete (Ssc-CdnE03 alone) or intact Ssc-CBASS operon, with either wild-type or D86A,D88A mutant Ssc-CdnE03, measured by optical density at 600 nm after the addition of Φ80α-vir at a MOI of 1. The mean of three biological replicates ± SD is reported. (**d**) Thin-layer chromatography analysis of Ssc-CdnE03 products in the presence of total RNA from *S. aureus* RN4220 after Φ80α-vir infection, using different radiolabeled nucleotides to investigate the nucleotide composition of the enzymatic product. Pi, free phosphates; int, intermediate cyclase product; cdn, cyclic dinucleotide. Data are representative of three independent experiments. (**e**) TIC analysis of the reaction products of wild-type Ssc-CdnE03. The retention time of the peak corresponding to the cyclase products coincides with the retention time of the 3’,2’-cGAMP. This peak is not present in the reaction products of the active site mutant cyclase, D86A-D88A. Peaks for other cyclic dinucleotides that contain adenine and guanosine, which do not overlap with 3’,2’ cGAMP, are shown. (**f**) Comparison of averaged MS/MS spectra of the reaction products of wild-type Ssc-CdnE03 (purple spectrum) and 3’,2’-cGAMP (green spectrum). The most abundant ions are present in both samples (see Supplementary Text for a complete MS analysis). (**g**) Treatment of the Ssc-CdnE03 reaction products with P1 nuclease. As shown in (**d**), after treatment of the Ssc-CdnE03 reaction products with calf intestinal phosphatase (CIP), an intermediate species is observed when ATP, but not when GTP, is radiolabeled (“-”). Given that the Ssc-CdnE03 product is 3’,2’-cGAMP, this intermediate should result from the formation of either the canonical (pppG[3’−5’]pA) or the non-canonical (pppA[2’-5’]pG) phosphodiester bond during the first step of the cyclase reaction. To distinguish between these two possibilities, the reaction products were treated with CIP and P1 nuclease (“+”), which cleave 5’ and 3’ phosphodiester bonds, respectively. This treatment eliminated both the ^32^P-labeled intermediate and cyclic nucleotide when ATP was radiolabeled. In contrast, when GTP was radiolabeled, treatment with both enzymes eliminated the radioactive cyclic nucleotide to generate the linear intermediate. This result is consistent with the formation of a (pppG[3’-5’]pA) intermediate, as shown in the Supplementary Text File. Data are representative of three independent experiments. (**h**) Diagram of the ΦNM1γ6 genome showing the localization of the cabRNA sequence. (**i**) Northern blot analysis of RNA extracted from *S. aureus*/pSsc-CBASS cells before (-) and 30 min after (+) infection with Φ80α-vir, using a cabRNA probe. A 400-nt RNA that is present only during phage infection, presumably the cabRNA, is marked by the arrowhead. Data are representative of three independent experiments.

Next, we investigated how Ssc-CBASS is activated by Φ80α-vir. First, using quantitative PCR with reverse transcription (RT–qPCR) (Extended Data Fig. 2a) and overexpression (Extended Data Fig. 2b) assays, we ruled out the possibility of transcriptional activation of the operon during infection^30^. We therefore searched for CBASS activators by performing nucleotide synthesis assays in vitro using purified Ssc-CdnE03 and trace ^32^P-labelled NTPs, and visualizing the reaction products using thin-layer chromatography^8^. We tested *S. aureus* RN4220 crude lysate, purified Φ80α-vir particles, host genomic DNA, phage genomic DNA and total RNA from uninfected and infected *S. aureus* RN4220 cells. Notably, only RNA isolated from cells infected with Φ80α-vir enabled the generation of a cyclic nucleotide product by wild-type Ssc-CdnE03 (Fig. 1b) but not the active site mutant D86A/D88A that does not mediate immunity (Extended Data Fig. 2c). Further analysis of this product by performing reactions with different radiolabelled NTPs and using liquid chromatography–mass spectrometry and P1 nuclease digestion identified it as 3′,2′-cyclic guanosine monophosphate–adenosine monophosphate (3′,2′)-cGAMP, with the reaction occurring through the pppG[3′−5′]pA linear intermediate, GpA (Extended Data Fig. 2d–g and Supplementary Text).

To identify the activating RNA species, we immobilized a hexahistidyl-tagged, maltose-binding protein fusion of Ssc-CdnE03 to a cobalt resin column that was loaded with total RNA extracted from either infected or uninfected staphylococci. Extraction and separation of the nucleic acids bound by the cyclase revealed the presence of an RNA that migrated at approximately 800 nucleotides in length (compared with an ssRNA ladder; Fig. 1c). We isolated a similar species for the Ssc-CBASS-sensitive ΦNM1γ6 phage, but not for the resistant ΦNM4γ4 and Φ12γ3 viruses (Fig. 1d). Next-generation sequencing of the isolated Φ80α-vir RNA determined that it mapped to a 400-nucleotide region beginning within the *gp40* gene and extending into *gp41*, which encode the terminase small subunit (TerS) and large subunit (TerL) (Fig. 1e and Supplementary Sequences). Similar results were obtained for the cyclase-bound RNA generated during ΦNM1γ6 infection (Extended Data Fig. 2h and Supplementary Sequences). Northern blot analysis of total RNA extracted from *S. aureus* RN4220 using a probe complementary to this RNA sequence confirmed the presence of an approximately 400 nt RNA only during phage infection (Extended Data Fig. 2i). Finally, we purified the RNA molecules obtained during the pull-down assays and found that they activate cGAMP production in vitro (Fig. 1f). We therefore named this viral-derived RNA CBASS-activating bacteriophage RNA (cabRNA).

## cabRNA contains secondary structures

**Fig. 2:**
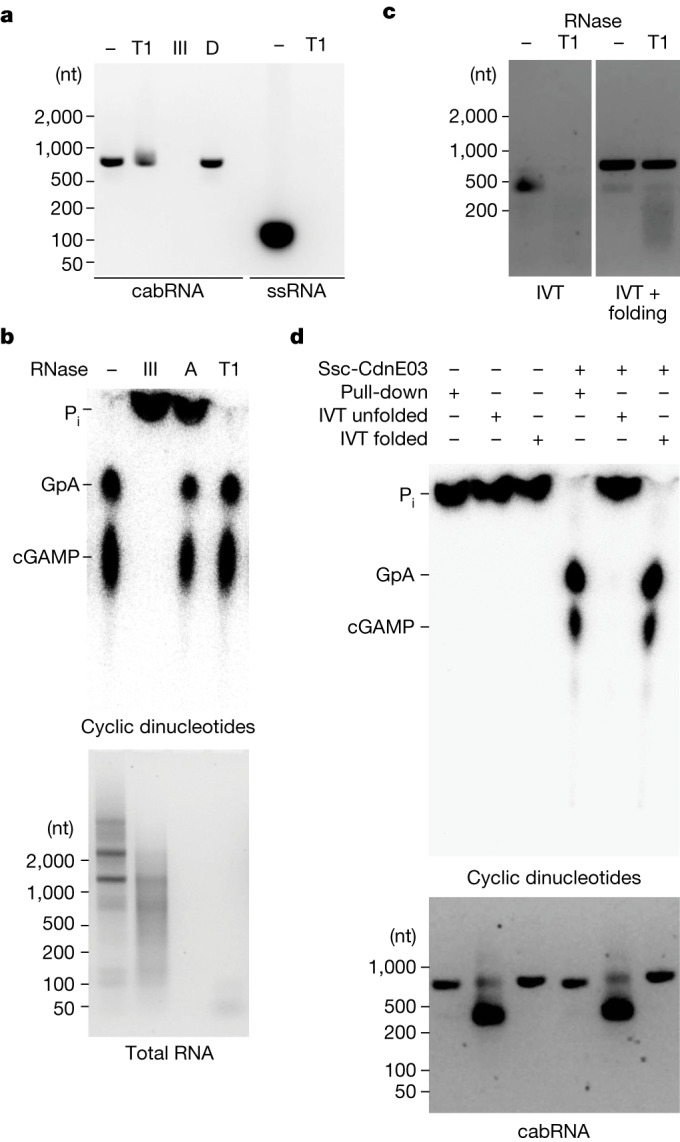
Secondary structures within the cabRNA are required for Ssc-CdnE03 activation. **a**, Agarose gel electrophoresis of pulled-down cabRNA treated with RNases T1 or III, or DNase (D). A ssRNA oligonucleotide was digested with RNase T1 as a control. Data are representative of three independent experiments. **b**, Thin-layer chromatography analysis of Ssc-CdnE03 products in the presence of total RNA extracted from infected cells and treated with RNases III, A, T1 or untreated. Data are representative of three independent experiments. **c**, Agarose gel electrophoresis of IVT cabRNA, unfolded (left) and folded (right), untreated or treated with RNase T1. Data are representative of three independent experiments. **d**, As in **b**, but incubating the cyclase with pulled-down, unfolded or folded IVT cabRNA.

The electrophoretic migration of cabRNA, higher than its nucleotide length, indicates the existence of secondary structures that may be important for cyclase activation. Using the ViennaRNA package^31^, we found several hairpins and double-stranded RNA (dsRNA) regions within the cabRNA (Supplementary Sequences). To test for the presence of these structures, we used RNase A, RNase T1 (both of which cleave ssRNA) and RNase III^32^ (which degrades dsRNA). We found that RNase III, but not RNase T1, degraded the pulled-down cabRNA (Fig. 2a). We also treated the total RNA extracted from infected staphylococci and found that RNase III, but not RNase T1 or RNase A, abrogated the ability to induce cGAMP production (Fig. 2b). These results demonstrate that dsRNA structures, but not ssRNA, within the cabRNA are important for Ssc-CdnE03 activation.

**Extended Data Fig. 3:**
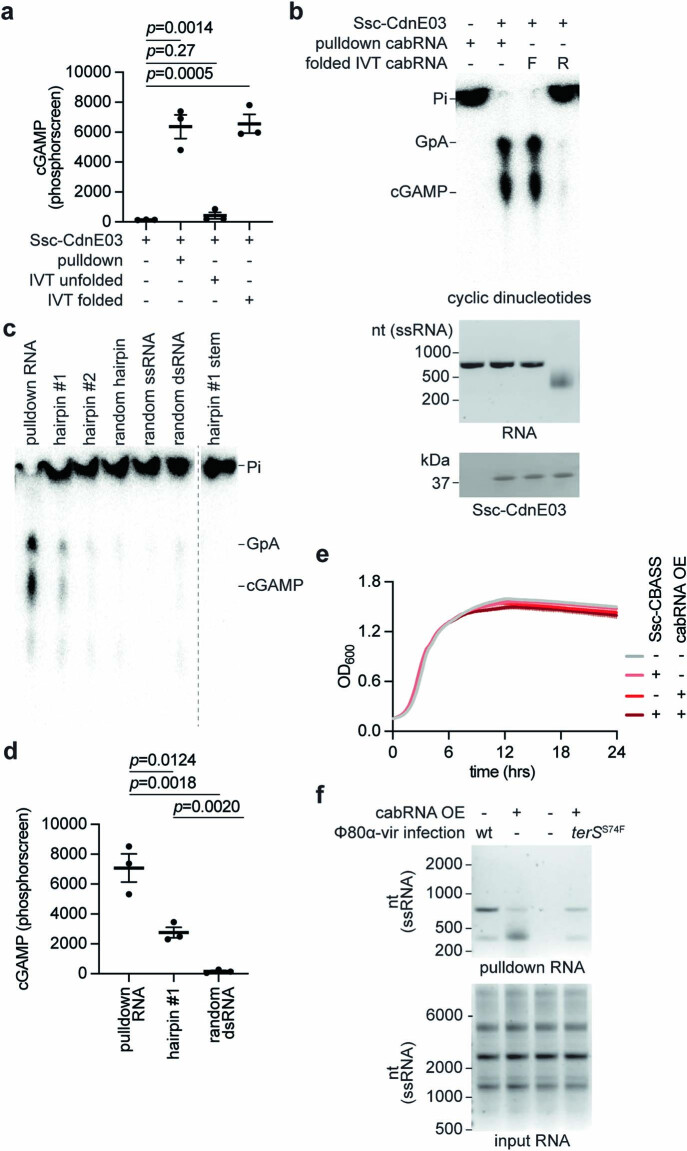
Analysis of cabRNA activation of Ssc-CdnE03. (**a**) Quantification of the cyclase reaction products of triplicates of the experiment shown in Fig. 3d. *p* values determined using a *t*-test (unpaired, two-tailed), individual data points are shown with error bars representing the mean +/− s.e.m of n = 3 technical replicates representative of three independent experiments. (**b**) Thin-layer chromatography analysis of Ssc-CdnE03 reaction products in the presence of the cabRNA isolated from a pull-down assay, or the sense or antisense strands of in vitro transcribed (IVT) cabRNA. IVT RNA was subjected to heat refolding (see Methods). An agarose gel stained with ethidium bromide (middle) and SDS-PAGE stained with Coomassie blue (bottom) are shown as loading controls. Pi, free phosphates. Data are representative of three independent experiments. (**c**) Same as in (**b**) but incubating the cyclase with pulled-down RNA or different synthetic RNA oligonucleotides described in detail in the Supplementary Sequences File and Supplementary Methods Table 5. (**d**) Quantification of the cyclase reaction products of triplicates of the experiment shown in (**c**), induced by the pull-down, hairpin−1 and random dsRNA oligonucleotide. *P* values determined using a *t*-test (unpaired, two-tailed), individual data points are shown with error bars representing the mean +/− s.e.m of n = 3 technical replicates representative of three independent experiments. (**e**) Growth of staphylococci harboring either an incomplete (Ssc-CdnE03 alone, “-”) or intact Ssc-CBASS (“+”) operon and an empty vector (“-”) or a plasmid encoding cabRNA (“+”) under the control of an aTc inducible promoter measured by optical density at 600 nm. The mean of three biological replicates ± SD is reported. (**f**) Agarose gel electrophoresis of the input and output RNA obtained after incubation of Ssc-CdnE03 with total RNA extracted from cells infected with Φ80α-vir or Φ80α-vir(*terS*^S74F^) phage, in the presence or absence of cabRNA plasmid overexpression. Data are representative of three independent experiments.

To determine whether the cabRNA alone is sufficient for activation of the cyclase, we produced cabRNA in vitro using T7 RNA polymerase. The obtained transcript migrated similar to a ssRNA of approximately 400 nucleotides after agarose gel electrophoresis and was completely digested by RNase T1; that is, it lacked secondary structures (Fig. 2c). To promote the folding of the in vitro transcribed (IVT) cabRNA, we heated the sample to 95 °C for 5 min before slowly cooling to room temperature. After this treatment, the IVT cabRNA migrated at approximately 800 nt and was resistant to RNase T1 degradation (Fig. 2c). Notably, only the folded species induced cGAMP synthesis to the same high levels as the cabRNA produced in vivo (Fig. 2d and Extended Data Fig. 3a). We also tested the activating properties of an IVT RNA with a sequence complementary to that of the cabRNA, heated and cooled to promote folding, and found it unable to activate Ssc-CdnE03 (Extended Data Fig. 3b). Finally, we used synthetic RNA oligonucleotides with the sequences of the two most prominent predicted hairpins (hairpin-1 and hairpin-2; Supplementary Sequences) and found that—albeit at lower levels than the full cabRNA—hairpin-1, but not hairpin-2, induced cGAMP production (Extended Data Fig. 3c,d). Other synthetic RNAs, including the stem portion of hairpin-1 (without the bulge and loop), an unrelated hairpin, a dsRNA and a ssRNA oligonucleotide (all with a similar size to hairpin-1 but with a different sequence; Supplementary Sequences) did not activate the cyclase (Extended Data Fig. 3c,d). Together, these data indicate that specific secondary and/or tertiary structures within the cabRNA are essential for activation of Ssc-CdnE03.

We also investigated the ability of the cabRNA to activate Ssc-CBASS in vivo in the absence of phage infection, which given that Ssc-CBASS causes cell death without lysis (Extended Data Fig. 1k), should result in a proliferation defect and/or death of staphylococci. However, cabRNA transcription using an anhydrotetracycline (ATC)-inducible promoter did not affect the growth of the cultures (Extended Data Fig. 3e). Pull-down assays using total RNA extracted from this strain after addition of ATC (Extended Data Fig. 3f) recovered a cabRNA with an electrophoretic mobility consistent with the RNase T1-sensitive, unfolded form of the cyclase inducer (lower band). This observation suggests that the unfolded cabRNA can interact with—but not activate—Ssc-CdnE03. Given that mostly RNase III-sensitive, folded cabRNA is detected during pull-down assays using total RNA extracted from infected cells (Fig. 1c,d and Extended Data Fig. 3f), we speculate that Φ80α-vir infection is critical for the proper generation, modification and/or folding of the inducer RNA.

## A positive surface of CdnE03 binds the cabRNA

**Fig. 3:**
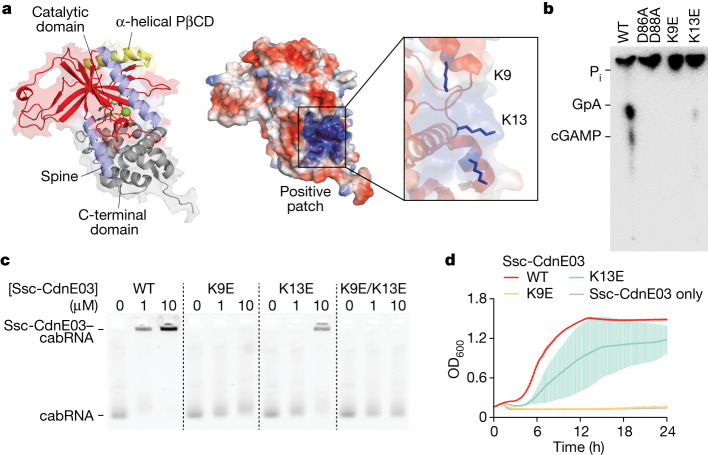
A positively charged surface within Ssc-CdnE03 binds the cabRNA to initiate immunity. **a**, AlphaFold model of Ssc-CdnE03 displayed with surface electrostatics (−77 to +77, red to blue). Inset, positively charged region harbouring the mutated lysine residues 9 and 13. **b**, Thin-layer chromatography analysis of the products of different Ssc-CdnE03 lysine mutants in the presence of total RNA extracted from infected cells. A representative image of multiple replicates is shown. WT, wild type. **c**, Electrophoretic mobility shift assay of cabRNA in the presence of increasing concentrations of different Ssc-CdnE03 mutants. **d**, Growth of staphylococci harbouring the Ssc-CBASS operon with wild-type, K9E or K13E Ssc-CdnE03 measured by optical density at 600 nm (OD_600_) after infection with Φ80α-vir at a MOI of 1. A culture expressing only Ssc-CdnE03 is used as a control. Data are mean ± s.d. of three biological replicates.

**Extended Data Fig. 4:**
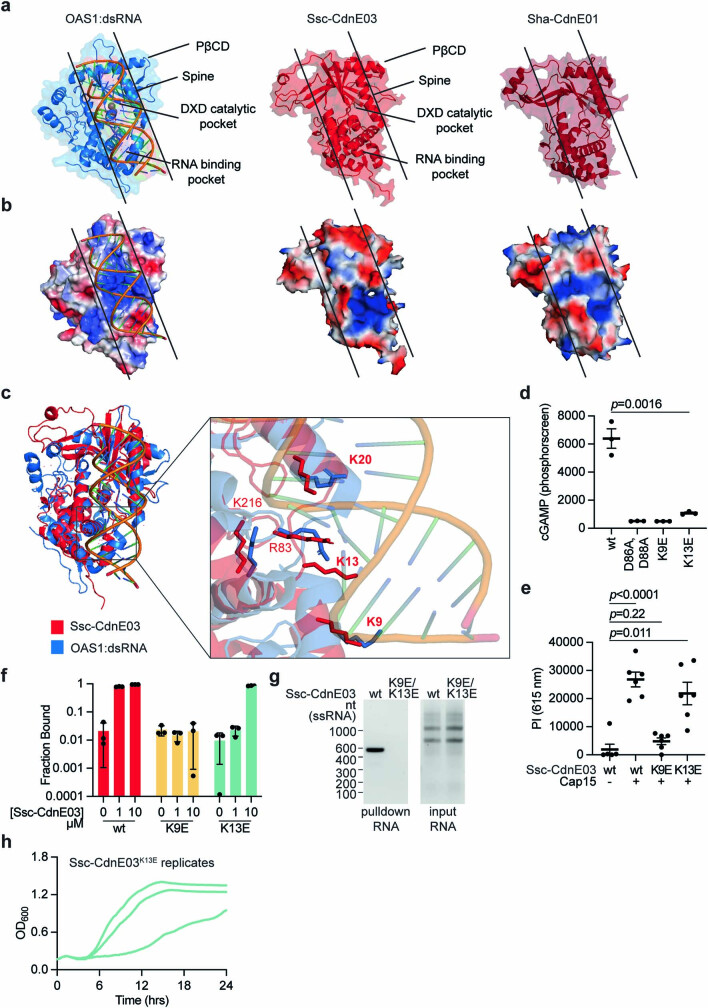
Structural analysis of Ssc-CdnE03. (**a**) Structures of porcine OAS1 bound to dsRNA (PDB: 4RWO), Ssc-CdnE03 and Sha-CdnE01 (AlphaFold rank #1 models). Black lines define a conserved primary dsRNA-binding surface present in OAS1 that seems conserved in the CBASS cyclases. (**b**) Surface electrostatics of the structures shown in (**a**); blue and red, positive and negative charge, respectively. Black lines define the dsRNA-binding surface. (**c**) Structural alignment of Ssc-CdnE03 (red) and crystal structure of porcine OAS1:dsRNA (PDB: 4RWO) (blue) with zoomed-in cutaways highlighting conservation of the active site (top inset) and positively charged residues within the ligand binding surface (bottom inset). (**d**) Quantification of the cyclase reaction products of triplicates of the experiment shown in Fig. 4d. *p* values determined using a *t*-test (unpaired, two-tailed), individual data points are shown with error bars representing the mean +/− s.e.m of n = 3 technical replicates representative of three independent experiments. (**e**) Quantification of propidium iodide (PI) fluorescence at 615 nm of *S. aureus* cultures expressing the Ssc-CdnE03 alone, or the full CBASS system harboring a wild-type, K9E or K13E cyclase, 15 min after infection with Φ80α-vir. *p* values determined using a t-test (unpaired, two-tailed). Individual data points are shown with error bars representing the mean +/− s.e.m for n = 6 biological replicates. (**f**) Quantification of the fraction of the cyclase bound to cabRNA in the experiment shown in Fig. 4c. *p* values determined using a *t*-test (unpaired, two-tailed), Individual data points are shown with error bars representing the mean +/− s.e.m n = 3 technical replicates representative of three independent experiments. (**g**) Agarose gel electrophoresis of the input and output RNA obtained after incubation of Ssc-CdnE03, wild-type or K9E/K13E mutant, with total RNA extracted from cells infected with Φ80α-vir. Data are representative of three independent experiments. (**h**) Replicates of the infection growth curve shown in Fig. 4d, for the cultures expressing Ssc-CdnE03^K13E^.

The predicted structure of Ssc-CdnE03 (Fig. 3a and Extended Data Fig. 4a) revealed shared features and organization with mammalian OAS1 and cGAS despite low sequence similarity^33^ (around 20%). In particular, Ssc-CdnE03 and OAS1 share: (1) the common DNA polymerase β-like nucleotidyltransferase superfamily protein fold and conserved active site architecture (Extended Data Fig. 4a); (2) a pocket on the rear of the active site with positive charge (Extended Data Fig. 4b); (3) two positively charged residues (Arg and Lys) at the first helix of the PβCD domain; and (4) surface exposed lysine and arginine residues along the enzyme ‘spine’ (K9, K13, K20 and R83; Fig. 3a and Extended Data Fig. 4c). Given that these conserved positive residues participate in dsRNA sensing by OAS1^3,6,20^, we substituted K9 and K13 in Ssc-CdnE03 (Fig. 3a and Extended Data Fig. 4c) for glutamic acid residues and assayed for cGAMP production in vitro, through TLC analysis, as well as in vivo, by quantifying the membrane disruption caused by Cap15 activation through the measurement of propidium iodide fluorescence at 615 nm of infected cells (Extended Data Fig. 1k). We found that the K9E mutation completely abrogated cyclase activity and K13E substitution substantially impaired the production of cGAMP, both in vitro (Fig. 3b and Extended Data Fig. 4d) and in vivo (Extended Data Fig. 4e). Electrophoretic mobility shift assays (Fig. 3c and Extended Data Fig. 4f) as well as pull-down assays (Extended Data Fig. 4g) demonstrated that the mutations have a similar effect on the binding of the cabRNA. Consistent with these in vitro observations, CBASS immunity against Φ80α-vir was most severely abrogated by the K9E mutation, and mildly reduced in staphylococci carrying the K13E mutant cyclase (Fig. 3d and Extended Data Fig. 4h). These results demonstrate that the cabRNA is bound by a positively charged surface present in Ssc-CdnE03.

## CdnE01 is also activated by the cabRNA

**Extended Data Fig. 5:**
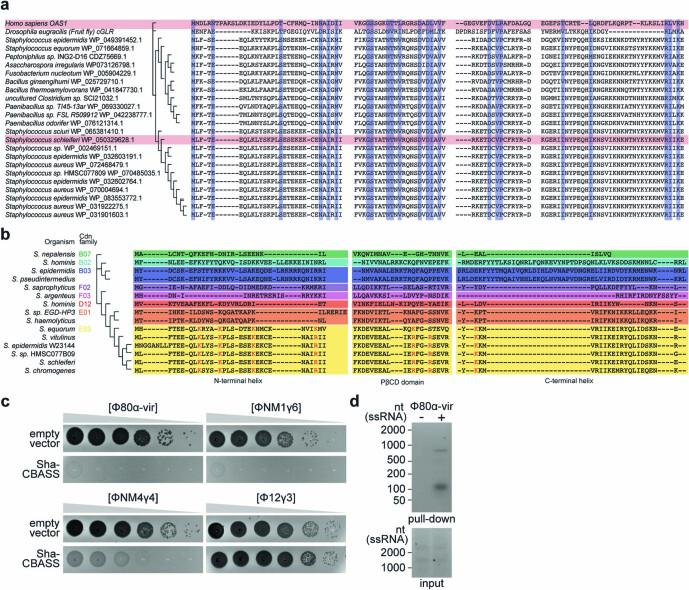
The Sha-CdnE01 cyclase also binds the cabRNA during Φ80α-vir infection. (**a**) Alignment of porcine OAS1, *D. erecta* cGLR, and bacterial CdnE03s. Conserved residues are highlighted in blue. (**b**) Alignment of the N-terminal helix, PβCD and C-terminal domains CD-NTases from staphylococcal species, belonging to different families (in different colors). The conserved basic residues involved in cabRNA binding by Ssc-CdnE03 are highlighted in red. Accession numbers are: *S. nepalensis* WP_096808822.1; *S. hominis* WP_0494417910.1; *S. epidermidis* WP_020363757.1 (CdnB03); *S. pseudintermedius* WP_101458501.1; *S. saprophyticus* WP_041080732.1; *S. argenteus* WP_076688059.1; *S. hominis* WP_071561002.1; *S. sp*. EGD-HP3 WP_021459889.1; *S. haemolyticus* WP_037559004.1; *S. equorum* WP_071664859.1; *S. vitulinus* WP_016911376.1; *S. epidermidis* W23144 (Cd–E03) - EES35648.1; *S. sp*. HMSC077B09 WP_070485035.1; *S. schleiferi* WP_050329628.1; *S. chromogenes* WP_037576868.1. (**c**) Detection of phage propagation after spotting ten-fold dilutions of the lytic DNA phages, Φ80α-vir, ΦNM1γ6, ΦNM4γ4, and Φ12γ3 onto lawns of *S. aureus* RN4220 harboring a plasmid expressing an incomplete (Sha-CdnE01 alone) or intact Sha-CBASS operon. (**d**) Agarose gel electrophoresis of the input and output RNA obtained after incubation of Sha-CdnE01 with total RNA extracted from uninfected staphylococci (RN4220) or from cells infected with Φ80α-vir phage. Data are representative of three independent experiments.

CBASS cyclases that belong to the E03 family are widely distributed in different organisms (Extended Data Fig. 5a). In staphylococci, most CBASS systems belong to the type I class and contain cyclases that are phylogenetically diverse (Extended Data Fig. 5b). Cyclases belonging to the E01 family also contain the conserved lysine or arginine residues that we identified as necessary for the interaction with the cabRNA (Extended Data Fig. 5a). We therefore studied the cyclase from the *Staphylococcus haemolyticus* CBASS system (Sha-CBASS), Sha-CdnE01, which contains this positively charged surface and shares a similar structure with Ssc-CdnE03 (Extended Data Fig. 4a; root mean squared deviation of 0.912 Å over 1,273 atoms; 33% sequence identity). We expressed the Sha-CBASS system in *S. aureus* RN4220 and found that, similar to Ssc-CBASS, it provides immunity against Φ80α-vir and ΦNM1γ6, but not ΦNM4γ4 or Φ12γ3 (Extended Data Fig. 5c). In a pull-down experiment using RNA extracts from staphylococci infected with Φ80α-vir, we found that Sha-CdnE01 interacts with two RNA species made during infection (Extended Data Fig. 5d). RNA-sequencing (RNA-seq) analysis showed that the larger species has the same sequence of the cabRNA pulled down with the Ssc-CdnE03 cyclase, whereas the smaller species is a 70-nucleotide transcript that contains hairpin-1 of the cabRNA (Supplementary Sequences). These results demonstrate that divergent cyclases have evolved to recognize the cabRNA.

## Longer cabRNAs do not activate CdnE03

**Fig. 4:**
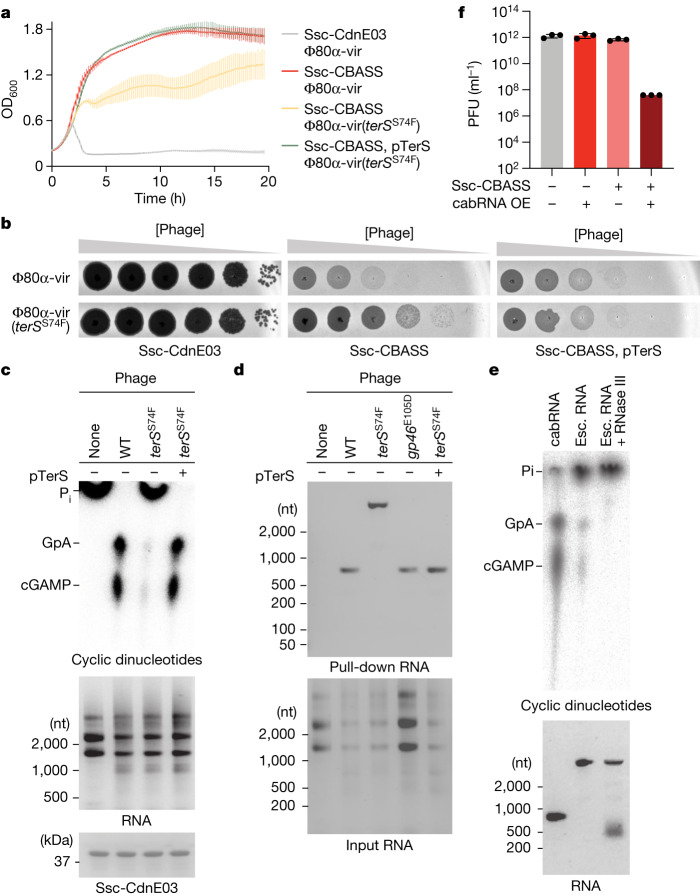
Phage mutants that evade Ssc-CBASS immunity do not produce cabRNA. **a**, Growth of staphylococci harbouring either an incomplete (Ssc-CdnE03 alone) or intact Ssc-CBASS operon measured by optical density at 600 nm after infection with Φ80α-vir or Φ80α-vir(*terS*^S74F^) at a MOI of 1, the latter in the presence or absence of TerS overexpression using plasmid pTerS. Data are mean ± s.d. of three biological replicates. **b**, Detection of phage propagation after spotting tenfold dilutions of Φ80α-vir or Φ80α-vir(*terS*^S74F^) onto lawns of the *S. aureus* RN4220 strains described in **a**. **c**, Thin-layer chromatography analysis of Ssc-CdnE03 products in the presence of total RNA extracted from uninfected staphylococci or cells infected with Φ80α-vir or Φ80α-vir(*terS*^S74F^), the latter in the presence or absence of TerS overexpression using plasmid pTerS. An agarose gel stained with ethidium bromide (middle) and SDS–PAGE stained with Coomassie blue (bottom) are shown as loading controls. Data are representative of three independent experiments. **d**, Agarose gel electrophoresis of the input and output RNA obtained after incubation of Ssc-CdnE03 with total RNA extracted from uninfected staphylococci (RN4220) or from cells infected with Φ80α-vir, Φ80α-vir(*gp46*^E105D^) or Φ80α-vir(*terS*^S74F^), the latter in the presence or absence of TerS overexpression using plasmid pTerS. Data are representative of three independent experiments. **e**, As in **c**, but in the presence of cabRNA, escaper (esc.) RNA, or escaper RNA pre-treated with RNase III. Data are representative of three independent experiments. **f**, Enumeration of plaque-forming units (PFU) from cultures harbouring Ssc-CdnE03 alone (−) or Ssc-CBASS (+) and either an empty vector (−) or a plasmid with cabRNA (+) under the control of an ATC-inducible promoter. OE, overexpression. Individual data points are shown with error bars representing the mean ± s.e.m for *n* = 3 biological replicates.

**Extended Data Fig. 6:**
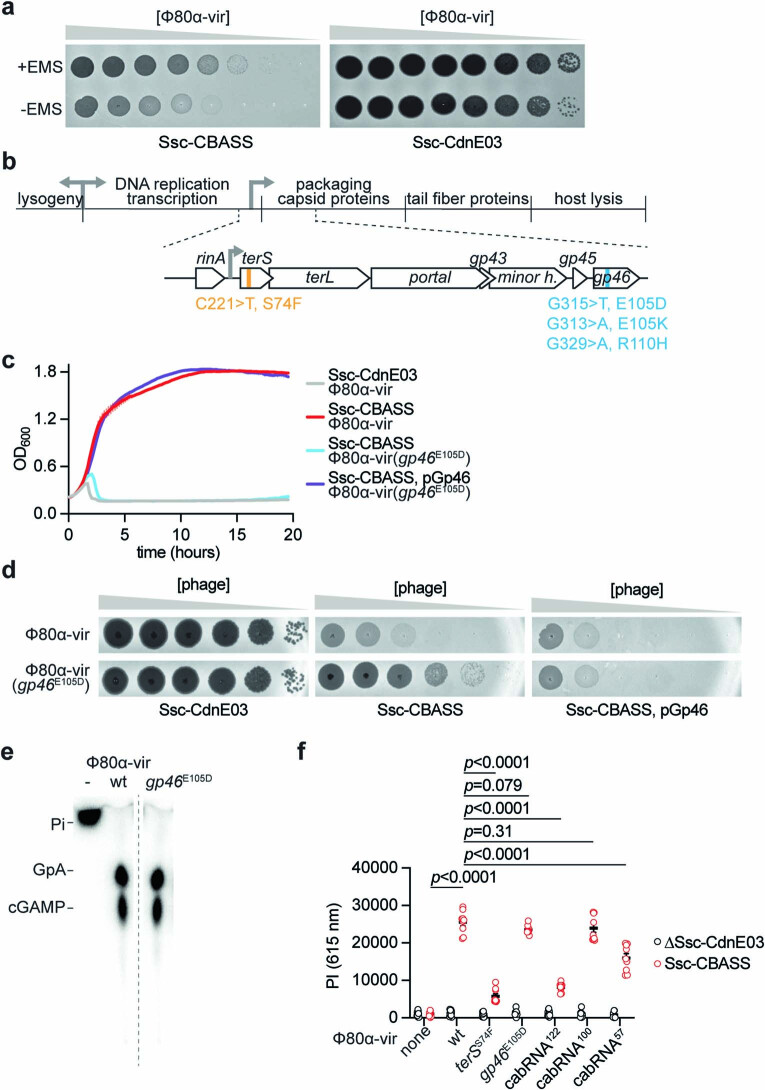
Isolation and characterization of CBASS escapers. (**a**) An overnight culture of *S. aureus* RN4220 was diluted and outgrown to early log-phase, at which time Φ80α-vir at an MOI of 1 was added. Just before the first burst (~30 min), 1% ethyl methanesulfonate (EMS) was added to generate mutations. Infections in the presence of EMS were allowed to proceed at 37 °C for 4 h to allow phage to propagate and lyse the culture. Culture supernatants were collected and used to infect staphylococci expressing Ssc-CBASS to enrich for phage escapers. Supernatants of these cultures were serially diluted and spotted on a lawn of *S. aureus* RN4220::Ssc-CBASS or Ssc-CdnE03. A control experiment without the addition of the EMS mutagen is shown. (**b**) Diagram of Φ80α-vir genome with localization of four unique escaper mutations identified in *terS* or *gp46*. (**c**) Growth of staphylococci harboring either an incomplete (Ssc-CdnE03 alone) or intact Ssc-CBASS operon measured by optical density at 600 nm after the addition of Φ80α-vir or Φ80α-vir(*gp46*^E105D^) at MOI 1. The mean of three biological replicates ± SD is reported. (**d**) Detection of phage propagation after spotting ten-fold dilutions of Φ80α-vir or Φ80α-vir(*gp46*^E105D^) onto lawns of *S. aureus* RN4220 harboring either an incomplete (Ssc-CdnE03 alone) or intact Ssc-CBASS operon, the latter in the presence or absence of Gp46 overexpression using plasmid pGp46. (**e**) Thin-layer chromatography analysis of Ssc-CdnE03 reaction products in the presence of total RNA extracted from uninfected staphylococci or cells infected with wild-type or *gp46*^E105D^ Φ80α-vir. Pi, free phosphates. (**f**) Quantification of propidium iodide (PI) fluorescence at 615 nm of *S. aureus* cultures expressing the Ssc-CdnE03 alone or the full CBASS system, 15 min after infection with wild-type or different mutant versions of Φ80α-vir. *p* values determined using a t-test (unpaired, two-tailed). Individual data points are shown with error bars representing the mean +/− s.e.m *n* = 9 biological replicates.

To gain further insight into the mechanism of Ssc-CBASS induction, we sought to isolate phage mutants that can evade the host defence. Since we were unable to observe discrete Φ80α-vir or ΦNM1γ6 plaques in our assays (Fig. 1a), we used ethyl methanesulfonate (EMS) to introduce random mutations into a Φ80α-vir population and select for variants that are able to form plaques even in the presence of Ssc-CBASS immunity (Extended Data Fig. 6a), and analyse them by next-generation sequencing (Extended Data Fig. 6b). In line with recent findings^34–36^, many of these phages had mutations in the *gp46* gene, which generated missense amino acid substitutions in the scaffold protein for the viral capsid^26^. We first corroborated the evasion of Ssc-CBASS immunity, as well as the restoration of sensitivity in staphylococci expressing wild-type Gp46 (Extended Data Fig. 6c,d). We observed that these phages still activate cGAMP production in vitro and in vivo (Extended Data Fig. 6e,f). In addition, pull-down assays retrieved the same cabRNA isolated from cells infected with wild-type viruses (Fig. 4d). Therefore, we conclude that escapers that produce mutant capsids evade Ssc-CBASS immunity through a mechanism that does not affect the generation of cabRNA or cyclase activation.

**Extended Data Fig. 7:**
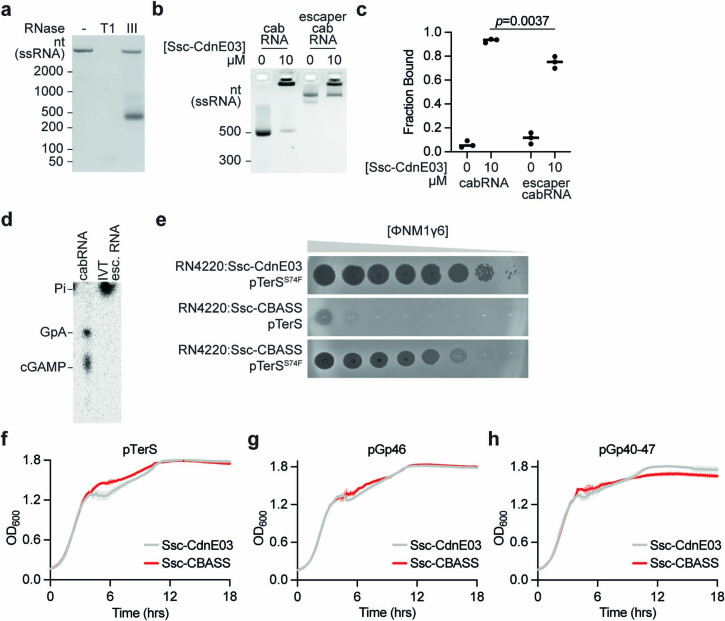
Mechanism of escape mediated by the *terS* mutation. (**a**) Agarose gel electrophoresis of the escaper RNA generated during infection with Φ80α-vir(*terS*^S74F^) isolated from a pull-down assay, treated with RNase T1 or III. Data are representative of three independent experiments. (**b**) Electrophoretic mobility shift assay of cabRNA and the escaper (long) cabRNA pulled down during infection with the Φ80α-vir(*terS*^S74F^) phage, in the presence of increasing concentrations of Ssc-CdnE03. (**c**) Quantification of the fraction of the cyclase bound to cabRNA in the experiment shown in (**b**). *p* values determined using a *t*-test (unpaired, two-tailed), individual data points are shown with error bars representing the mean +/− s.e.m n = 3 technical replicates representative of three independent experiments. (**d**) Thin-layer chromatography analysis of Ssc-CdnE03 reaction products in the presence of cabRNA or in vitro transcribed escaper RNA. Pi, free phosphates. (**e**) Wild-type ΦNM1γ6 was propagated in liquid cultures of *S. aureus* RN4220::Ssc-CBASS harboring a plasmid-borne *terS* gene, wild-type or carrying the S74F mutation. Culture supernatants were collected and serial dilutions were spotted onto lawns of *S. aureus* RN4220::Ssc*-*CdnE03 or RN4220::Ssc*-*CBASS. (**f**) Growth of staphylococci harboring pTerS, providing IPTG-inducible expression of the Φ80α-vir TerS protein, measured by optical density at 600 nm after the addition of the inducer. The mean of three biological replicates ± SD is reported. (**g**) Same as (**f**) but using pGp46 plasmid, providing IPTG-inducible expression of the Φ80α-vir Gp46 protein. (**h**) Same as (**f**) but using pGp40-47 plasmid, providing IPTG-inducible expression of the complete Φ80α-vir viral capsid.

A different escaper mutation mapped to *terS* (also known as *gp40*). This was a C-to-T transition that changes serine 74 (UCU) to phenylalanine (UUU) in the small terminase subunit, located six nucleotides upstream of the cabRNA start. Infection of staphylococci harbouring Ssc-CBASS with the Φ80α-vir(*terS*^S74F^) mutant phage prevented bacterial growth (Fig. 4a) and resulted in the production of high numbers of viral particles (Fig. 4b). cGAMP production was not detected in vitro after incubation of Ssc-CdnE03 with total RNA from infected cultures (Fig. 4c) or in vivo, demonstrated by the lack of Cap15 activation (Extended Data Fig. 6f). Surprisingly, pull-down assays revealed that the cyclase binds an RNA species generated during infection that is several hundreds of nucleotides larger than the cabRNA (Fig. 4d). RNA-seq of this species identified it as a 1,237-nucleotide-long transcript that starts at *rinA* (also known as *gp39*) and extends into *terL* (*gp41*), harbours the C-to-T mutation and shares the same 3′ end with the cabRNA (Fig. 1e and Supplementary Sequences). In contrast to the cabRNA, this species was susceptible to RNase T1, was only partially degraded by RNase III (Extended Data Fig. 7a), and retained a low level of cyclase activation that was eliminated after RNase III treatment (Fig. 4e). Consistent with the pull-down assay, electrophoretic mobility shift assays showed that an IVT escaper RNA was bound by the cyclase (Extended Data Fig. 7b) at similar levels as the cabRNA (Extended Data Fig. 7c), but was unable to induce cGAMP production (Extended Data Fig. 7d). Together, these results indicate that the ‘long’ escaper RNA has a different secondary and tertiary structure than the cabRNA and mediates low levels of Ssc-CdnE03 binding and activation. Finally, we introduced the escape mutation into the *terS* homologue of ΦNM1γ6 and showed that the recombinant phage acquired resistance to Ssc-CBASS (Extended Data Fig. 7e and Supplementary Sequences). Of note, overexpression of wild-type TerS (Extended Data Fig. 7f), Gp46 (Extended Data Fig. 7g) or the complete set of phage genes required for capsid formation, *gp40*–*gp47* (Extended Data Fig. 7h)—whose expression was shown to form Φ80α capsids in the absence of phage infection^37^—were insufficient to activate Ssc-CdnE03.

We also performed experiments to restore Ssc-CBASS immunity against Φ80α-vir(*terS*^S74F^) through plasmid-mediated overexpression of either wild-type TerS (pTerS) or cabRNA (pcabRNA). Protein expression during infection restored cell growth (Fig. 4a) and prevented mutant phage propagation (Fig. 4b) through the generation of a structured cabRNA (Fig. 4c,d). Since the pTerS construct lacks the *terL*-encoded, downstream half of the cabRNA, it cannot produce a transcript that induces cGAMP production. Thus, these data suggest that TerS protein is required for the generation of an activating cabRNA with the proper length. Infection of staphylococci harbouring pcabRNA with Φ80α-vir(*terS*^S74F^) only partially restored Ssc-CBASS immunity (Fig. 4f). In this experiment, both the inactive and active forms of the cabRNA are present in the infected cells, produced by Φ80α-vir(*terS*^S74F^) and pcabRNA, respectively. However, the long cabRNA was not detected in pull-down assays (Extended Data Fig. 3f), a result that suggests that the small binding preference of Ssc-CdnE03 for the short form of the cabRNA (Extended Data Fig. 7c) is amplified when the RNA is overexpressed. Notably, compared with the RNA pulled down in staphylococci harbouring pcabRNA in the absence of Φ80α-vir(*terS*^S74F^) infection, a greater fraction of the pulled-down cabRNA displayed an electrophoretic mobility consistent with its structured form (Extended Data Fig. 3f). These data support our previous hypothesis that the secondary structures present in the inducer RNA, which is required for the activation of Ssc-CdnE03, are generated during phage infection.

## Mutant cabRNAs can activate CdnE03

**Extended Data Fig. 8:**
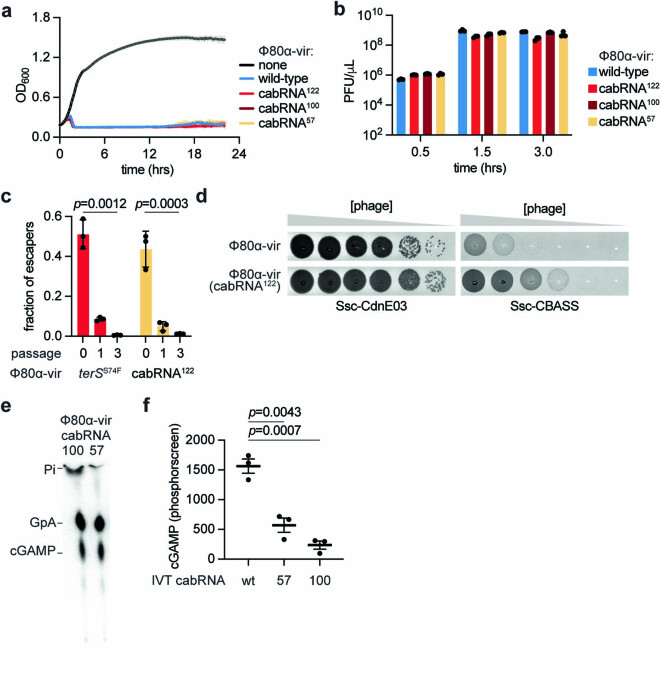
Characterization of Φ80α-vir phages carrying mutations in the cabRNA. (**a**) Growth of staphylococci expressing the Ssc-CdnE03 cyclase alone, measured by optical density at 600 nm after the addition of Φ80α-vir mutants that contain 122, 100 or 57 mutations within the cabRNA sequence, at MOI 1. An uninfected culture is shown as control. The mean of three biological replicates ± SD is reported. (**b**) Enumeration of PFU from cultures shown in (**a**), 0.5, 1.0 and 3.0 h after infection. Mean ± SEM of three biological replicates is reported. (**c**) competition assays between Φ80α-vir and Φ80α-vir(*terS*^S74F^) or Φ80α-vir(cabRNA^122^). A 1:1 ratio mix of both phages was used to infect *S. aureus* RN4220 and supernatants were collected every three hours to enumerate total and Φ80α-vir(*terS*^S74F^) or Φ80α-vir(cabRNA^122^) PFUs (mutant phages were enumerated through plating on lawns of staphylococci expressing the Ssc-CBASS system, on which wild-type phages cannot form plaques), and diluted into a fresh bacterial culture. Each dilution constitutes one passage. *p* values determined using a t test (unpaired, two-tailed), individual data points are shown with error bars representing the mean +/− s.e.m of n = 3 biological replicates. (**d**) Detection of phage propagation after spotting ten-fold dilutions of Φ80α-vir or Φ80α-vir(cabRNA^122^) onto lawns of *S. aureus* RN4220 harboring either an incomplete (Ssc-CdnE03 alone) or intact Ssc-CBASS operon. (**e**) Thin-layer chromatography analysis of Ssc-CdnE03 reaction products in the presence of cabRNA^57^ or cabRNA^100^ isolated from pull-down assays. Pi, free phosphates. (**f**) Quantification of the cyclase reaction products of triplicates of the experiment shown in Fig. 5e. *p* values determined using a *t*-test (unpaired, two-tailed), individual data points are shown with error bars representing the mean +/− s.e.m of n = 3 technical replicates representative of three independent experiments.

We were intrigued by the absence of phage escapers harbouring mutations within the cabRNA sequence, a result that suggests that: (1) mutations in the cabRNA alter the viability of Φ80α-vir (even if they do not alter expression or protein sequence of TerS and TerL); or (2) multiple mutations are required to affect the ability of the cabRNA to induce cGAMP synthesis. In either situation, the mutant phages would be nearly absent in the viral population. To investigate the importance of the cabRNA sequence on phage viability and Ssc-CBASS escape, we mutated the *terS–terL* region of Φ80α-vir that generates the cabRNA, introducing nucleotide substitutions at codon wobble positions that do not disrupt the TerS and TerL protein sequences. We engineered 122 (the maximum number of mutations at the third codon position that are possible without altering the protein sequence or the ribosome binding site for TerL translation), 100 or 57 mutations, generating Φ80α-vir(cabRNA^122^), Φ80α-vir(cabRNA^100^) and Φ80α-vir(cabRNA^57^), respectively (Supplementary Sequences). Although the mutations changed the predicted secondary structure of the cabRNA (Supplementary Sequences), they were tolerated, leading to the generation of viable phages (Extended Data Fig. 8a,b). However, competition assays between Φ80α-vir and Φ80α-vir(cabRNA^122^) showed that the relative titre of the mutant phage decreased over the course of three passages (Extended Data Fig. 8c). We also competed Φ80α-vir(*terS*^S74F^) and obtained similar results. Therefore, these data demonstrate that mutations affecting either the cabRNA sequence or length impair maximal viral propagation on staphylococci.

**Fig. 5:**
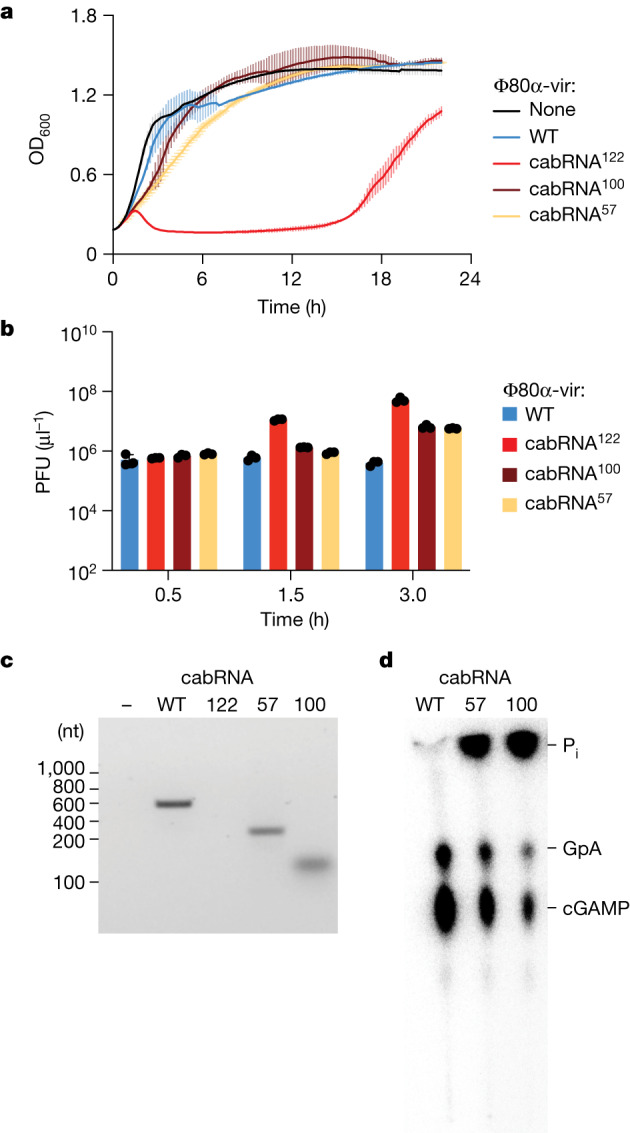
Mutations reduce the length of the cabRNA but do not eliminate its activating properties. **a**, Growth of staphylococci carrying the full Ssc-CBASS locus, measured by optical density at 600 nm after the addition of Φ80α-vir mutants that contain 122, 100 or 57 mutations within the cabRNA sequence at a MOI of 1. An uninfected culture is shown as control. Data are mean ± s.d. of three biological replicates. **b**, Enumeration of PFU from cultures shown in **a**, 0.5, 1.0 and 3.0 h after infection. Data are mean ± s.e.m. of three biological replicates. **c**, Agarose gel electrophoresis of the RNA obtained after incubation of Ssc-CdnE03 with no RNA (−) or total RNA extracted from staphylococci infected with wild-type or cabRNA mutants of the Φ80α-vir phage. Data are representative of three independent experiments. **d**, Thin-layer chromatography analysis of Ssc-CdnE03 products in the absence of RNA (−) or in the presence of the IVT cabRNA (wild type), cabRNA^57^ and cabRNA^100^. A representative image of multiple replicates is shown.

To investigate the effect of the cabRNA mutations on immunity, we infected staphylococci carrying the Ssc-CBASS system and found that Φ80α-vir(cabRNA^100^) and Φ80α-vir(cabRNA^57^) were able to induce a partial immune response, resulting in intermediate levels of bacterial growth (Fig. 5a) and phage propagation (Fig. 5b), when compared with the wild-type virus. By contrast, Φ80α-vir(cabRNA^122^) completely escaped CBASS immunity (Fig. 5a,b), although the plaques formed on lawns of staphylococci carrying Ssc-CBASS were smaller than those formed on lawns of a control strain (Extended Data Fig. 8d), a result that suggests the presence of low levels of immunity against Φ80α-vir(cabRNA^122^). Of note, when using RNA extracted from cells infected with Φ80α-vir(cabRNA^100^) or Φ80α-vir(cabRNA^57^), the cyclase pulled down RNAs that are smaller than the cabRNA produced during infection with wild-type phage (Fig. 5c), which were able to activate cGAMP synthesis in vitro and in vivo (Extended Data Fig. 8e,f). RNA-seq determined the presence of 200 and 80 nucleotide-long species produced during Φ80α-vir(cabRNA^57^) and Φ80α-vir(cabRNA^100^) infection, respectively, which corresponded with the unmutated regions of the original cabRNA (Supplementary Sequences). Synthetic RNAs containing these sequences also activated Ssc-CdnE03 in vitro (Fig. 5d and Extended Data Fig. 8f). By contrast, no RNA was recovered during the pull-down of RNAs extracted from staphylococci infected with Φ80α-vir(cabRNA^122^) (Fig. 5c), a result that is consistent with the lack of Cap15 activation (Extended Data Fig. 6f) and the CBASS escape phenotype.

## Phages generate diverse cabRNAs

**Fig. 6:**
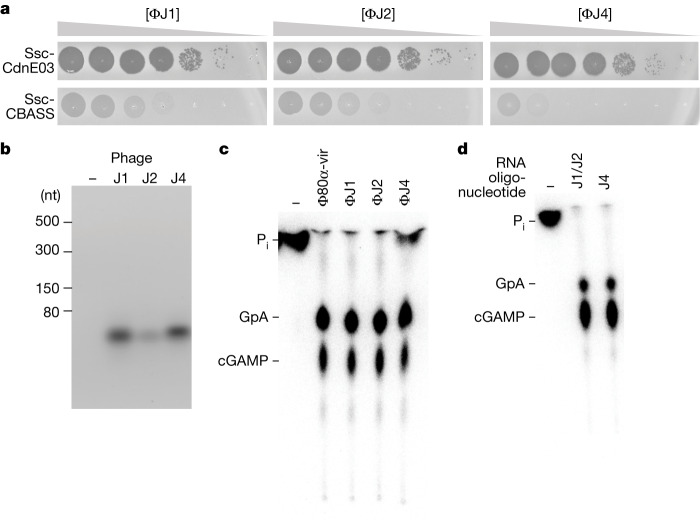
Diverse staphylococcal phages produce different cabRNAs to initiate the Ssc-CBASS response. **a**, Detection of phage propagation after spotting tenfold dilutions of the lytic DNA phages ΦJ1, ΦJ2 and ΦJ4 onto lawns of *S. aureus* RN4220 harbouring a plasmid expressing an incomplete (Ssc-CdnE03 alone) or intact Ssc-CBASS operon. **b**, Agarose gel electrophoresis of the RNA obtained after incubation of Ssc-CdnE03 with total RNA extracted from uninfected staphylococci (−) or from cells infected with ΦJ1, ΦJ2 or ΦJ4. Data are representative of three independent experiments. **c**, Thin-layer chromatography analysis of Ssc-CdnE03 products in the absence of RNA (−) or in the presence the cabRNAs pulled down in the experiment shown in **b**. A representative image of multiple replicates is shown. **d**, As in **c**, but incubating the cyclase with synthetic oligonucleotides with the sequences of the cabRNAs produced by the ΦJ1, ΦJ2 and ΦJ4 phages.

**Extended Data Fig. 9:**
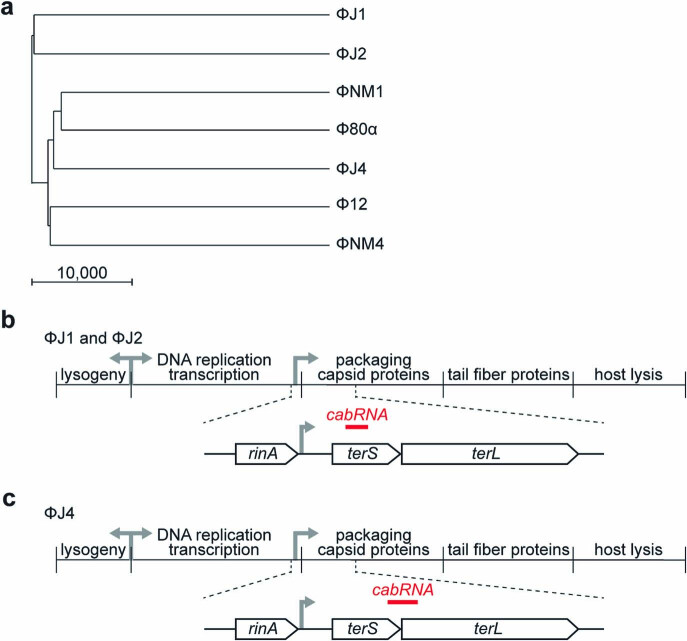
Characterization of Ssc-CBASS immunity against ΦJ1, ΦJ2 and ΦJ4 phages. (**a**) Phylogenetic tree of the phages used in this study, generated using Jalview. The scale bar indicates the genetic distance calculated by this software. (**b**) Diagram of the ΦJ1/2 genomes showing the localization of the cabRNA sequence within the *terS/L* region. (**c**) Same as (**b**) but for the ΦJ4 genome.

We isolated additional phages from different *S. aureus* clinical strains and tested them for their ability to induce cGAMP production by Ssc-CdnE03. We found three novel phages, ΦJ1, ΦJ2 and ΦJ4, that are more phylogenetically divergent to Φ80α-vir than ΦNM1γ6 (Extended Data Fig. 9a) and were restricted by the Ssc-CBASS system (Fig. 6a). Pull-down assays using RNA from cells infected with these phages led to the isolation of cabRNAs less than 50 nt long (Fig. 6b) that were able to induce cGAMP production in vitro (Fig. 6c). RNA-seq of these cabRNAs showed that they are transcribed from *terS* in the case of the ΦJ1 and ΦJ2 phages, which share identical *terS* sequences (Extended Data Fig. 9b), and from the *terS–terL* boundary for ΦJ4 (Extended Data Fig. 9c). None of these cabRNAs have sequence homology to each other or to the Φ80α-vir and ΦNM1γ6 cabRNA, but in all cases they are predicted to form hairpins (Supplementary Sequences). Finally, we obtained synthetic versions of the ΦJ1/2 and ΦJ4 cabRNAs and found that both induced cGAMP production in vitro (Fig. 6d). These results expand our previous findings to diverse phages and demonstrate a conservation of the region from which the cabRNAs are produced—that is, the *terS* and *terL* genes.

## Discussion

Here we show that RNAs produced by staphylococcal phages during infection—cabRNAs—trigger CBASS immunity through the activation of cyclic nucleotidyltransferases from the E clade. We found that although cabRNAs are transcribed from the viral *terS* and *terL* genes and are predicted to form secondary structures, they can have different lengths and sequences. For the Φ80α-vir and ΦNM1γ6 phages, the cabRNA length is 400 nt and contains secondary structures that are important for its activating properties. The size of the Ssc-CdnE03 positively charged surface (approximately 40 Å in length) should be able to only accommodate a dsRNA of approximately 20 base pairs (Supplementary Discussion File). A similar observation has been reported for the human antiviral 2′,5′-oligoadenylate synthetase (OAS) enzymes OAS1 and OAS3, which also require large dsRNA molecules for optimal activity^38^. In OAS1, the positively charged surface can interact with approximately 18–20 base pairs (Extended Data Fig. 4a,b), yet dsRNAs of this length can only provide limited activity^38^ (around 7 to 8% of the maximum). The other two cabRNAs isolated in this study have lengths (34 and 49 nt) that would make them optimally fit the size of Ssc-CdnE03 binding area.

To prevent the rapid evolution of phages that avoid recognition, defence systems are usually triggered by processes that are essential for infection, where deletion or mutation causes a considerable fitness cost for the virus^39^. The cabRNA seems to have these characteristics as well, possibly a consequence of its origin as a transcript from the *terS–terL* gene region, which is essential for the viral lytic cycle. In the case of the Φ80α-vir cabRNA, we found that phages carrying mutations that increase the length or mutate the sequence of the cabRNA in Φ80α-vir(*terS*^S74F^) and Φ80α-vir(cabRNA^122^), respectively, do not activate CBASS immunity (Fig. 4a,b and Fig. 5a, b) and are viable. However, they exhibit a substantial fitness loss when competed with the wild-type phage (Extended Data Fig. 8c). In addition, Φ80α-vir(cabRNA^100^) and Φ80α-vir(cabRNA^57^) phages, which have extensive sequence alterations in the cabRNA, produce smaller cabRNA molecules (Fig. 5c) that can trigger the Ssc-CBASS response (Fig. 5a,b,d). Although it is unclear how the mutations reduce the cabRNA length, this result demonstrates that the cabRNA constitutes a target molecule with strong immunogenicity that must accumulate a high number of mutations to lose its cyclase-activating properties. The evolutionary difficulty of such mutagenic events is perhaps the reason why additional routes of escape have been found^34–36^, namely mutations in capsid-related proteins (Gp46 in the case of Φ80α-vir). Indeed, we found that the Φ80α-vir(*gp46*^E105D^) escaper phage still produces a structured cabRNA (Fig. 4d) that is capable of inducing cGAMP synthesis by the Ssc-CdnE03 cyclase (Extended Data Fig. 6e) and activating Cap15 (Extended Data Fig. 6f). However, in contrast to the *terS*^S74F^ mutant phages, the *gp46*^E105D^ escapers completely kill staphylococci in liquid cultures (Extended Data Fig. 6c, compared to Fig. 4a) and form larger plaques than Φ80α-vir(*terS*^S74F^) phages (Extended Data Fig. 6d, compared to Fig. 4b). Therefore, we hypothesize that the low frequency and/or high fitness cost of cabRNA mutations that disrupt its recognition by CBASS cyclases (and possibly also mutations in other cyclase activators), led to the rise of capsid mutations that interfere with CBASS immunity after cGAMP production during the evolutionary arms race between bacteria and their phages.

Eukaryotic synthases are activated by long, unmodified dsRNA in a sequence-independent manner, since the presence of these molecules in the cytoplasm usually signals infection by RNA viruses^16^. Given the lack of nuclear compartmentalization in bacteria, our results demonstrate that prokaryotic CBASS cyclases require a specific phage-derived RNA with special properties that distinguish it from other host-derived transcripts to avoid the induction of autoimmunity. Future studies will focus on these special properties of the cabRNA, such as its biogenesis, structure, function and molecular interactions with CBASS cyclases, that enable it to initiate the synthesis of cyclic nucleotide second messengers.

## Methods

### Bacterial strains and growth conditions

The bacterial strains used in this study are listed in Supplementary Table 1. *S. aureus* strain RN4220^25^ was grown at 37 °C with shaking (220 RPM) in brain heart infusion (BHI) broth, supplemented with chloramphenicol (10 μg ml^−1^) or erythromycin (10 μg ml^−1^) to maintain pC194-based^24^ or pE194-based plasmids^40^, respectively. Cultures were supplemented with chloramphenicol (5 μg ml^−1^) to select for strains with chromosomally integrated Ssc-CBASS or Ssc-CdnE03. Gene expression was induced by the addition of 1 mM isopropyl-d-1-thiogalactopyranoside (IPTG) or 100 ng ml^−1^ ATC, where appropriate.

### Bacteriophage propagation

The bacteriophages used in this study are listed in Supplementary Table 2. To generate a high-titre phage stock, an overnight culture of *S. aureus* RN4220 was diluted 1:100 and outgrown to mid-log phase (~90 min) in BHI broth supplemented with 5 mM CaCl_2_. The culture was diluted to an OD_600_ of 0.5 (~1 × 10^8^ CFU ml^−1^). The culture was infected by adding phage at a MOI of 0.1 (~1 × 10^7^ PFU ml^−1^), or by inoculating with either a single picked plaque or scrape of a frozen stock. The infected culture was grown at 37 °C with shaking and monitored for lysis (full loss of turbidity was typically observed at ~3–4 h). Culture lysates were centrifugated (4,300*g* for 10 min) to pellet cellular debris. The supernatant was collected, passed through a sterile membrane filter (0.45 μm), and stored at 4 °C. Phage concentrations were determined by serially diluting the obtained stock in tenfold increments and spotting 5 μl of each dilution on BHI soft agar mixed with RN4220 and supplemented with 5 mM CaCl_2_. After incubation overnight at 37 °C, individual plaques (that is, zones of no bacterial growth) were counted, and the viral titre was calculated.

### Molecular cloning

The plasmids (and details of their construction) and the oligonucleotide primers used in this study are listed in Supplementary Tables 3 and  4, respectively. The coding sequences of Ssc-CBASS and phage gene products were obtained from genomic DNA preparations of *S. schleiferi* 2142-05 cultures^22^ or phage stocks^41^, respectively.

### Chromosomal integration of Ssc-CBASS

Ssc-CBASS or Ssc-CdnE03, along with a chloramphenicol resistance (cmR) cassette, was integrated into the *hsdR* gene (which encodes the defective R-subunit of the restriction-modification system in *S. aureus* RN4220), an insertion site which was previously shown to not impact growth^42^. Ssc-CBASS-cmR and Ssc-CdnE03-cmR were amplified from the plasmids pDVB303 and pDVB301 respectively, using primers oDVB565 and oDVB566, which were flanked with *loxP* sites at both ends followed by 60-bp homology regions to *hsdR*. Electrocompetent *S. aureus* RN4220 cells harbouring the recombineering plasmid pPM300 were electroporated with 1−2 μg of PCR product and selected for with chloramphenicol (5 μg ml^−1^). Potential integrants were screened by colony PCR as well as for functional immunity, and then verified by Sanger sequencing.

### Isolation of strictly lytic phage mutants

To construct a virulent mutant of the phage Φ80α^26^, we used a variation of a method previously described to generate ΦNM1γ6^27^, ΦNM4γ4^28^ and Φ12γ3^29^. Φ80α-vir was isolated as a spontaneous escaper forming a clear plaque following Φ80α infection of a BHI soft-agar lawn of *S. aureus* RN4220 cells harbouring plasmid pDVB08, which encodes a type III-A CRISPR–Cas system targeting the Φ80α cI-like repressor. PCR of the Φ80α-vir *cI* gene and Sanger sequencing confirmed an 8-bp deletion.

### Isolation of ΦJ1, ΦJ2, and ΦJ4

*S. aureus* strains NRS52, NRS102, and NRS110 from the Network on Antimicrobial Resistance in *S. aureus* (NARSA) repository (BEI/NIAID) were grown overnight at 37 °C with shaking (200 RPM) in Mueller Hinton II (MHII) broth. The next day, cultures were diluted 1:100 into 10 ml fresh MHII and grown for one hour to enter early log phase. Prophages were then induced by adding ciprofloxacin at a final concentration of 0.8 mg ml^−1^ to each culture. Following a 4 h incubation at 37 °C, each culture was spun down, and the supernatants filtered through a 0.22 μm syringe-driven filter. Singles plaques of these filtrates were obtained via serial dilution onto lawns of *S. aureus* RN4220, and high-titre phage stocks were produced as described above.

### Soft agar phage infection

One-hundred microlitres of an overnight bacterial culture was mixed with 5 ml BHI soft agar supplemented with 5 mM CaCl_2_ and poured onto BHI agar plates to solidify at room temperature (~15 min). Phage lysates were serially diluted tenfold and 4 μl was spotted onto the soft-agar surface. Once dry, plates were incubated at 37 °C overnight and visualized the next day. Individual plaques (zones of no bacterial growth) were counted manually.

### Liquid culture phage infection

Overnight cultures were diluted 1:100 in BHI supplemented with 5 mM CaCl_2_ and the appropriate antibiotic for selection, outgrown at 37 °C with shaking to mid-log phase (~90 min), and normalized to OD_600_ 0.5. For the desired MOI, a calculated volume of phage stock was added to each culture and 150 μl was seeded into each well of a 96-well plate. OD_600_ was measured every 10 min in a microplate reader (TECAN Infinite 200 PRO) at 37 °C with shaking.

### RT–qPCR

Total RNA was extracted from *S. aureus* cells using a Direct-Zol RNA MiniPrep Plus Kit (R2072). Extracted RNA was treated with TURBO DNase (Thermo Fisher Scientific) before cDNA first-strand synthesis with SuperScript IV Reverse Transcriptase (Thermo Fisher Scientific) using random hexamers. qPCR was performed using Fast SYBR Green Master Mix (Life Technologies) and 7900HT Fast Real-Time PCR System (Applied Biosystems) with primer pairs for the *S. aureus* housekeeping gene *ptsG* (oDVB426/427), *cdnE03* (oDVB610/611) or *cap15* (oDVB614/615).

### Protein expression and purification

Ssc-CdnE03, Sha-CdnE01, and various mutants were expressed and purified using the following approach: transformed BL21 (DE3) *E. coli* were grown in LB broth at 37 °C with shaking to mid-log phase (OD_600_ 0.6–0.8), at which point the culture was cooled on ice for 10 min and induced with 0.2 mM IPTG for 16 h at 18 °C. Bacteria were harvested, resuspended in lysis buffer (25 mM Tris pH 7.4, 300 mM NaCl, 5% glycerol, 2 mM β-mercaptoethanol), and subjected to a single freeze–thaw cycle. The cells were incubated on ice with lysozyme, DNase I, and EDTA-free protease inhibitor cocktail. After incubating on ice for 40 min, the cells were lysed using sonication. Lysates were clarified by centrifugation and applied to cobalt affinity resin. After binding, the resin was washed extensively with lysis buffer prior to elution with lysis buffer containing 300 mM imidazole. Eluted proteins were then proteolysed with TEV protease to remove the affinity tag during overnight 4 °C dialysis to reaction buffer (25 mM HEPES-KOH pH 7.5, 250 mM KCl, 5% glycerol, 2 mM β-mercaptoethanol). The cleaved proteins were then passed over cobalt resin to collect the remaining tag (or uncleaved protein) and concentrated using 10,000 MWCO centrifugal filters (Amicon). Purified proteins were visualized by SDS–PAGE and used for downstream in vitro assays.

### Nucleotide synthesis assays

Nucleotide synthesis assays were performed using a variation of the method described by Whiteley et al.^8^. The final reactions (50 mM 3-(cyclohexylamino)-2-hydroxy-1-propanesulfonic acid (CAPSO) pH 9.4, 50 mM KCl, 5 mM magnesium acetate, 1 mM DTT, 25 or 250 uM individual NTPs, trace amounts of [α-^32^P]NTP, 5 uM nucleic acid ligand, and 5 μM enzyme) were started with the addition of enzyme. All reactions except for those with RNA activator (2 h) were incubated overnight at 37 °C. For reactions with total RNA extracts, 500 ng was added to each condition. The sequences of the ssRNA oligonucleotides used as activators are reported in Supplementary Table 5. Reactions were stopped with the addition of 1 U of alkaline phosphatase, which removes triphosphates on the remaining NTPs and enables the visualization of cyclized nucleotide species. After a 1 h incubation, 0.5 μl of the reaction was spotted 1.5 cm from the bottom of a PEI-cellulose thin-layer chromatography (TLC) plate, spaced 0.8 cm apart. TLC plates were developed in 1.5 M KH_2_PO_4_ pH 3.8 until the buffer front reached 1 cm from the top (~ 12 cm). The TLC plates were completely dried, covered with plastic wrap and exposed to a phosphor screen before detection by a Typhoon Trio Imager System.

For the putative activator screening in vitro, RN4220 cells were lysed using lysostaphin (5 mg ml^−1^) treatment at 37 °C for 1 h, clarified lysate was then added to nucleotide synthesis reactions. Phage particles were enriched using polyethylene glycol (PEG8000) precipitation. Resuspended phage was then treated with DNase and RNase to ensure that only phage structural elements remained. Genomic DNA from RN4220 was prepared according to the Wizard Genomic DNA Purification Kit (A1120). Genomic DNA of phage was isolated following phage particles purification, using polyethylene glycol precipitation and CsCl gradient, according to the manufacturer’s protocol. Total RNA was extracted from *S. aureus* cells with or without infection using a Direct-Zol RNA MiniPrep Plus Kit (R2072).

To purify the Ssc-CdnE03 cyclic nucleotide product for mass spectrometry analysis, nucleotide synthesis reaction conditions were scaled up to 1 ml reactions containing 5 μM Ssc-CdnE03, 250 uM ATP, 250 uM GTP, approximately 5 ng of cabRNA, in 50 mM CAPSO pH 9.4, 50 mM KCl, 5 mM Mg(OAc)_2_, 1 mM DTT buffer. Reactions were incubated with gentle shaking for 24 h at 37 °C followed by Quick CIP (NEB) treatment for 4 h at 37 °C. Following incubation, reactions were filtered through a 10,000 MWCO centrifugal filter (Amicon) to remove protein.

### Nucleotide high-resolution mass spectrometry analysis

All solvents and reagents used for chromatography were liquid chromatography–mass spectrometry grade. Ultrahigh performance liquid chromatography–high-resolution mass spectrometry (UPLC-HRMS) data were acquired on a Sciex ExcionLC UPLC coupled to an X500R mass spectrometer, controlled by SCIEXOS software. Chromatography was carried out on a Waters XBridge BEH Amide Column XP (2.1 × 150 mm, 2.5 µm), under the following conditions: 100% B from 0.0 to 1.0 min, from 100% to 70% B from 1.0 to 8.9 min, 60% B from 9.0 to 13.0 min, 100% B from 13.2 to 20.0 (A: 10 mM ammonium formate + 0.1% formic acid; B: 90% acetonitrile in 10 mM ammonium formate + 0.1% formic acid buffer), with a flow rate of 0.40 ml min^−1^ and 0.5 µl of injection volume. HRMS analysis were performed in positive and negative electrospray ionization mode in the range *m/z* 100–1,200 for MS^1^ and MS^2^ scans; the maximum candidate ions subjected for Q2-MS^2^ experiments was 7, declustering potential of 80 V, collision energy of 5 V and temperature of 500 °C. For electrospray ionozation high-resolution mass spectrometry (ESI–HRMS) experiments the spray voltage was set in 5,500 V, the Q2 collision energy at 30 V with a spread of 10 V, whereas the spray voltage for ESI–HRMS was set in 4500 V and the Q2 collision energy at 35 V with a spread of 10 V. The concentration of the standard solutions was 6.25 µM, and all the solutions were centrifuged (13,000 rpm × 3 min) before injection. The molecular ions for ESI modes were analysed for all compounds, but the fragmentation in ESI mode showed a better consistency and was consequently used for the structural analysis. The data analysis was carried out with MestReNova software (14.3.0), data output was converted with MSConvert from Proteowizard, MS^2^ mirror plot was obtained from GNPS using averaged MS^2^ spectra from GNPS molecular networking.

### Nuclease P1 cleavage analysis

Nuclease P1 cleavage analysis was performed using Ssc-CdnE03 reactions labelled with α-^32^P-ATP or α-^32^P-GTP. Radiolabelled nucleotide products were incubated with nuclease P1 (80mU; N8630, Sigma) in buffer (30 mM sodium acetate pH 5.3, 5 mM ZnSO_4,_ and 50 mM NaCl) for 30 min at 37 °C in the presence of Quick CIP (NEB). Reactions were terminated by heat inactivation at 95 °C for 2 min before PEI-cellulose TLC analysis as described above.

### RNA extraction from phage infection

Ten millilitres of a mid-log phase *S. aureus* RN4220 culture normalized to OD_600_ 0.5 was infected with phage at MOI 10. Infection was allowed to proceed for 30 min, just before the completion of the first burst. Cells were pelleted at 4,300*g* for 5 min and flash-frozen with liquid nitrogen. The pellet was resuspended in 150 μl PBS and 50 μl lysostaphin (5 mg ml^−1^) and incubated at 37 °C for 30 min. Total RNA was extracted from *S. aureus* cells using a Direct-Zol RNA MiniPrep Plus Kit (R2072). In brief, 450 μl Trizol was added to the lysate, vigorously vortexed and centrifuged at 16,000*g* for 30 s. In total, 650 μl of 100% ethanol was added to the supernatant and the samples were thoroughly vortexed. The entire volume was passed through a Zymo-Spin IIICG Column followed by in-column treatment with DNase I for 15 min at room temperature. The column was washed according to the manufacturer’s protocol and RNA was eluted in 100 μl nuclease-free water.

### RNA pull-down assay

His_6_–MBP-tagged or His_6_-tagged Ssc-CdnE03 as well as Sha-CdnE01 were expressed and purified as described above. Purified His_6_–MBP tag alone was prepared alongside as a negative control. After immobilizing ~0.2 mg of protein on cobalt resin, the column was washed extensively with lysis buffer prior to the addition of 5 ml of lysis buffer containing 1 mM MgCl_2_, 5 units of RNaseOUT (ThermoFisher, 10777019), and 100 μg of total RNA extracted from cultures with or without phage infection. The RNA was incubated with the tagged Ssc-CdnE03 on the column for 40 min before washing the column with 5 volumes of lysis buffer. The column was treated with His_6_-tagged TEV protease to release the Ssc-CdnE03 and bound RNA. Eluted protein was collected for each sample and combined with TRI Reagent (Zymo Research, R2050-1-200). RNA was then extracted according to the Direct-Zol RNA MiniPrep Plus Kit (R2072) manufacturer’s protocol. The final RNA product was run on a 2% agarose 1× TAE gel and stained with SyBr Gold or ethidium bromide. Eluted protein samples were collected as controls for visualization by SDS–PAGE.

### RNA sequencing

cDNA library preparation was performed using the Illumina TruSeq Stranded mRNA (for >100 nt) or Small RNA (for <100 nt) library preparation kits. In brief, reverse transcription of the RNA isolated from the CD-NTase pull-down assays was performed using the Illumina manufacturer’s protocol, or alternatively as follows: RNA was treated with TURBO DNase (Thermo Fisher Scientific) before cDNA first-strand synthesis with SuperScript IV Reverse Transcriptase using random hexamers. Second-strand synthesis of the cDNA was performed with Q5 DNA polymerase at 15 °C for 2 h, followed by 75 °C for 10 min in the presence of RNase H and DMSO. Chemical fragmentation was then performed using the Illumina manufacturer’s protocol, or alternatively as follows: cDNA was sheared to 150-bp fragments using an S220 Covaris Focused-Ultrasonicator (peak incident power: 175 W, duty factor: 10%, cycles per burst: 200, treatment time: 430 s, temperature 4 °C) in S-Series Holder microTUBEs (PN 500114). Quantification and quality check of cDNA libraries were performed by Qubit 4.0 Fluorometer and Agilent Bioanalyzer/Tapestation, respectively. 12 pM of indexed cDNA libraries was loaded on an Illumina MiSeq instrument for either single-read (150 cycle) or paired-end sequencing (2 ×75 cycle). Bowtie2 via the Galaxy open-source interface^43^ was used to align sequencing reads to phage and host genomes and then visualized using Geneious Prime. A custom Python script was used to convert the output SAM alignments into CSV files containing the number of aligned reads at each nucleotide location along a given reference genome.

### RNA structure prediction

RNA secondary structures were analysed using the ViennaRNA 2.0 package^31^ and visualized via the SnapGene interface.

### In vitro transcription of cabRNA

IVT was performed according to the Thermo Scientific TranscriptAid T7 High Yield Transcription Kit protocol (K0441). Linear dsDNA for the cabRNA, and *terS*^*S74F*^ phage escaper RNA sequences were PCR-amplified using oCR190/193 (sense cabRNA), oCR191/192 (antisense cabRNA), and oDVB691/oCR193 (*terS*^*S74F*^ phage escaper RNA). The target sequence was placed downstream of a T7 promoter, which was inverted for antisense transcription reactions. For high yield in vitro transcription reactions, 1 μg of PCR product was combined with TranscriptAid Enzyme mix and NTPs. Following a 4 h incubation period at 37 °C, transcripts were purified according to the Direct-Zol RNA MiniPrep Plus Kit (R2072) manufacturer’s protocol. To stimulate the refolding and formation of a structured RNA product, the purified IVT samples were heated at 95 °C for 5 min in a heat block, which was slowly cooled down to room temperature over 1 h. Where indicated, IVT products were either heat-treated (folded) or untreated.

### Electrophoretic mobility shift assay

Analysis of in vitro protein-nucleic acid complex formation was performed as previously described^18^. 1 μM cabRNA or escaper RNA was incubated with Ssc-CdnE03 at a concentration of 0, 1, or 10 μM. Complex formation was performed in the reaction buffer: 50 mM CAPSO pH 9.4, 50 mM KCl, 5 mM magnesium acetate, 1 mM DTT. Reactions (20 μl) were incubated at 4 °C for 25 min before separation on a 2% agarose gel using 1× TB buffer as running buffer. The agarose gel was stained with ethidium bromide and complex formation was visualized using an Amersham ImageQuant 800 (Cytiva). Fraction of RNA bound in each sample was calculated by dividing the mean intensity of the shifted band over the sum of the mean intensity for the shifted and unshift bands. Mean intensity of signal was generated using Fiji (measure tool).

### In vivo Ssc-Cap15 activation assay

Overnight cultures of *S. aureus* RN4220 harbouring either Ssc-CdnE03 alone or the full Ssc-CBASS operon were diluted 1:100 in BHI supplemented with 5 mM CaCl_2_, 10 μM propidium iodide (PI), and the appropriate antibiotic for selection, outgrown at 37 °C with shaking to mid-log phase (~90 min), and then normalized to OD_600_ 0.5. Cells were then infected with phage at MOI 10 and allowed for grow at 37 °C for another 10 min before collection (4,000 rpm, 5 min), and resuspension of 2 × 10^8^ cells in 200 μl 1× PBS. Upon binding to DNA or RNA in cells propidium iodide fluorescence is enhanced 20 to 30-fold Excitation_max_ = 540 nm/Emission_max_ = 617 nm, a process which requires disruption of the bacterial membrane. Thus, cGAMP-induced membrane disruption by Ssc-Cap15 was measured by adding cells to a 96-well plate and using a multi-well fluorescence scanner to report emission in each well at 615 nm. Experiments were performed with six independent clones (biological replicates), each with three technical replicates.

### Structural prediction and analysis of Ssc-CdnE03

The amino acid sequence of Ssc-CdnE03 sequence was used to seed a position-specific iterative BLAST (PSI-BLAST) search of the NCBI non-redundant protein and conserved domain databases (composition-based adjustment, E-value threshold 0.01). Putative domains identified from this search include a C-terminal nucleotidyltransferase (NT) domain of 2′,5′-oligoadenylate (2–5 A) synthetase (NT_2-5OAS) domain (residues 61–204; E-value 2.63 × 10^−15^) and an N-terminal tRNA nucleotidyltransferase (CCA-adding enzyme) domain (residues 5–158; E-value 8.01 × 10^−4^). A structure of the Ssc-CdnE03 was predicted using AlphaFold (ColabFold). Following structure determination, pairwise structural comparison of the rank 1 model to the full PDB database was performed using DALI. The ConSurf database was used to visualize conserved structural features of the Ssc-CdnE03. Structural alignments and generation of surface electrostatics with apo-OAS1 (PDB:4RWQ) and OAS1:dsRNA (PDB:4RWO) were performed using PyMOL.

### Generation of GFP-tagged Φ80α-vir

Wild-type Φ80α-vir was passaged on a liquid culture of *S. aureus* RN4220 harbouring a plasmid (pDVB434) encoding the *gfp* gene flanked by 500-nucleotide upstream and downstream homology arms corresponding to Φ80α *gp18* and *gp19*, respectively. To isolate individual plaques, the lysed culture supernatant spotted onto a lawn of RN4220 harbouring a type II-A Sau CRISPR–Cas targeting plasmid (pDVB435) in BHI soft agar for counter-selection against wild-type phage and enrichment of Φ80α-vir::GFP. The *gp18* and *gp19* genes were amplified by PCR and the *gfp* insertion was confirmed by Sanger sequencing.

### Time-lapse fluorescence microscopy

*S. aureus* cells harbouring Ssc-CBASS or lacking Cap15 were loaded onto microfluidic chambers using the CellASIC ONIX2 microfluidic system. After cells became trapped in the chamber, they were supplied with BHI medium with 5 mM CaCl_2_ and 10 μM propidium iodide under a constant flow of 5 μl h^−1^. After 1 h, GFP-tagged Φ80α-vir was flowed through the chambers for 1 h, before switching back to growth medium. Phase contrast images were captured at 1,000× magnification every 2 min using a Nikon Ti2e inverted microscope equipped with a Hamamatsu Orca-Fusion SCMOS camera and the temperature-controlled enclosure set to 37 °C. GFP was imaged using a GFP filter set and propidium iodide stain with DSRed filter set, both using an Excelitas Xylis LED Illuminator set to 2% power, with an exposure time of 300 ms. Images were aligned and processed using the NIS Elements software. Further downstream analysis of images was performed with Fiji v.2.3.0.

### Generation and isolation of escaper bacteriophages

Overnight cultures of *S. aureus* RN4220 were diluted 1:100 and outgrown at 37 °C with shaking for 1 h, infected with Φ80α-vir (MOI 1) for 20 min, and then treated with 1% EMS, a chemical mutagen. Cultures were allowed to lyse for 3 h before pelleting debris and sterile-filtering the supernatant to obtain an EMS-treated mutant phage library. One-hundred microlitres of RN4220 overnight cultures harbouring Ssc-CBASS were infected with a high-titre mutant phage library in BHI soft agar and then plated. After incubating at 37 °C overnight, individual phage plaques were picked from the top agar and resuspended in 50 μl of BHI liquid medium. Phage lysates were further purified over two rounds of passaging on RN4220 harbouring Ssc-CBASS.

### Whole-genome sequencing and analysis

Genomic DNA from high-titre phage stocks was extracted using a previously described method^41^. DNA was sheared to 300-bp fragments using an S220 Covaris Focused-Ultrasonicator (peak incident power: 140 W, duty factor: 10%, cycles per burst: 200, treatment time: 80 s, temperature 4 °C) in S-Series Holder microTUBEs (PN 500114). Library preparation was performed using the Illumina Nextera XT DNA Library Preparation Kit protocol (FC-131-1096). 12 pM of the library was loaded on an Illumina MiSeq instrument for paired-end sequencing (2 × 150 cycles). Bowtie2 via the Galaxy open-source interface^43^ was used to align sequencing reads to phage and host genomes. A custom Python script was used to convert the output SAM alignments into CSV files.

### Generation of recombinant ΦNM1γ6 *terS*^*S74F*^ mutants

Wild-type ΦNM1γ6 was passaged on *S. aureus* RN4220 harbouring Ssc-CBASS and pTerS or pTerS^S74F^ to enable recombination. The infected culture supernatant was spotted onto a lawn of RN4220 with Ssc-CBASS in BHI soft agar to isolate individual escaper plaques. The *terS* gene was amplified by PCR and the S74F mutation was confirmed by Sanger sequencing.

### Generation of recoded Φ80α-vir cabRNA mutants

Wild-type Φ80α-vir was passaged on a liquid culture of *S. aureus* RN4220 harbouring a plasmid (pDVB442, pDVB443, or pDVB460) encoding a mutant cabRNA sequence with silent transition mutations at the wobble positions of each codon and flanking homology arms. To isolate individual plaques, the lysed culture supernatant spotted onto a lawn of RN4220 harbouring a type II-A Sau CRISPR–Cas targeting plasmid (pDVB444) in BHI soft agar for counter-selection against wild-type phage and enrichment of the recoded mutants. The *terS* and *terL* genes were amplified by PCR and the recombined phage mutants was confirmed by Sanger sequencing.

### Northern blot analysis

RNA was extracted from *S. aureus* RN4220 cells, with or without infection by Φ80α-vir, according to the Direct-Zol RNA MiniPrep Plus Kit (R2072) manufacturer’s protocol. RNA samples were diluted in an equal volume of sample buffer (90 mM Tris-borate, 2 mM EDTA, pH 8.3, 8 M Urea, 10% sucrose, 0.05% bromophenol blue, and 0.05% xylene cyanol) and denatured by heating at 65 °C for 15 min, followed by chilling on ice. Denatured samples were separated by 12% polyacrylamide-8 M urea denaturing gel electrophoresis at 250 V in 0.5× TBE buffer (45 mM Tris, 45 mM borate, and 1 mM EDTA, pH 8.0). For blotting, separated RNA was transferred onto BrightStar-Plus positively charged nylon membrane (ThermoFisher, AM10100) by semidry electroblotting in molecular grade water at 300 mA for 60 min. After EDC crosslinking, the membrane was blocked in 6× SSC and 7% SDS in a 65 °C oven for 1 h. The membrane was then incubated overnight at 42 °C with PCR-generated double-stranded DNA probes against the cabRNA labelled with fluorescein. After washing with 0.1% SDS in 3× SSC 3 times for 10 min at 42 °C the blots were imaged using a Typhoon Trio Imager System for detection of fluorescein signal.

### Phylogenetic analysis of CD-NTase sequences

The CD-NTases from *S. schleiferi* are most similar to the CdnE subtype 03 (CdnE03) described by Whiteley et al.^8^. All CD-NTase enzymes were aligned using TCoffee Multiple Sequence Alignment tool (default parameters) and used to construct a phylogenetic tree with Geneious Prime using the neighbour-joining method and Jukes–Cantor genetic distance model with no outgroup.

### Statistical analysis

All statistical analyses were performed using GraphPad Prism v9.5.1. Error bars and number of replicates for each experiment are defined in the figure legends. Comparisons between groups for viral titre, gene expression, colony-forming units, and signalling intensity were analysed by unpaired parametric *t*-test, two-tailed with no corrections. Comparisons of signal intensities from phosphor screen images were quantified using Fiji v2.3.

### Reporting summary

Further information on research design is available in the Nature Portfolio Reporting Summary linked to this article.

## Online content

Any methods, additional references, Nature Portfolio reporting summaries, source data, extended data, supplementary information, acknowledgements, peer review information; details of author contributions and competing interests; and statements of data and code availability are available at 10.1038/s41586-023-06743-9.

### Supplementary information

Supplementary Discussion

Reporting Summary

Supplementary Figure 1 Raw images.

Supplementary Text Mass spectrometry analysis of the products of the Ssc-CdnE03 cyclase.

Supplementary Tables Supplementary Tables 1-5.

Supplementary Sequences Sequences and structures of the different RNAs described in this study.

## Data Availability

Source data are provided with this paper. Any additional data from this study are available from the lead contact upon request. The raw FASTQ files for the next-generation sequencing experiments can be found at the NCBI Sequence Read Archive (SRA) under BioProject PRJNA1016327. The complete genomes of ΦJ1, ΦJ2 and ΦJ4 are deposited in GenBank with accession numbers OR453397, OR453398 and OR453399, respectively. The crystal structures in Extended Data Fig. 4a–c are derived from Protein Data Bank 4RWQ and 4RWO.

## Code availability

Custom Python scripts used for data analysis are deposited at github.com/Marraffini-Lab/Banh_Roberts_etal_2023.
