# Author Correction: Bacterial cGAS senses a viral RNA to initiate immunity

**DOI:** 10.1038/s41586-023-06929-1

**Published:** 2023-12-05

**Authors:** Dalton V. Banh, Cameron G. Roberts, Adrian Morales-Amador, Brandon A. Berryhill, Waqas Chaudhry, Bruce R. Levin, Sean F. Brady, Luciano A. Marraffini

**Affiliations:** 1 Laboratory of Bacteriology, The Rockefeller University, New York, NY USA; 2 Weill Cornell/Rockefeller/Sloan Kettering Tri-Institutional MD-PhD Program, New York, NY USA; 3 Laboratory of Genetically Encoded Small Molecules, The Rockefeller University, New York, NY USA; 4 Department of Biology, Emory University, Atlanta, GA USA; 5 Howard Hughes Medical Institute, The Rockefeller University, New York, NY USA

**Keywords:** Bacteria, Bacteriophages

Correction to: *Nature* 10.1038/s41586-023-06743-9 Published online 15 November 2023

In the version of the article initially published, there were errors in the Abstract and Discussion. In the Abstract, the sentence now reading “Since mammalian oligoadenylate synthetases also bind viral double-stranded RNA…” previously read “As the mammalian cyclase OAS1 also binds viral double-stranded RNA…”. In the first paragraph of the Discussion, the sentence now reading “A similar observation has been reported for the human antiviral 2′,5′-oligoadenylate synthetase (OAS) enzymes OAS1 and OAS3…” previously read “A similar observation has been reported for the human OAS1 and OAS3 cyclases…”. At the beginning of the final paragraph of the Discussion, “Eukaryotic synthases” previously read “Eukaryotic cyclases”. These changes have been made to the HTML and PDF versions of the article.

