# Supplementary material for: Bacterial cGAS senses a viral RNA to initiate immunity
Source: Nature. 2023 Nov 15;623(7989):1001–8. doi: 10.1038/s41586-023-06743-9 (PMC10686824; doi:10.1038/s41586-023-06743-9)
Supplement: Supplementary file 1 — Supplementary Discussion [file 41586_2023_6743_MOESM1_ESM.docx]

**Supplementary Discussion**

*Importance of the cabRNA size and its hairpins for cyclase activation*

The positively charged surface of Ssc-CdnE03 (approximately 40 Å in length) should be able to only accommodate a dsRNA of approximately 20 base pairs. We tested the two sequences with the strongest probability of hairpin formation (#1 and #2) within the cabRNA and found that hairpin #1, but not #2, mediated substantial Ssc-CdnE03 activation *in vitro* (Extended Data Fig. 3e). We also reduced the size of the cabRNA *in vivo*, through the introduction of mutations in Φ80α-vir(cabRNA^100^), to 80 nucleotides (Fig. 5c). This sequence, which could not be mutated without altering the amino acid coding of the *terS/L* genes, includes hairpin #2. Given that our findings suggest that the formation of cabRNA secondary structures is facilitated during phage infection (Extended Data Fig. 3f), it is possible that hairpin #2 cannot adopt an activating form *in vitro*, but only during the viral lytic cycle. On the other hand, the RNA pulled down from infected staphylococci with Sha-CdnE01 included, in addition to the 400-nt cabRNA, an 80-nt species that contained the sequence of hairpin #1. Therefore, it remains incompletely understood the exact contributions of hairpin #1, hairpin #2, or any other region of the Φ80α-vir and ΦNM1γ6 cabRNAs to cyclase activation. A similar observation has been reported for the human OAS1 and OAS3 cyclases, which also require large dsRNA molecules for optimal activity ^1^.

*Different CBASS cyclases may have different mechanisms of activation*

CBASS cyclases that belong to the E03 families are widely distributed in different organisms (Extended Data Fig. 5a) and therefore we believe that the recognition of viral RNA for the activation of CBASS is a widespread mechanism across prokaryotes. However, there are many enzymes that seem to lack the basic patch we identified in Ssc-CdnE03 and Sha-CdnE01. This observation suggests that there are other modes of cyclase activation, both in staphylococci and in other CBASS types, that do not sense viral RNA. This idea is also supported by our findings that the staphylococcal phages ΦNM4γ4 and Φ12γ3 do not produce cabRNA and are not restricted by Ssc-CBASS. Effective immunity against these phages may be provided by either the above-mentioned CBASS cyclases that sense viral signals different than the cabRNA, or by some of the many diverse mechanisms of anti-phage defense that exist in bacteria ^2^.

*The function of the cabRNA in the viral lytic cycle*

In spite of its importance for the phage lytic cycle, the function of the cabRNA is unclear. One possibility is that, similarly to the prohead RNA of the *Bacillus* phage Φ29 ^3^, the cabRNA interacts with components of the phage capsid to participate in capsid biogenesis and/or viral DNA packaging. The Φ29 prohead RNA is a 174-nt RNA that is transcribed from an open reading frame of a viral protein of unknown function ^4^ that associates with the capsid vertex complex to participate in the packaging of the viral genome into empty capsids ^5^. The prohead RNA has extensive secondary structure and is believed to form an intermolecular pentameric ring ^6^. While the formation of such structure is certainly possible for the wild-type cabRNA of Φ80α-vir, it is less likely for the small cabRNAs produced by the Φ80α-vir(cabRNA^100^), Φ80α-vir(cabRNA^57^), ΦJ1, ΦJ2, and ΦJ4 phages. It is also not known how the cabRNA is produced during infection. Given that expression of a cabRNA in uninfected hosts generates mostly the unfolded version of this species (Extended Data Fig. 3f), which is pulled down by the Ssc-CdnE03 cyclase but fails to activate the CBASS response (Extended Data Fig. 3g), we speculate that phage infection is required for the folding of the cabRNA into its cyclase-activating form. In addition, since the S74F mutation in TerS, which does not change the cabRNA sequence, leads to the production of a longer form of the cabRNA (Fig. 4d) that can be reverted to the 400-nt form by the expression *in trans* of wild-type TerS (Fig. 4d), we conclude that this protein is required to determine the proper length of the cabRNA.

**References**

1 Wang, Y., Holleufer, A., Gad, H. H. & Hartmann, R. Length dependent activation of OAS proteins by dsRNA. *Cytokine* **126**, 154867.

2 Bernheim, A. & Sorek, R. The pan-immune system of bacteria: antiviral defence as a community resource. *Nat. Rev. Microbiol.* **18**, 113-119.

3 Hendrix, R. W. Bacteriophage DNA packaging: RNA gears in a DNA transport machine. *Cell* **94**, 147-150.

4 Guo, P. X., Bailey, S., Bodley, J. W. & Anderson, D. Characterization of the small RNA of the bacteriophage phi 29 DNA packaging machine. *Nucleic Acids Res.* **15**, 7081-7090.

5 Guo, P. X., Erickson, S. & Anderson, D. A small viral RNA is required for in vitro packaging of bacteriophage phi 29 DNA. *Science* **236**, 690-694.

6 Ding, F. *et al.* Structure and assembly of the essential RNA ring component of a viral DNA packaging motor. *Proc Natl Acad Sci U S A* **108**, 7357-7362.
