# Supplementary material for: Bacterial cGAS senses a viral RNA to initiate immunity
Source: Nature. 2023 Nov 15;623(7989):1001–8. doi: 10.1038/s41586-023-06743-9 (PMC10686824; doi:10.1038/s41586-023-06743-9)

Supplementary Figure 1

Original source images for data obtained by spotting phage on lawns of staphylococci

Box indicates location of cropped images in **Figure 1A**

A)

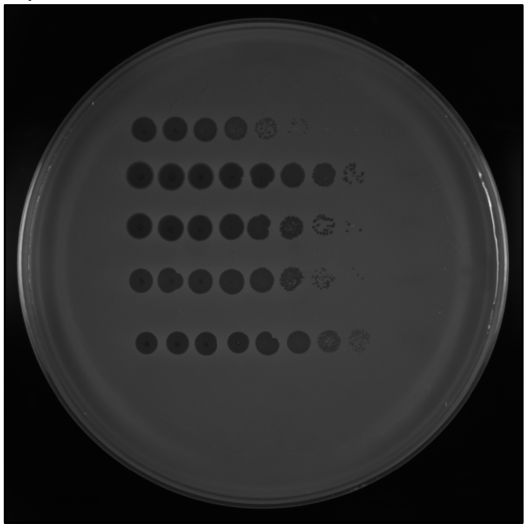

B)

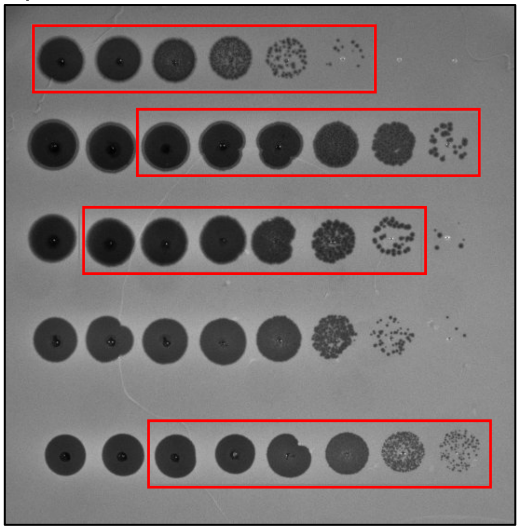

ϕNM1$\gamma 6$

$\phi 80\alpha - vir$

ϕNM4 $\gamma 4$

ϕSA2

ϕ12 $\gamma 3$

RN4220 + pSscCBASS dCap15

C)

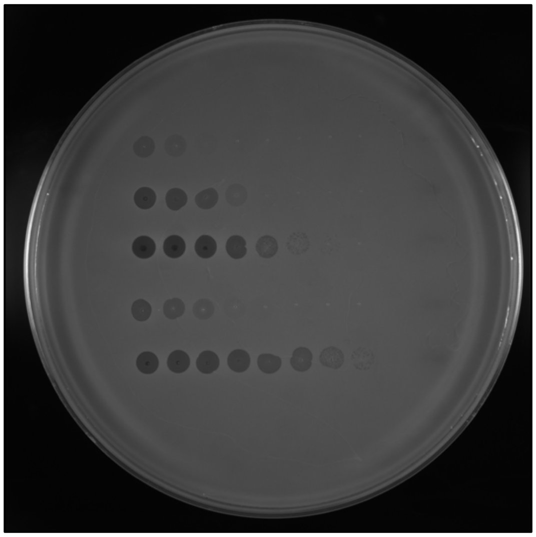

D)

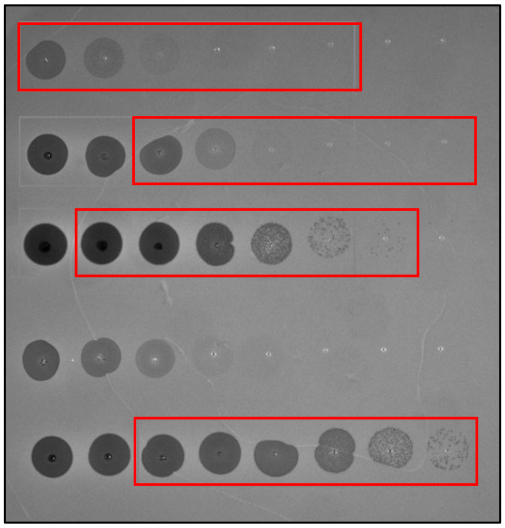

ϕNM1 $\gamma 6$

$\phi 80\alpha - vir$

ϕNM4 $\gamma 4$

ϕSA2

ϕ12 $\gamma 3$

RN4220 + pSscCBASS

Original source images for data obtained by TLC separation of cyclic nucleotides followed by phosphor screen imaging

Box indicates location of cropped images in **Figure 1B**

E)

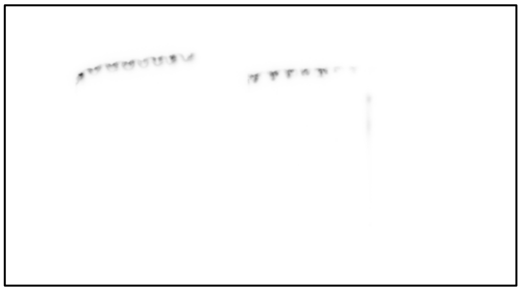

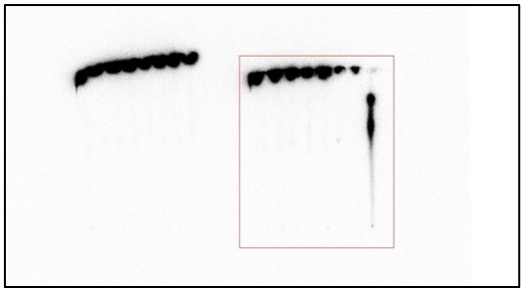

phosphor screen α-^{32}P NTPs

imageJ auto contrast applied to image on the right

F)

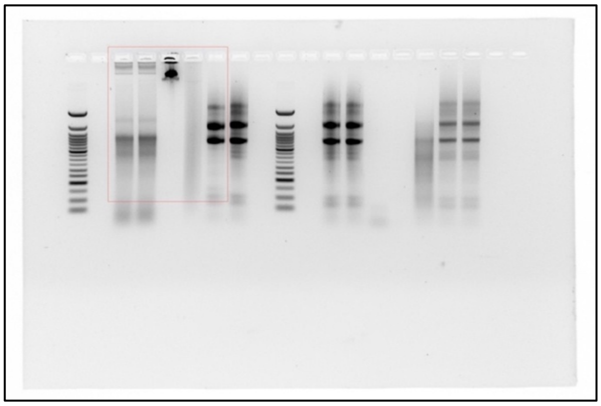

stained with ethidium bromide

Ladder: new England biolabs 50 bp DNA ladder

G)

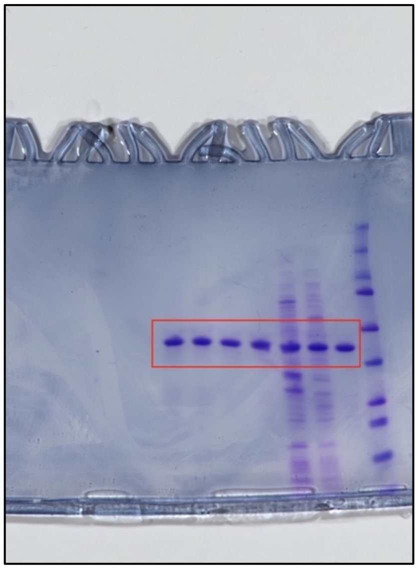

stained with Coomassie

Ladder: Bio-rad Precision Plus Protein Dual Color Standard

Original source images for data obtained by electrophoretic separation

Box indicates location of cropped images in **Figure 1C**

H)

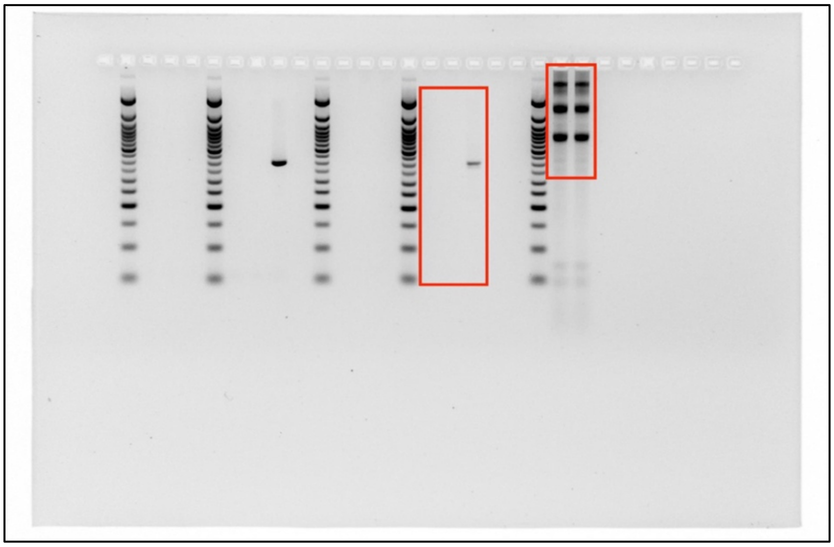

stained with ethidium bromide
Ladder: new England biolabs 50 bp DNA ladder

I)

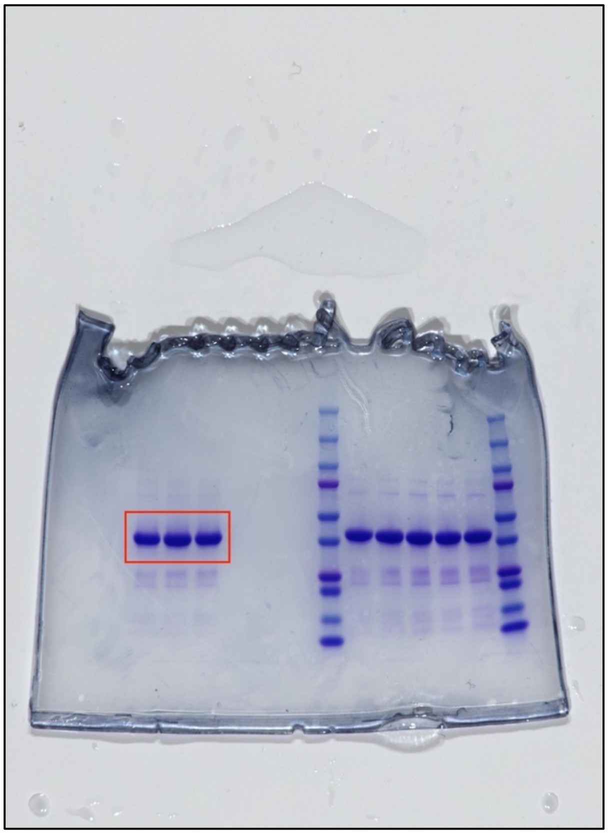

stained with Coomassie blue
Ladder: Bio-rad Precision Plus Protein Dual Color Standard

Original source images for data obtained by electrophoretic separation

Box indicates location of cropped images in **Figure 1D**

J)

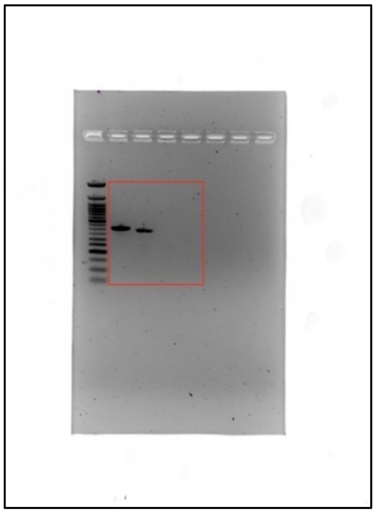

stained with ethidium bromide
Ladder: new England biolabs 50 bp DNA ladder

K)

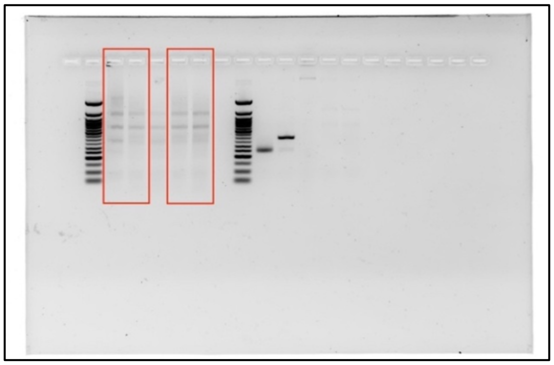

stained with ethidium bromide
Ladder: new England biolabs 50 bp DNA ladder

L)

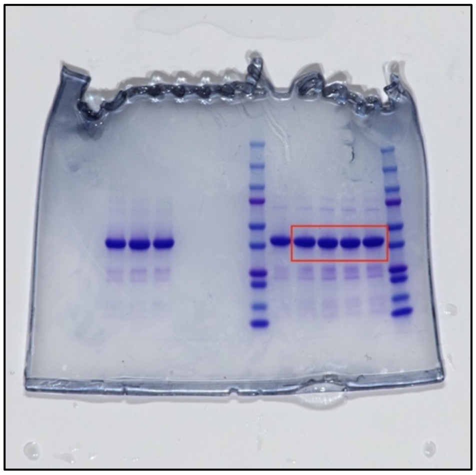

stained with Coomassie blue
Ladder: Bio-rad Precision Plus Protein Dual Color Standard

Original source images for data obtained by electrophoretic separation & data obtained by TLC separation of cyclic nucleotides followed by phosphor screen imaging

Box indicates location of cropped images in **Figure 1F**

M)

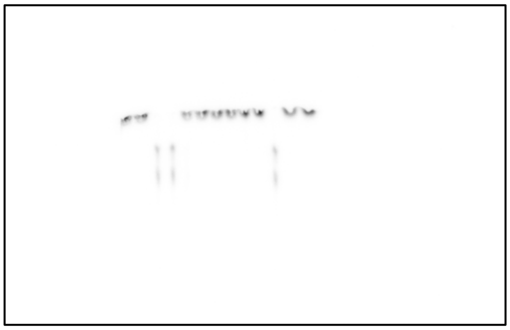

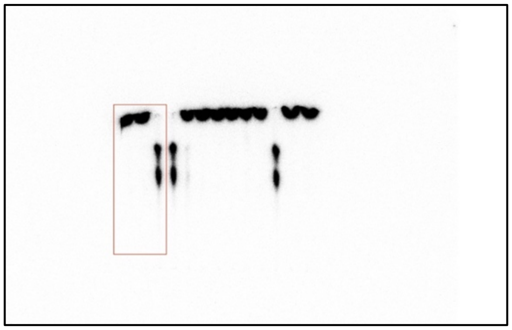

phosphor screen α-^{32}P NTPs

imageJ auto contrast applied to image on the right

N)

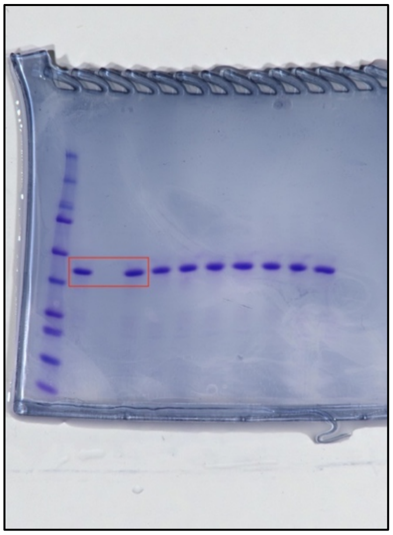

stained with Coomassie blue
Ladder: Bio-rad Precision Plus Protein Dual Color Standard

O)

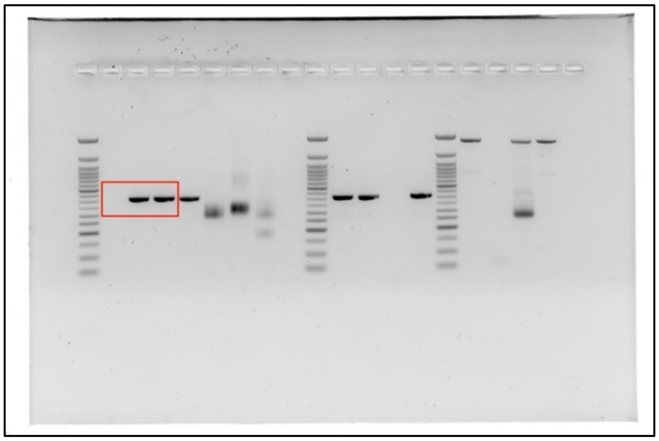

stained with ethidium bromide, Ladder: new England biolabs 50 bp DNA ladder

Original source images for data obtained by electrophoretic separation

Box indicates location of cropped images in **Figure 2A**

P)

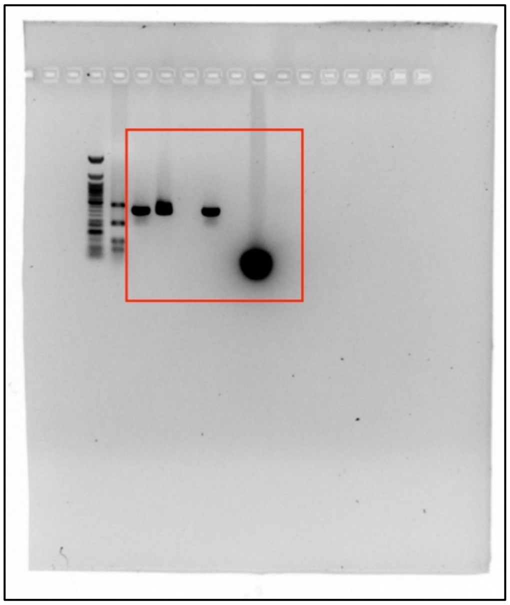

stained with ethidium bromide
Ladder: new England biolabs 50 bp DNA ladder and dsRNA ladder

Original source images for data obtained by electrophoretic separation & data obtained by TLC separation of cyclic nucleotides followed by phosphor screen imaging

Box indicates location of cropped images in **Figure 2B**

Q)

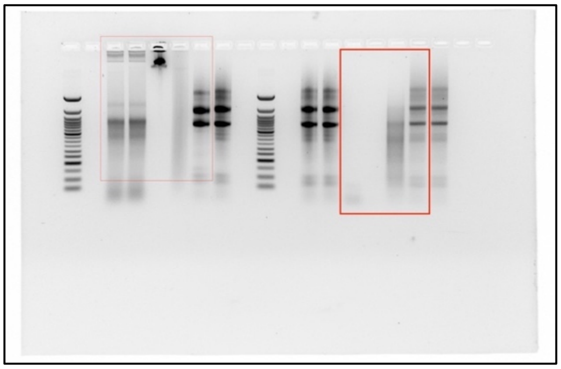

stained with ethidium bromide, Ladder: new England biolabs 50 bp DNA ladder

R)

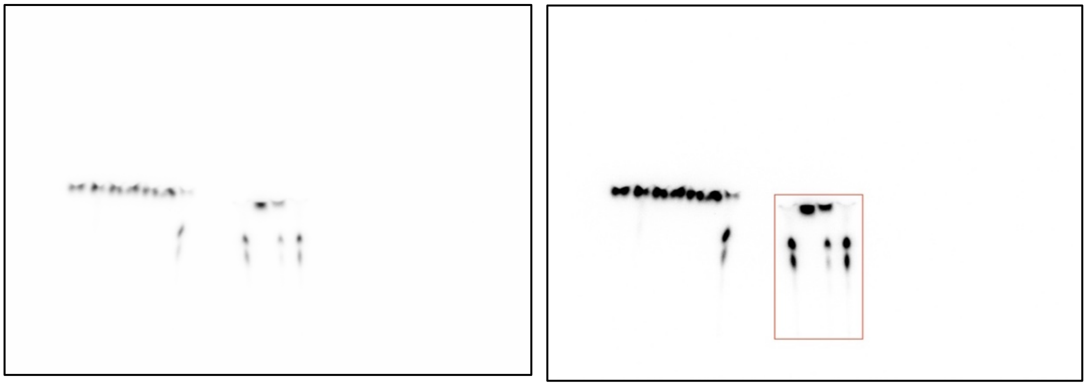

phosphor screen α-^{32}P NTPs

imageJ auto contrast applied to image on the right

Original source images for data obtained by electrophoretic separation

Box indicates location of cropped images in **Figure 2C**

S)

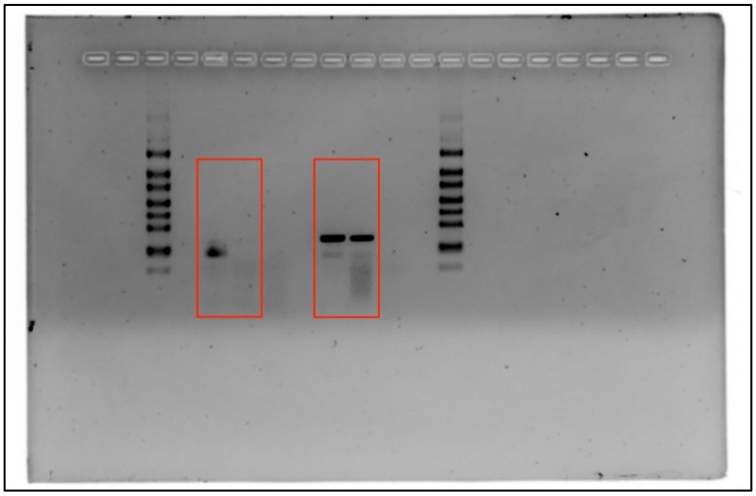

stained with ethidium bromide
Ladder: new England biolabs 50 bp DNA ladder

Original source images for data obtained by electrophoretic separation & data obtained by TLC separation of cyclic nucleotides followed by phosphor screen imaging

Box indicates location of cropped images in **Figure 2D**

T)

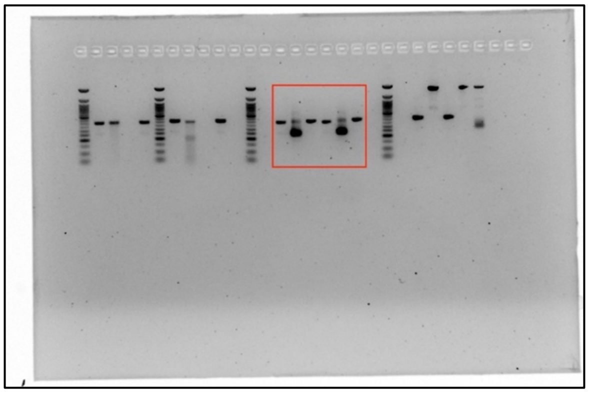

stained with ethidium bromide, Ladder: new England biolabs 50 bp DNA ladder

U)

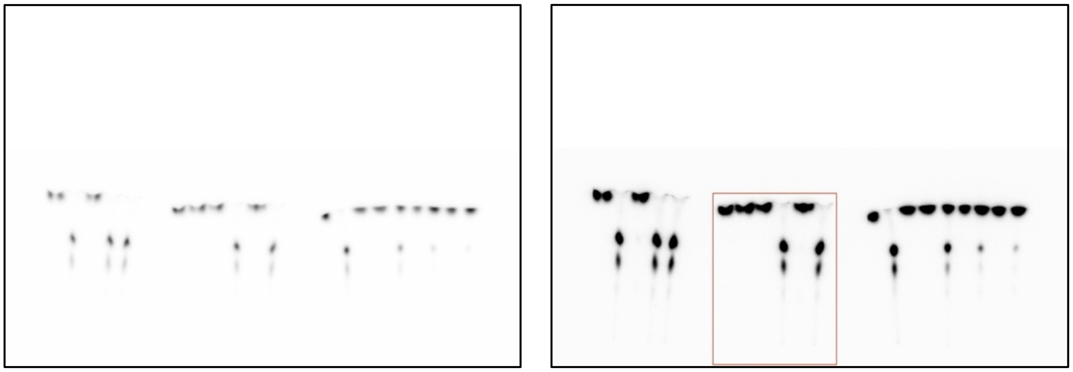

phosphor screen α-^{32}P NTPs

imageJ auto contrast applied to image on the right

Original source images for data obtained by TLC separation of cyclic nucleotides followed by phosphor screen imaging

Box indicates location of cropped images in **Figure 3B**

V)

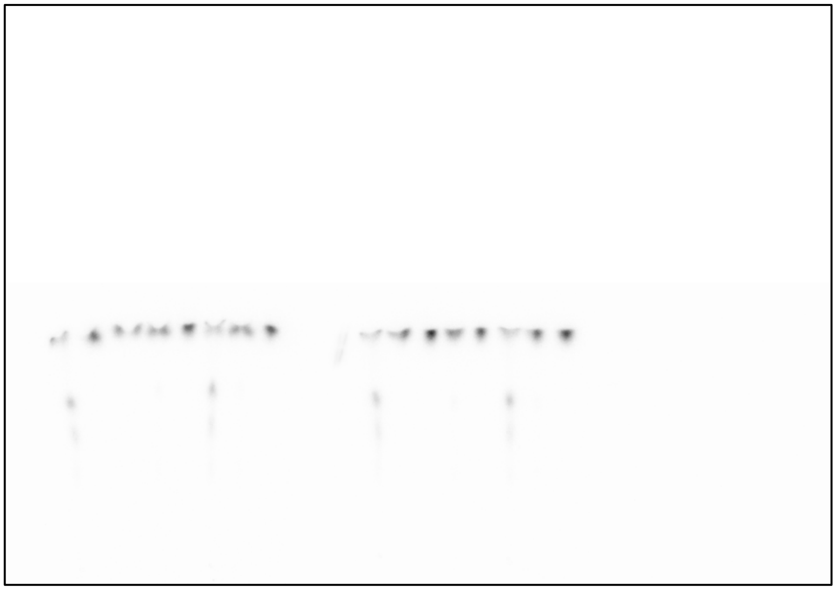

phosphor screen α-^{32}P NTPs

imageJ auto contrast applied to image on the bottom

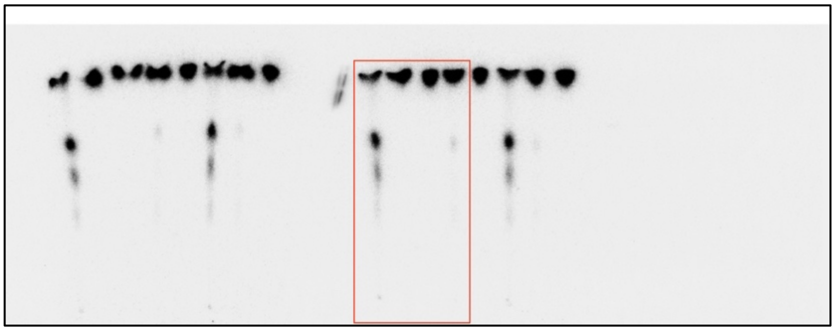

Original source images for data obtained by electrophoretic mobility assay

Box indicates location of cropped images in **Figure 3C**

W)

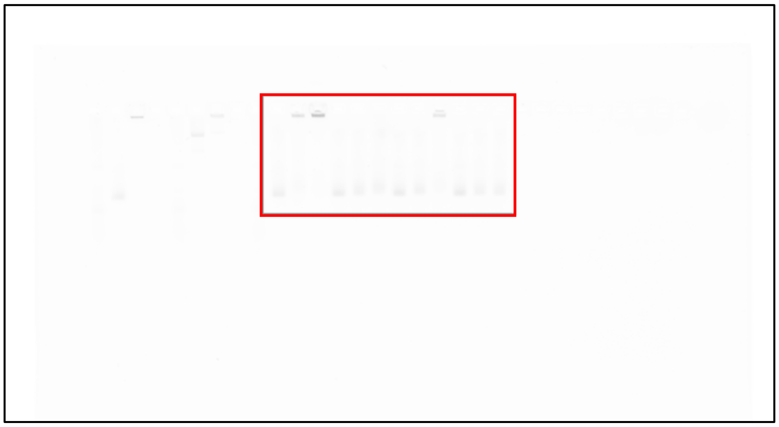

stained with ethidium bromide, Ladder: new England biolabs 50 bp DNA ladder

Original source images for data obtained by spotting phage on lawns of staphylococci

Box indicates location of cropped images in **Figure S5C**

X)

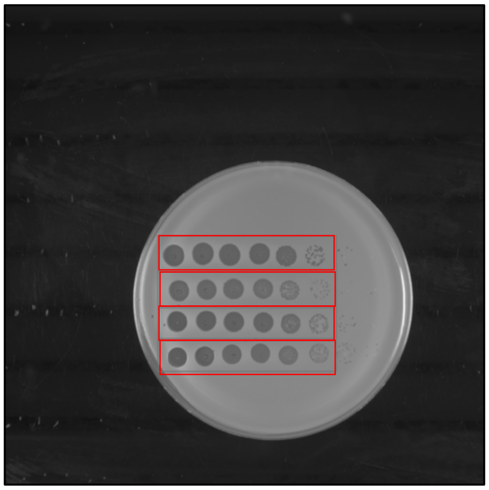

RN4220 + empty vector

Y)

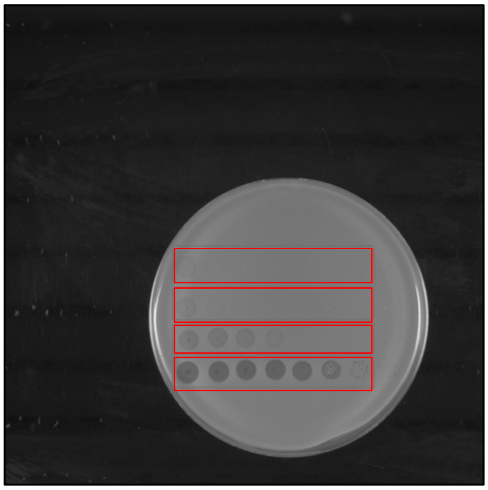

ϕ80α

ϕNM1

ϕNM4

ϕ12

RN4220 + pShaCBASS

Original source images for data obtained by electrophoretic separation

Box indicates location of cropped images in **Figure S5D**

Z)

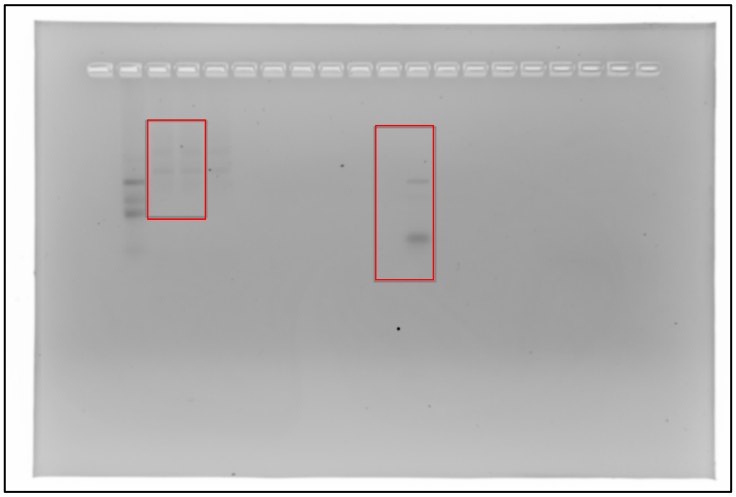

stained with ethidium bromide
Ladder: new England biolabs dsRNA ladder

Original source images for data obtained by spotting phage on lawns of staphylococci

Box indicates location of cropped images in **Figure 4B**

AA) **AB)** **AC)**

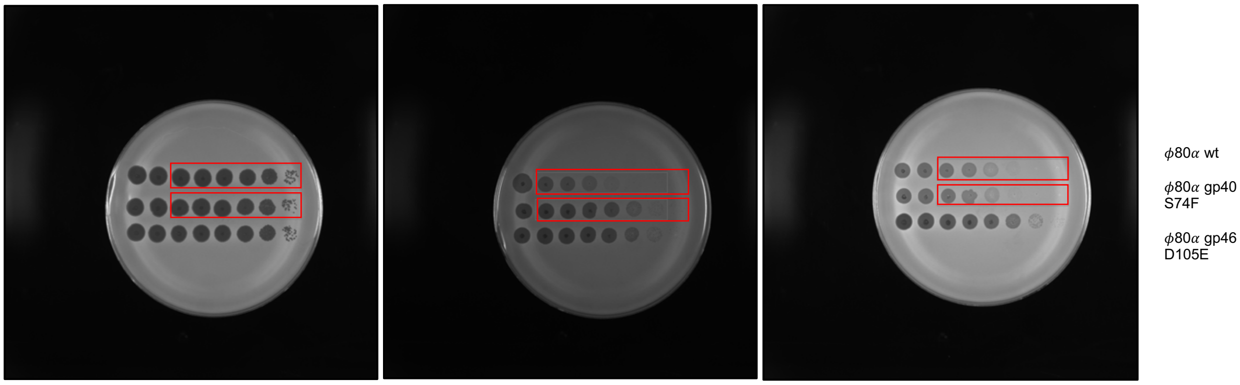

RN4220 + empty vector RN4220 + pCBASS RN4220 + pCBASS + pTerS

Original source images for data obtained by TLC separation of cyclic nucleotides followed by phosphor screen imaging & for data obtained by electrophoretic separation

Box indicates location of cropped images in **Figure 4C**

AD)

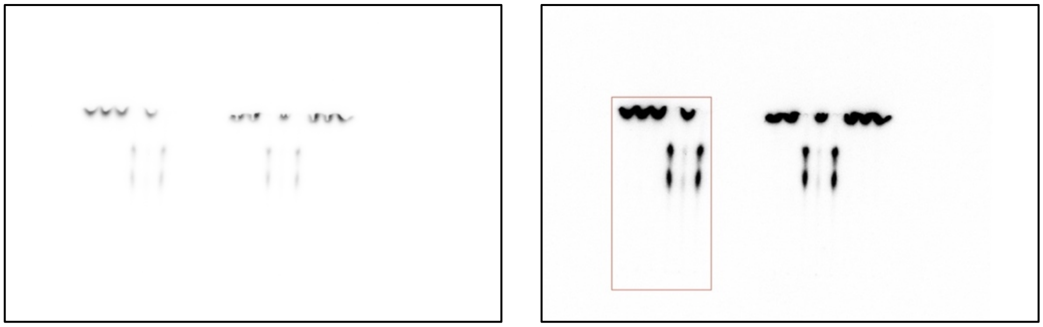

phosphor screen α-^{32}P NTPs

imageJ auto contrast applied to image on the right

AE)

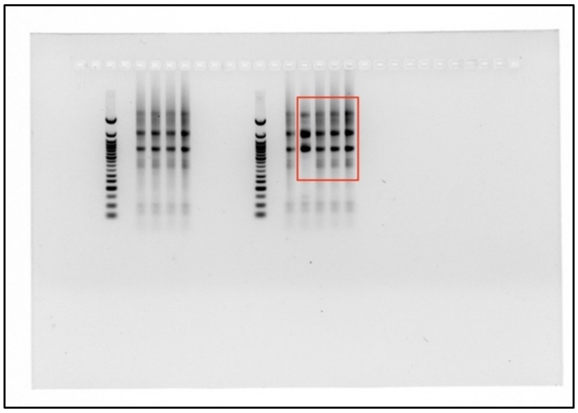

stained with ethidium bromide, Ladder: new England biolabs 50 bp DNA ladder

AF)

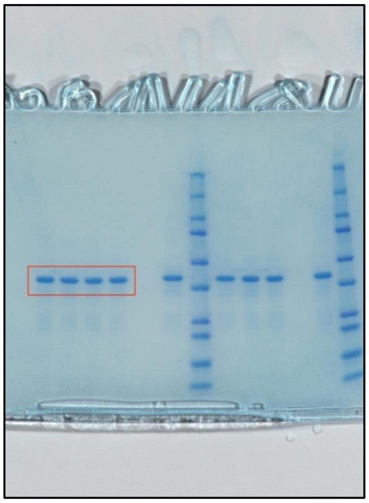

stained with Coomassie blue
Ladder: Bio-rad Precision Plus Protein Dual Color Standard

Original source images for data obtained by electrophoretic separation

Box indicates location of cropped images in **Figure 4D**

AG)

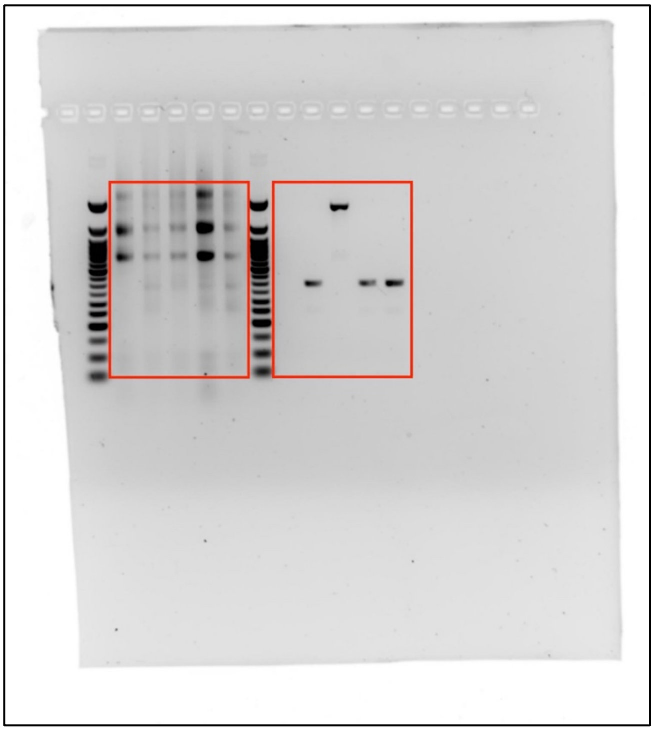

stained with ethidium bromide
Ladder: new England biolabs 50 bp DNA ladder

Original source images for data obtained by TLC separation of cyclic nucleotides followed by phosphor screen imaging & for data obtained by electrophoretic separation

Box indicates location of cropped images in **Figure 4E**

AH)

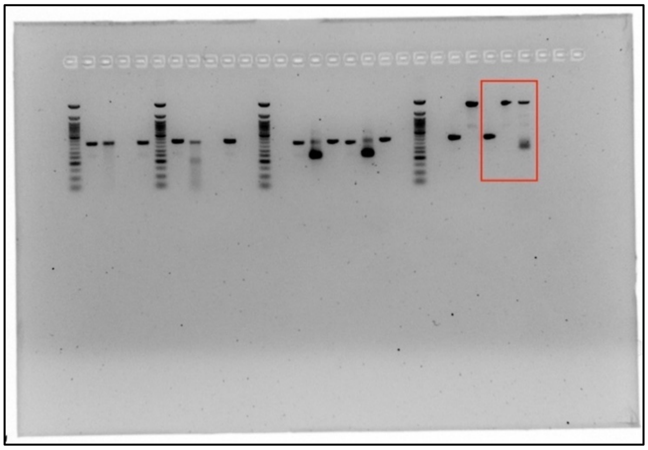

stained with ethidium bromide
Ladder: new England biolabs 50 bp DNA ladder

AI)

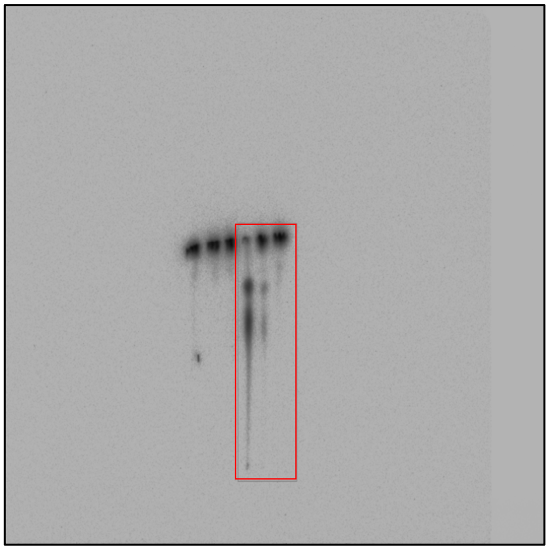

phosphor screen α-^{32}P NTPs

Original source images for data obtained by electrophoretic separation

Box indicates location of cropped images in **Figure 5C AJ)**

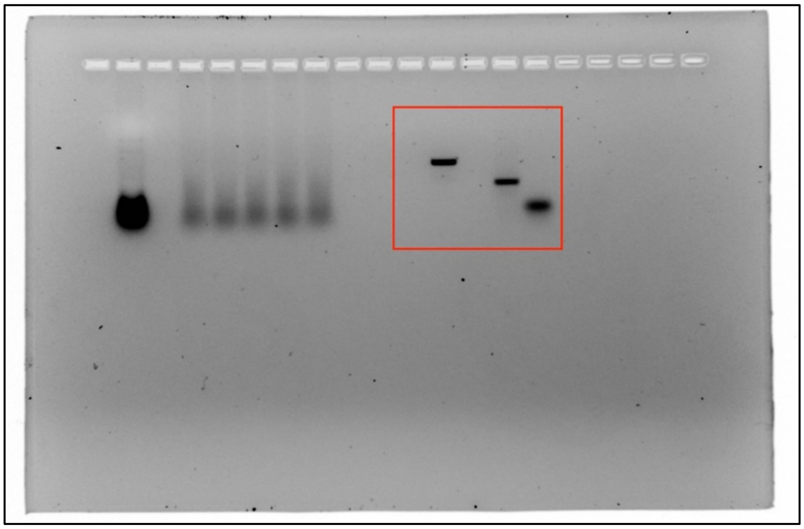

stained with ethidium bromide
Ladder: new England biolabs low range dsRNA ladder

Original source images for data obtained by TLC separation of cyclic nucleotides followed by phosphor screen imaging

Box indicates location of cropped images in **Figure 5D**

AK)

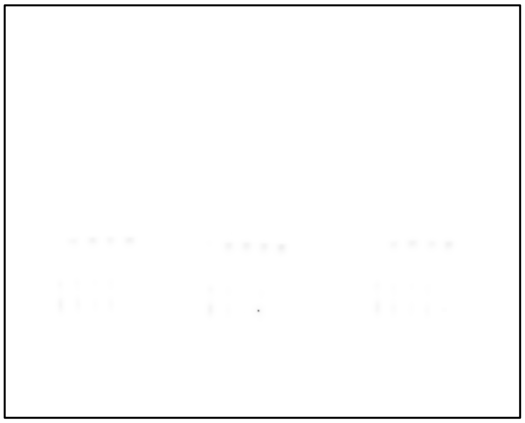

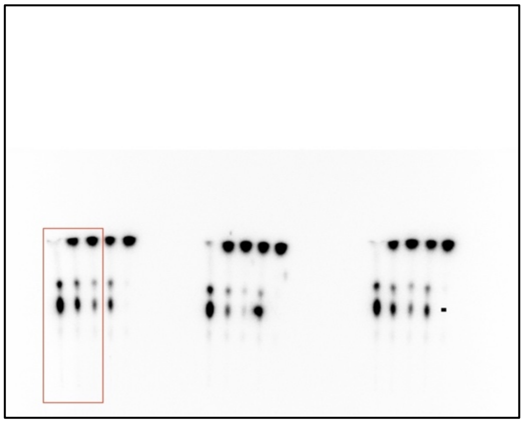

phosphor screen α-^{32}P NTPs

imageJ auto contrast applied to image on the right

Original source images for data obtained by spotting phage on lawns of staphylococci

Box indicates location of cropped images in **Figure 6A**

AL)

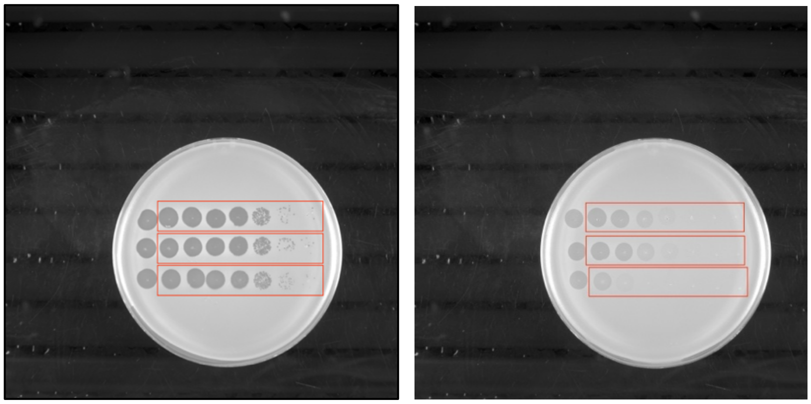

RN4220 + pSscCBASS dCap15 RN4220 + pSscCBASS

Original source images for data obtained by electrophoretic separation

Box indicates location of cropped images in **Figure 6B**

AM)

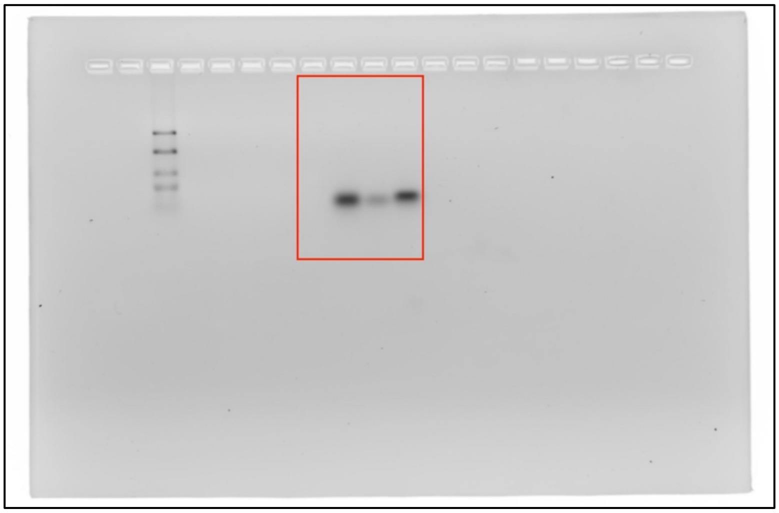

stained with ethidium bromide
Ladder: new England biolabs low range dsRNA ladder

Original source images for data obtained by TLC separation of cyclic nucleotides followed by phosphor screen imaging

Box indicates location of cropped images in **Figure 6C**

AN)

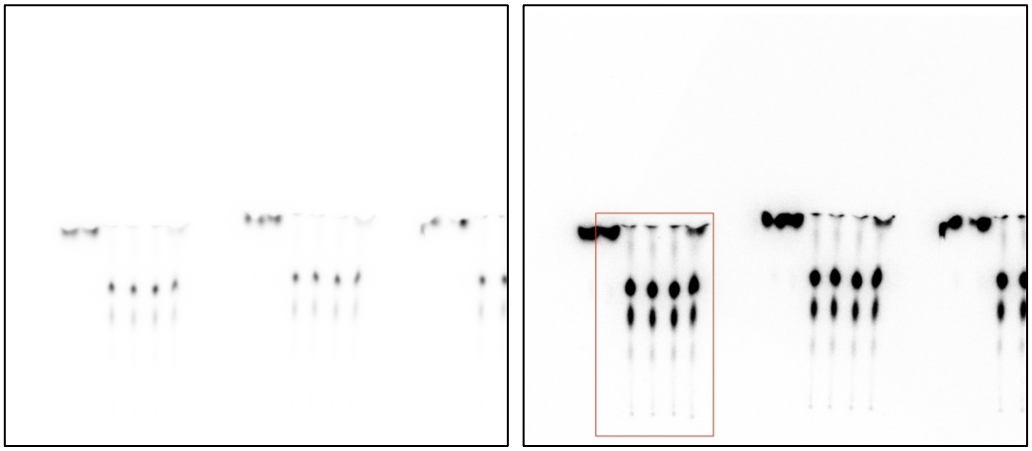

phosphor screen
α-^{32}P NTPs

imageJ auto contrast applied to image on the right

Original source images for data obtained by TLC separation of cyclic nucleotides followed by phosphor screen imaging

Box indicates location of cropped images in **Figure 6D**

AO)

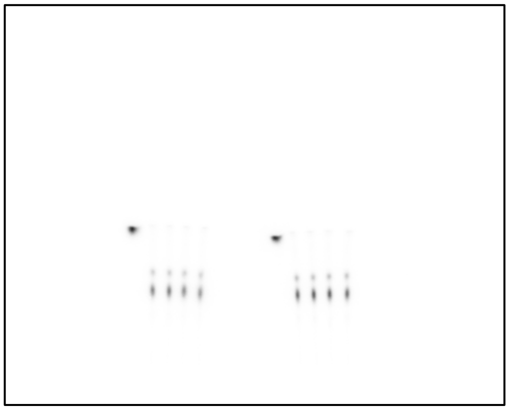

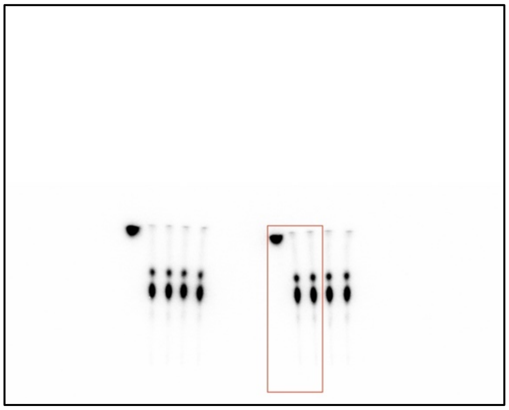

phosphor screen
α-^{32}P NTPs

imageJ auto contrast applied to image on the right

Original source images for data obtained by spotting phage on lawns of staphylococci

Box indicates location of cropped images in **Figure S1B**

AP)

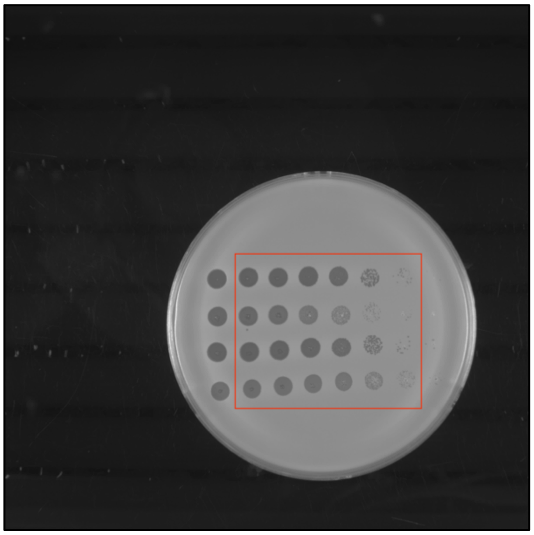

RN4220::SscCdnE03

AQ)

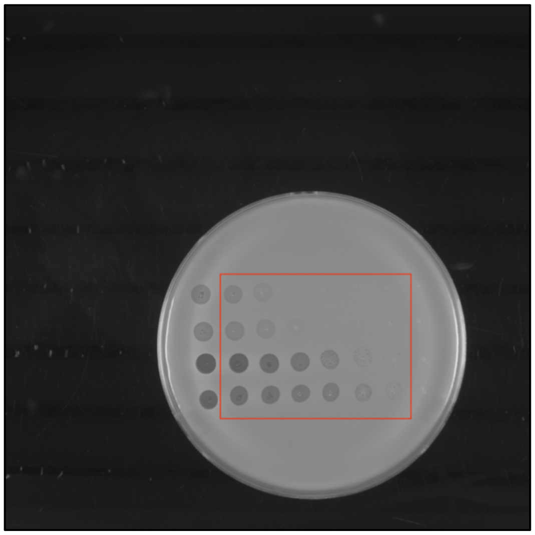

ϕNM1

$\phi 80\alpha$

ϕNM4

ϕ12

RN4220::CBASS

Original source images for data obtained by fluorescence confocal microscopy of staphylococci harboring Ssc-CBASS

Box indicates location of cropped images in **Figure S1K**

AR)

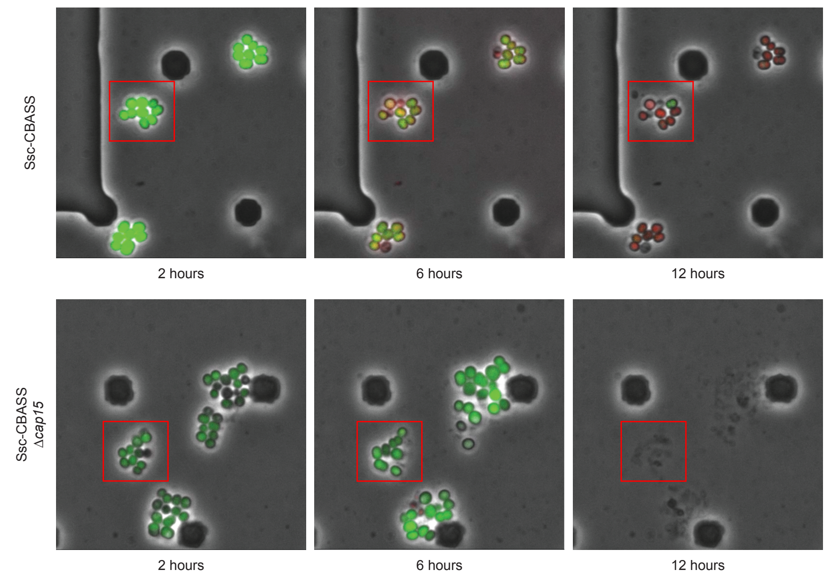

GFP channel = phage replication

RFP channel = propidium iodide staining, representing membrane disruption

Original source images for data obtained by TLC separation of cyclic nucleotides followed by phosphor screen imaging

Box indicates location of cropped images in **Figure S2D**

AS)

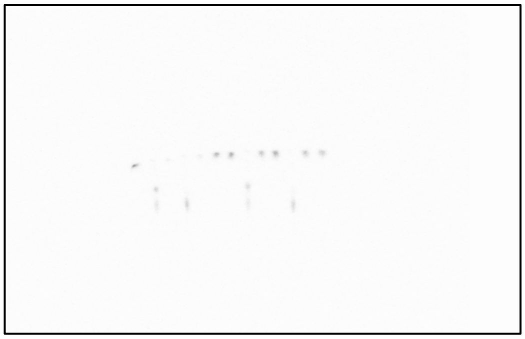

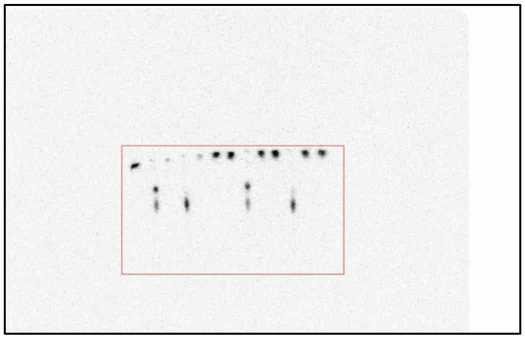

phosphor screen α-^{32}P NTPs

imageJ auto contrast applied to image on the right

Original source images for data obtained by TLC separation of cyclic nucleotides followed by phosphor screen imaging

Box indicates location of cropped images in **Figure S2G**

AT)

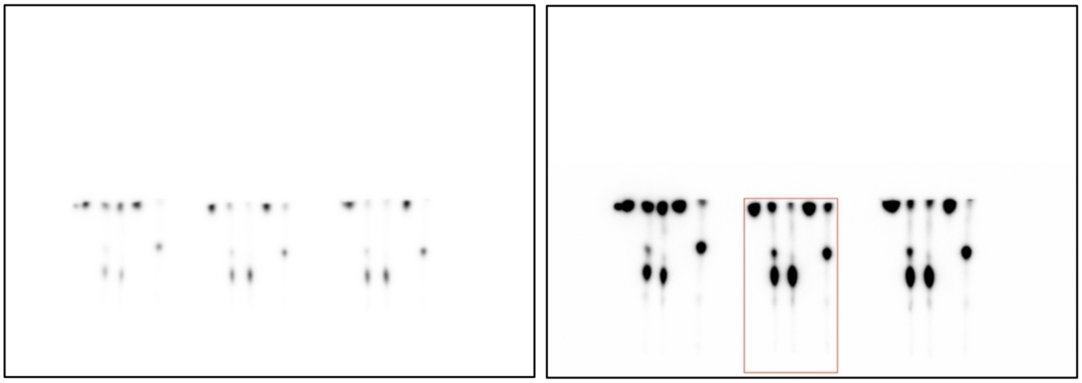

phosphor screen α-^{32}P NTPs

imageJ auto contrast applied to image on the right

Original source images for data obtained by electrophoretic separation of RNA followed by northern blot

Box indicates location of cropped images in **Figure S2I**

AU)

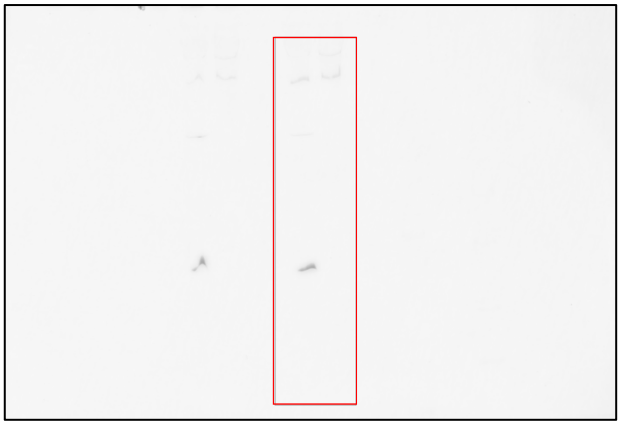

northern blot of one gel/membrane probed with 1 3’FAC dsDNA probe visualized in one fluorescence channel

Ladder Jena bioscience fluorescent low range DNA ladder

Original source images for data obtained by TLC separation of cyclic nucleotides followed by phosphor screen imaging & for data obtained by electrophoretic separation

Box indicates location of cropped images in **Figure S3B**

AV)

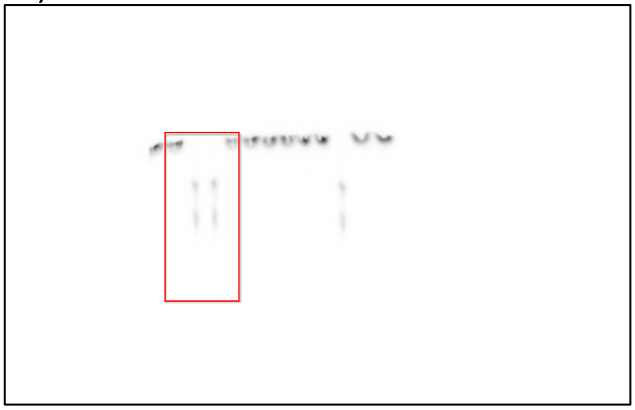

phosphor screen α-^{32}P NTPs

AW)

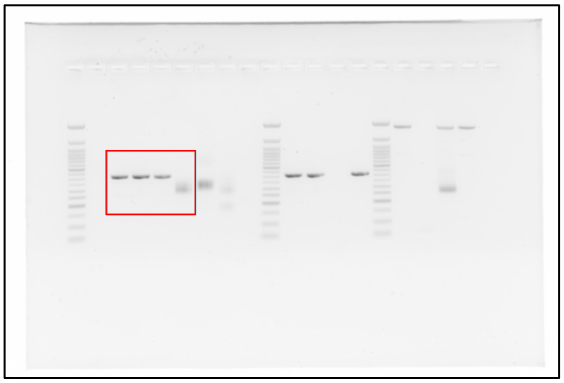

stained with ethidium bromide
Ladder: new England biolabs 50 bp DNA ladder

AX)

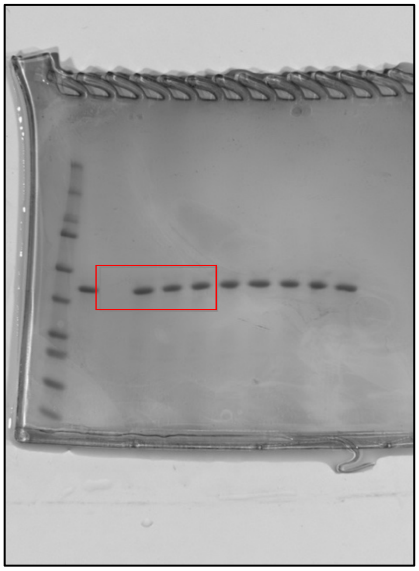

stained with Coomassie blue
Ladder: Bio-rad Precision Plus Protein Dual Color Standard

Original source images for data obtained by TLC separation of cyclic nucleotides followed by phosphor screen imaging

Box indicates location of cropped images in **Figure S3C**

AY)

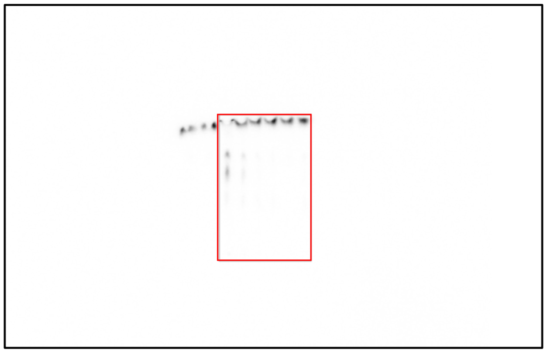

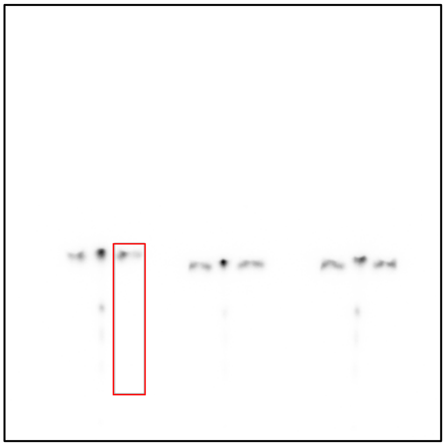

phosphor screen α-^{32}P NTPs

Original source images for data for data obtained by electrophoretic separation

Box indicates location of cropped images in **Figure S3F**

AZ)

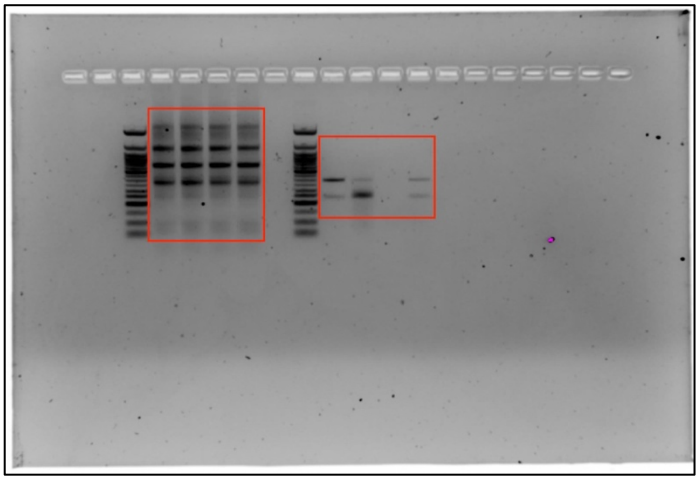

stained with ethidium bromide
Ladder: new England biolabs 50 bp DNA ladder

Original source images for data obtained by electrophoretic separation

Box indicates location of cropped images in **Figure S4G**

BA)

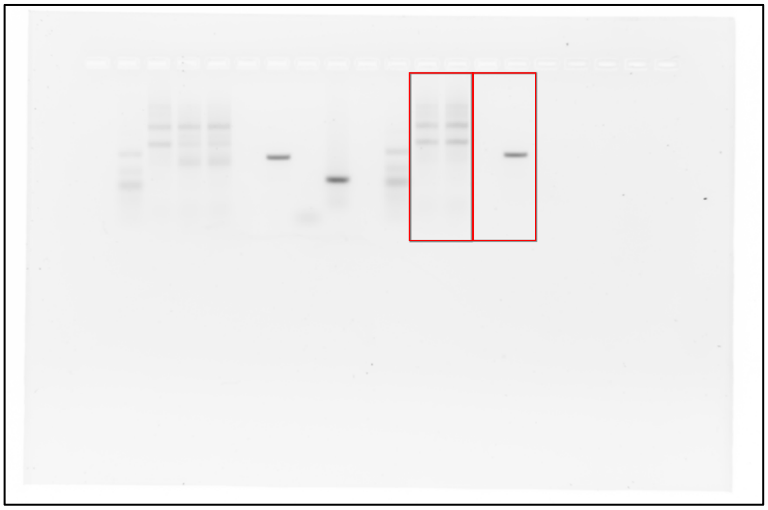

stained with ethidium bromide
Ladder: new England biolabs 50 bp DNA ladder

Original source images for data obtained by spotting phage on lawns of staphylococci

Box indicates location of cropped images in **Figure S6A**

BB)

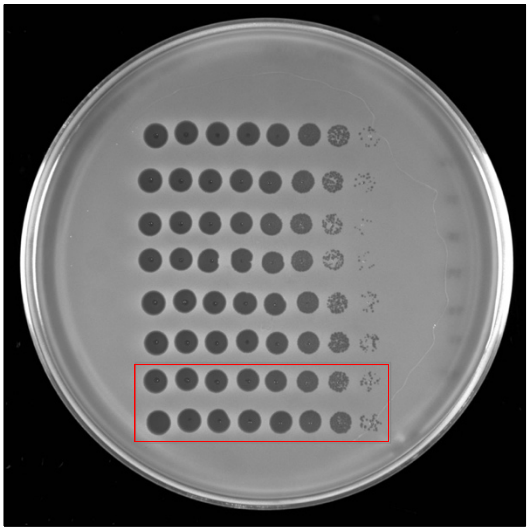

RN4220

BC)

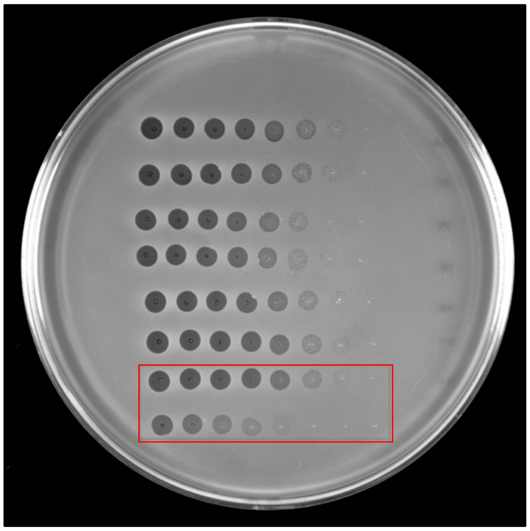

Replicates EMS treated phage libraries

1

2

3

4

5

6

7

wild-type

RN4220 + pSscCBASS

Original source images for data obtained by spotting phage on lawns of staphylococci

Box indicates location of cropped images in **Figure S6D**

BD) **BE)** **BF)**

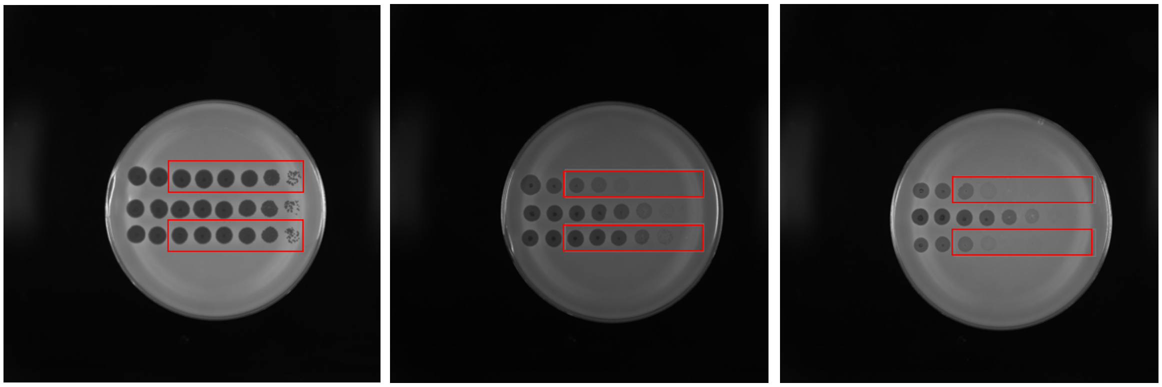

$\phi80\alpha$ wt

$\phi80\alpha$ gp40 S74F

$\phi80\alpha$ gp46 D105E

RN4220 + empty vector RN4220 + pCBASS RN4220 + pCBASS + pGp46

Original source images for data obtained by TLC separation of cyclic nucleotides followed by phosphor screen imaging

Box indicates location of cropped images in **Figure S6E**

BG)

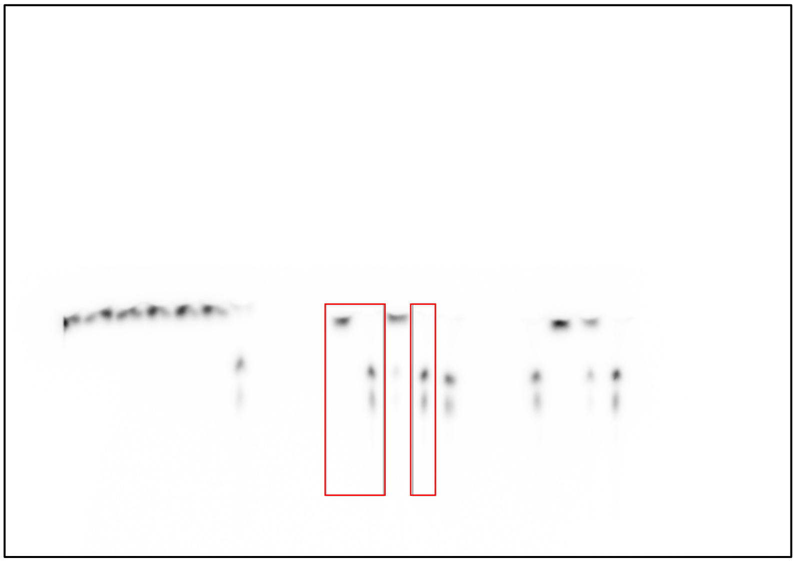

phosphor screen α-^{32}P NTPs

Original source images for data for data obtained by electrophoretic separation

Box indicates location of cropped images in **Figure S7A**

BH)

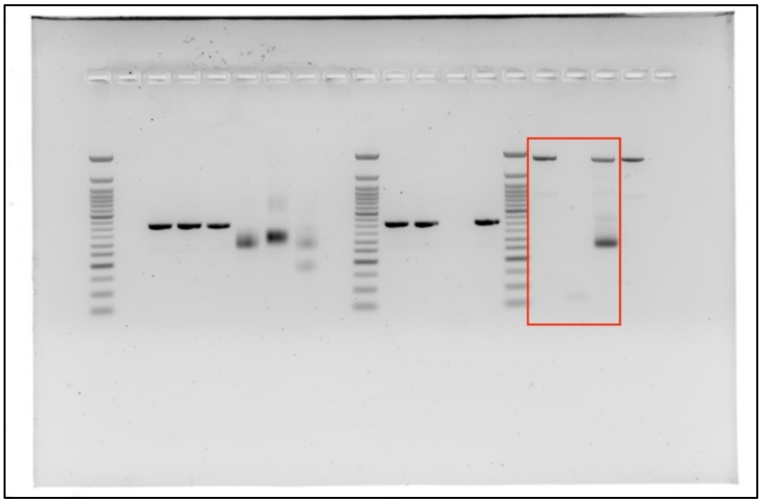

stained with ethidium bromide
Ladder: new England biolabs 50 bp DNA ladder

Original source images for data for data obtained by electrophoretic mobility assay

Box indicates location of cropped images in **Figure S7B**

BI)

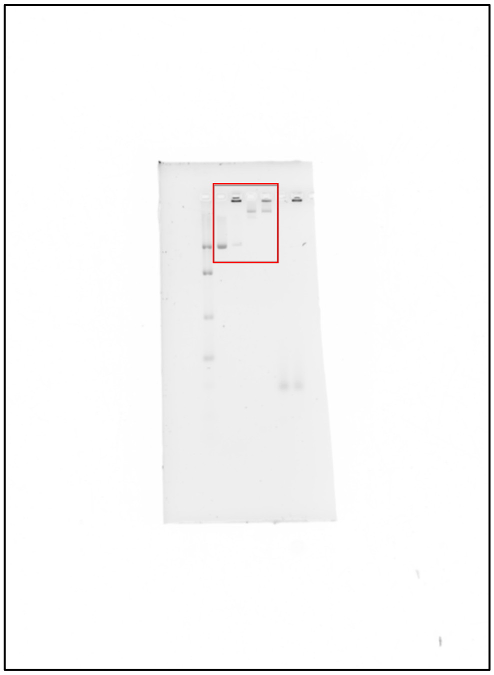

stained with ethidium bromide
Ladder: new England biolabs dsRNA ladder

Original source images for data obtained by TLC separation of cyclic nucleotides followed by phosphor screen imaging

Box indicates location of cropped images in **Figure S7D**

BJ)

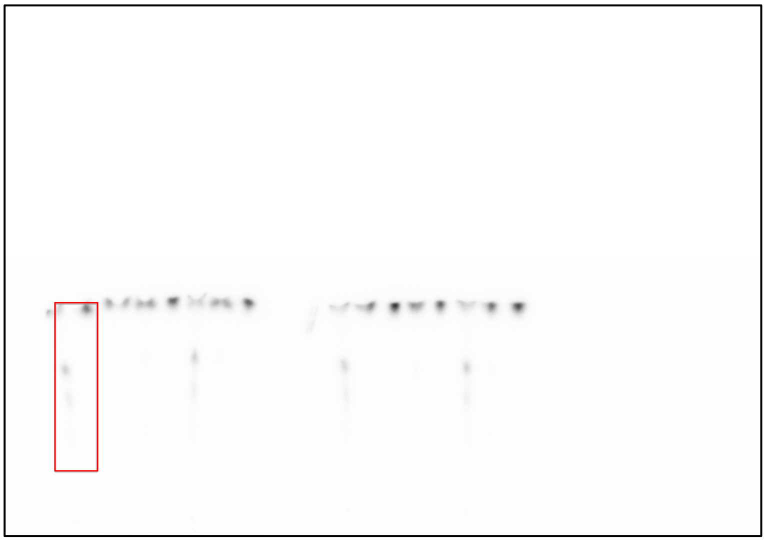

phosphor screen α-^{32}P NTPs

Original source images for data obtained by spotting phage on lawns of staphylococci

Box indicates location of cropped images in **Figure S7E**

BK) **BL)** **BM)**

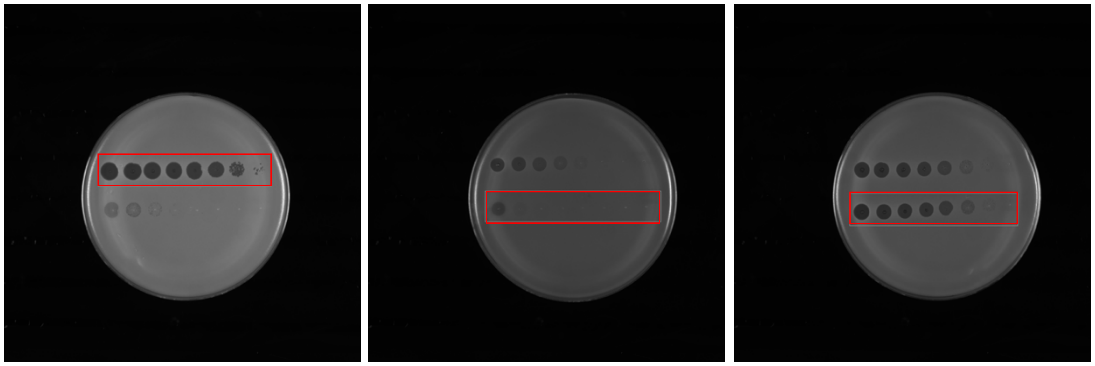

ϕNM1 propagated strains listed below

Spotted serial dilutions on the same strain

RN4220:Ssc-CdnE03 pTerS^{S74F} RN4220:Ssc-CBASS pTerS RN4220:Ssc-CBASS pTerS^{S74F}

Original source images for data obtained by spotting phage on lawns of staphylococci

Box indicates location of cropped images in **Figure S8B**

BN)

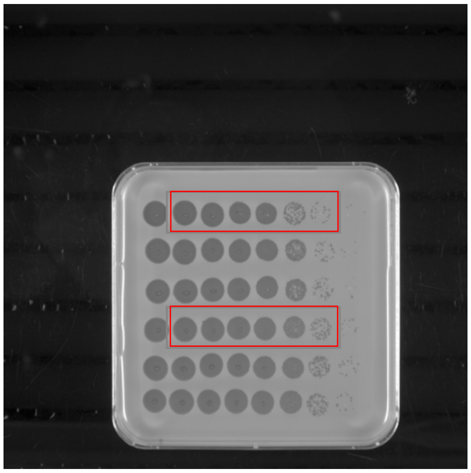

RN4220::SscCdnE03

BO)

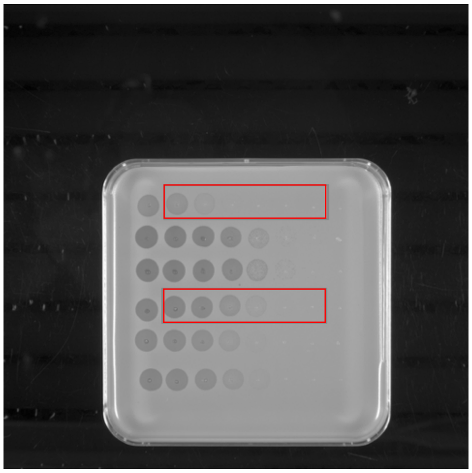

RN4220::CBASS

Original source images for data obtained by TLC separation of cyclic nucleotides followed by phosphor screen imaging

Box indicates location of cropped images in **Figure S8C**

BP)

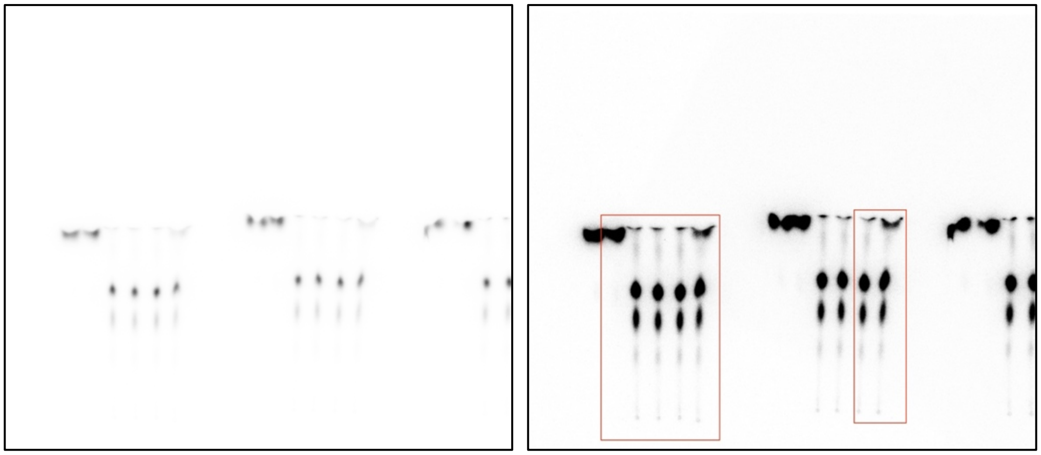

phosphor screen α-^{32}P NTPs

imageJ auto contrast applied to image on the right

BQ)

Reference for DNA/RNA gel ladders

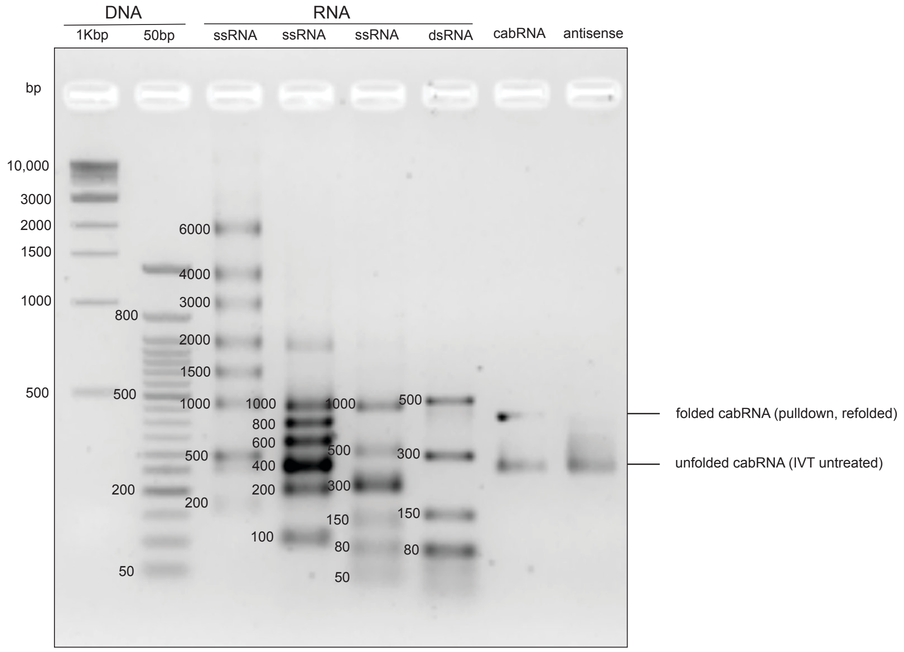

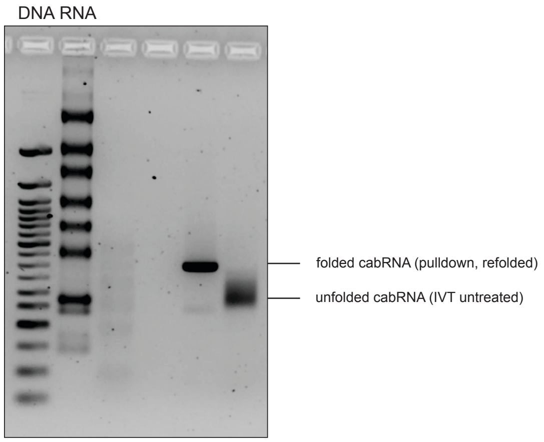

Supplement: Supplementary file 3 — Raw images. [file 41586_2023_6743_MOESM3_ESM.pdf]
