# Supplementary material for: Bacterial cGAS senses a viral RNA to initiate immunity
Source: Nature. 2023 Nov 15;623(7989):1001–8. doi: 10.1038/s41586-023-06743-9 (PMC10686824; doi:10.1038/s41586-023-06743-9)

**Supplementary Text.**

**High Resolution Mass Spectrometry Analysis of Ssc-CdnE03 reaction products.**

The Full HRMS of the products of the Ssc-CdnE03 cyclase shows that this peak corresponds to a compound identified from its mono and double charged ions in the positive and negative ionization modes, that allow the molecular formula C_15_H_19_N_5_O_13_P_2_ to be predicted, consistent with the molecular formula of cGAMP.

MS^2^ experiments were run in both ionization modes, however, the negative shows more diagnostic fragments and a consistent fragmentation pattern throughout the set of evaluated ions. The MS^2^ spectra of both cGAMP isomers show identical fragmentation to the Ssc-CdnE03 product, being particularly relevant the presence of fragment ions from cleavages a (a1, a2), b (b1, b2) and c (c1, c2) that allow identifying this product as a dimeric nucleotide constituted by GMP and AMP units. Based on these results we conclude that the Ssc-CdnE03 product is a cGAMP isomer or a mixture of them.

**NEGATIVE ION MODE ACQUISITION**

**Characterization of standard 3´,3´-cGAMP:**

- Full HRMS (-ESI):

| Ion | Formula | *m/z* obs. | *m/z* theo. | RDB | ∆ppm |
| --- | --- | --- | --- | --- | --- |
| [M-H]^-^ | C_20_H_23_N_10_O_13_P_2_^-^ | 673.0930 | 673.0926 | 13.5 | 0.59 |
| [M-2H]^2-^ | C_20_H_22_N_10_O_13_P_2_^2-^ | 336.0424 | 336.0427 | 14.0 | -0.89 |

- MS/MS

| Cleavage | Ion | Formula | *m/z* obs. | *m/z* theo. | RDB | ∆ppm |
| --- | --- | --- | --- | --- | --- | --- |
| a | a_1_, [X-H]^-^ | C_15_H_18_N_5_O_13_P_2_^-^ | 538.0407 | 538.0382 | 8.5 | 4.64 |
|  | a_2_, [X-H]^-^ | C_5_H_4_N_5_^-^ | 134.0479 | 134.0472 | 6.5 | 5.23 |
| b | b_1_, [X-H]^-^ | C_15_H_18_N_5_O_12_P_2_^-^ | 522.0459 | 522.0433 | 8.5 | 4.98 |
|  | b_2_, [X-H]^-^ | C_5_H_4_N_5_O^-^ | 150.0427 | 150.0421 | 6.5 | 4.00 |
| c | c_1_, [X-H]^-^ | C_10_H_11_N_5_O_7_P^-^ | 344.0412 | 344.0402 | 7.5 | 2.91 |
|  | c_2_, [X-H]^-^ | C_10_H_11_N_5_O_6_P^-^ | 328.0464 | 328.0452 | 7.5 | 3.65 |

**Characterization of standard 3´,2´-cGAMP:**

- Full HRMS (-ESI):

| Ion | Formula | *m/z* obs. | *m/z* theo. | RDB | ∆ppm |
| --- | --- | --- | --- | --- | --- |
| [M-H]^-^ | C_20_H_23_N_10_O_13_P_2_^-^ | 673.0924 | 673.0916 | 13.5 | 1.20 |
| [M-2H]^2-^ | C_20_H_22_N_10_O_13_P_2_^-^ | 336.0425 | 336.0427 | 14.0 | -0.59 |

- MS/MS:

| Cleavage | Ion | Formula | *m/z* obs. | *m/z* theo. | RDB | ∆ppm |
| --- | --- | --- | --- | --- | --- | --- |
| a | a_1_, [X-H]^-^ | C_15_H_18_N_5_O_13_P_2_^-^ | 538.0402 | 538.0382 | 8.5 | 3.71 |
|  | a_2_, [X-H]^-^ | C_5_H_4_N_5_^-^ | 134.0482 | 134.0472 | 6.5 | 7.46 |
| b | b_1_, [X-H]^-^ | C_15_H_18_N_5_O_12_P_2_^-^ | 522.0407 | 522.0433 | 8.5 | -4.86 |
|  | b_2_, [X-H]^-^ | C_5_H_4_N_5_O^-^ | 150.0430 | 150.0421 | 6.5 | 5.77 |
| c | c_1_, [X-H]^-^ | C_10_H_11_N_5_O_7_P^-^ | 344.0413 | 344.0402 | 7.5 | 3.39 |
|  | c_2_, [X-H]^-^ | C_10_H_11_N_5_O_6_P^-^ | 328.0473 | 328.0452 | 7.5 | 6.32 |

**Characterization of standard 3´,3´-cyclic-dAMP:**

- Full HRMS (-ESI):

| Ion | Formula | *m/z* obs. | *m/z* theo. | RDB | ∆ppm |
| --- | --- | --- | --- | --- | --- |
| [M-H]^-^ | C_20_H_23_N_10_O_12_P_2_^-^ | 657.0986 | 657.0978 | 13.5 | 1.21 |
| [M-2H]^2-^ | C_20_H_22_N_10_O_13_P_2_^2-^ | 328.0450 | 328.0452 | 14.0 | -0.61 |

- MS/MS

| Cleavage | Ion | Formula | *m/z* obs. | *m/z* theo. | RDB | ∆ppm |
| --- | --- | --- | --- | --- | --- | --- |
| a | a_1_, [X-H]^-^ | C_15_H_18_N_5_O_12_P_2_^-^ | 522.0469 | 522.0433 | 8.5 | 6.89 |
|  | a_2_, [X-H]^-^ | C_5_H_4_N_5_^-^ | 134.0480 | 134.0472 | 6.5 | 5.97 |
| b | b [X-H]^-^ | C_10_H_11_N_5_O_6_P^-^ | 328.0465 | 328.0452 | 7.5 | 3.94 |

**Characterization of standard 3´,3´-cyclic-dGMP:**

- Full HRMS (-ESI):

| Ion | Formula | *m/z* obs. | *m/z* theo. | RDB | ∆ppm |
| --- | --- | --- | --- | --- | --- |
| [M-H]^-^ | C_20_H_23_N_10_O_14_P_2_^-^ | 689.0875 | 689.0876 | 13.5 | -0.14 |
| [M-2H]^2-^ | C_20_H_22_N_10_O_14_P_2_^2-^ | 344.0388 | 344.0402 | 14.0 | -4.07 |

- MS/MS

| Cleavage | Ion | Formula | *m/z* obs. | *m/z* theo. | RDB | ∆ppm |
| --- | --- | --- | --- | --- | --- | --- |
| a | a_1_, [X-H]^-^ | C_15_H_18_N_5_O_12_P_2_^-^ | 538.0367 | 538.0382 | 8.5 | -2.71 |
|  | a_2_, [X-H]^-^ | C_5_H_4_N_5_O^-^ | 150.0416 | 150.0421 | 6.5 | -3.24 |
| b | b [X-H]^-^ | C_10_H_11_N_5_O_7_P^-^ | 344.0394 | 344.0402 | 7.5 | -2.28 |

**Analysis of WT enzyme product:**

- Full HRMS (-ESI):

| Ion | Formula | *m/z* obs. | *m/z* theo. | RDB | ∆ppm |
| --- | --- | --- | --- | --- | --- |
| [M-H]^-^ | C_20_H_23_N_10_O_13_P_2_^-^ | 673.0921 | 673.0916 | 13.5 | 0.74 |
| [M-2H]^2-^ | C_20_H_22_N_10_O_13_P_2_^2-^ | 336.0416 | 336.0427 | 14.0 | -3.27 |

- MS/MS

| Ion | Formula | *m/z* obs. | *m/z* theo. | RDB | ∆ppm |
| --- | --- | --- | --- | --- | --- |
| [X-H]^-^ | C_15_H_18_N_5_O_13_P_2_^-^ | 538.0374 | 538.0382 | 8.5 | -1.38 |
| [X-H]^-^ | C_15_H_18_N_5_O_12_P_2_^-^ | 522.0438 | 522.0433 | 8.5 | 1.09 |
| [X-H]^-^ | C_10_H_11_N_5_O_7_P^-^ | 344.0400 | 344.0402 | 7.5 | -0.39 |
| [X-H]^-^ | C_10_H_11_N_5_O_6_P^-^ | 328.0451 | 328.0452 | 7.5 | -0.45 |
| [X-H]^-^ | C_5_H_4_N_5_O^-^ | 150.0421 | 150.0421 | 6.5 | -0.23 |
| [X-H]^-^ | C_5_H_4_N_5_^-^ | 134.0471 | 134.0472 | 6.5 | -0.75 |

**POSITIVE ION MODE ACQUISITION**

**Characterization of standard 3´,2´-cGAMP:**

- Full HRMS (+ESI):

| Ion | Formula | *m/z* obs. | *m/z* theo. | RDB | ∆ppm |
| --- | --- | --- | --- | --- | --- |
| [M+H]^+^ | C_20_H_25_N_10_O_13_P_2_^+^ | 675.1079 | 675.1072 | 12.5 | 0.99 |
| [M+2H]^2+^ | C_20_H_26_N_10_O_13_P_2_^2+^ | 338.0562 | 338.0572 | 12.0 | -2.95 |

**Characterization of standard 3´,3´-cGAMP:**

- Full HRMS (+ESI):

| Ion | Formula | *m/z* obs. | *m/z* theo. | RDB | ∆ppm |
| --- | --- | --- | --- | --- | --- |
| [M+H]^+^ | C_20_H_25_N_10_O_13_P_2_^+^ | 675.1062 | 675.1072 | 12.5 | -1.48 |
| [M+2H]^2+^ | C_20_H_26_N_10_O_13_P_2_^2+^ | 338.0556 | 338.0572 | 12.0 | -4.73 |

**Characterization of standard 3´,3´-cyclic-dAMP:**

- Full HRMS (+ESI):

| Ion | Formula | *m/z* obs. | *m/z* theo. | RDB | ∆ppm |
| --- | --- | --- | --- | --- | --- |
| [M+H]^+^ | C_20_H_25_N_10_O_12_P_2_^+^ | 659.1123 | 659.1123 | 12.5 | -0.05 |
| [M+2H]^2+^ | C_20_H_26_N_10_O_12_P_2_^2+^ | 330.0584 | 330.0598 | 12.0 | -4.24 |

**Characterization of standard 3´,3´-cyclic-dGMP:**

- Full HRMS (+ESI):

| Ion | Formula | *m/z* obs. | *m/z* theo. | RDB | ∆ppm |
| --- | --- | --- | --- | --- | --- |
| [M+H]^+^ | C_20_H_25_N_10_O_14_P_2_^+^ | 691.0998 | 691.1021 | 12.5 | -3.32 |
| [M+2H]^2+^ | C_20_H_26_N_10_O_14_P_2_^2+^ | 346.0528 | 346.0547 | 12.0 | -5.49 |

**Analysis of WT enzyme product:**

- Full HRMS (+ESI):

| Ion | Formula | *m/z* obs. | *m/z* theo. | RDB | ∆ppm |
| --- | --- | --- | --- | --- | --- |
| [M+H]^+^ | C_20_H_25_N_10_O_13_P_2_^+^ | 675.1068 | 675.1072 | 12.5 | -0.59 |
| [M+2H]^2+^ | C_20_H_26_N_10_O_13_P_2_^2+^ | 338.0557 | 338.0572 | 12.0 | -4.43 |

**Proposed synthesis pathway of 3’2’-cGAMP by Ssc-CdnE03**

The following pathway is consistent with the products obtained after using different radiolabeled NTPs as substrates (Figs. S2D, S2G), as well as after P1 nuclease digestion of the reaction products (Fig. S2G)


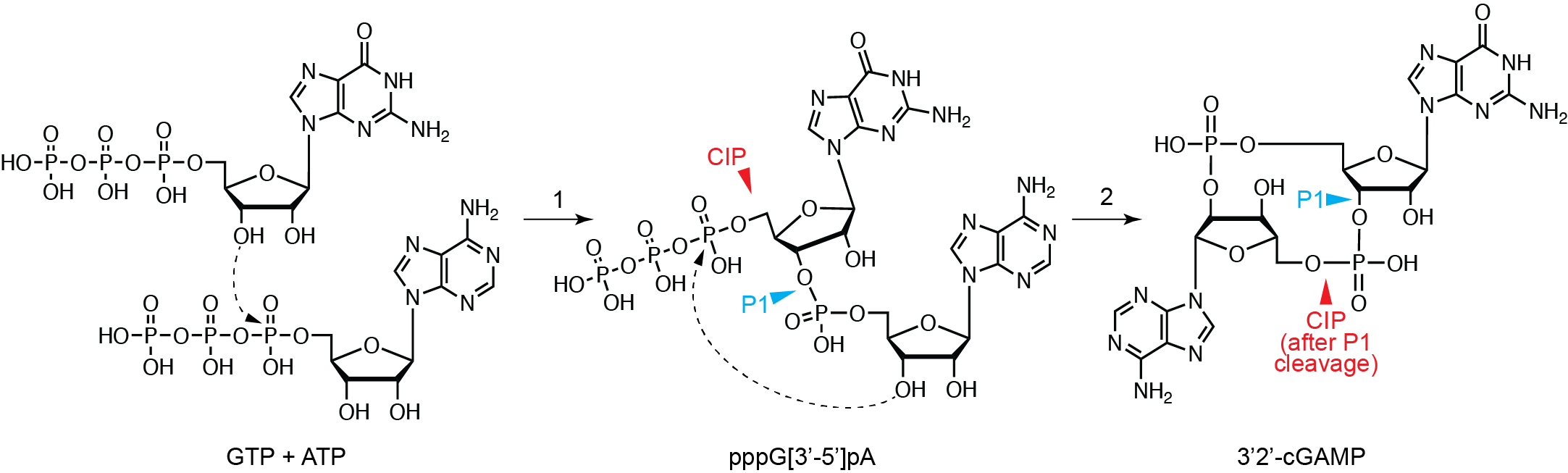

Supplement: Supplementary file 4 — Mass spectrometry analysis of the products of the Ssc-CdnE03 cyclase. [file 41586_2023_6743_MOESM4_ESM.docx]
