# Supplementary material for: Bacterial cGAS senses a viral RNA to initiate immunity
Source: Nature. 2023 Nov 15;623(7989):1001–8. doi: 10.1038/s41586-023-06743-9 (PMC10686824; doi:10.1038/s41586-023-06743-9)
Supplement: Supplementary file 5 — Supplementary Tables 1-5. [file 41586_2023_6743_MOESM5_ESM.docx]

**SUPPLEMENTARY TABLES.**

**Supplementary Methods Table 1.** Bacterial strains used in this study.

| **Species** | **Strain** | **Genotype** | **Origin** |
| --- | --- | --- | --- |
| *S. aureus* | RN4220 | Wild type | Kreiswerth et al., Nature (1983) |
| *S. aureus* | RN4220 | ::Ssc-CdnE03-cmR | Chromosomal integration (see Methods) |
| *S. aureus* | RN4220 | ::Ssc-CBASS-cmR | Chromosomal integration (see Methods) |
| *E. coli* | BL21 (DE3) | F^–^ *ompT* *gal* *dcm* *lon* *hsdS_B_* (*r_B_*^–^*m_B_*^–^) | Thermo Fisher Scientific |

**Supplementary Methods Table 2.** Phages used in this study.

| **Phage** | **Host** | **Genotype** | **Origin** |
| --- | --- | --- | --- |
| Φ80α-vir | *S. aureus* | Wild type | This study; strictly lytic mutant of Φ80α isolated from type III CRISPR-Cas targeting of *cI* repressor gene |
| Φ80α-vir(*terS*^S74F^) | *S. aureus* | *terS* S74F (C221>T) | This study; isolated from EMS chemical mutagenesis screen for Ssc-CBASS escapers |
| Φ80α-vir(*gp46*^E105D^) | *S. aureus* | *gp46* E105D (G315>T) | This study; isolated from EMS chemical mutagenesis screen for Ssc-CBASS escapers |
| Φ80α-vir(*gp46*^E105K^) | *S. aureus* | *gp46* E105K (G313>A) | This study; isolated from EMS chemical mutagenesis screen for Ssc-CBASS escapers |
| Φ80α-vir(*gp46*^R110H^) | *S. aureus* | *gp46* R110H (G329>A) | This study; isolated from EMS chemical mutagenesis screen for Ssc-CBASS escapers |
| Φ80α-vir(cabRNA^57^) | *S. aureus* | 57 silent recoding mutations along the cabRNA sequence; see Supplementary Sequences File | This study; isolated through recombination with pDVB443 followed by type II CRISPR-Cas targeting counter-selection on pDVB444 |
| Φ80α-vir(cabRNA^100^) | *S. aureus* | 100 silent recoding mutations along the cabRNA sequence; see Supplementary Sequences File | This study; isolated through recombination with pDVB442 followed by type II CRISPR-Cas targeting counter-selection on pDVB444 |
| Φ80α-vir(cabRNA^122^) | *S. aureus* | 122 silent recoding mutations along the cabRNA sequence; see Supplementary Sequences File | This study; isolated through recombination with pDVB460 followed by type II CRISPR-Cas targeting counter-selection on pDVB444 |
| Φ80α-vir^GFP^ | *S. aureus* | *gfp* inserted in between *gp18* and *gp19* | This study; isolated through recombination with pDVB434 followed by type II CRISPR-Cas targeting counter-selection on pDVB435 |
| ΦNM1γ6 | *S. aureus* | Wild type | Goldberg et al., Nature (2014) |
| ΦNM1γ6(*terS*^S74F^) | *S. aureus* | Φ80α *terS* S74F | This study; isolated through recombination with Φ80α-vir *terS^S74F^* |
| ΦNM4γ4 | *S. aureus* | Wild type | Heler et al., Nature (2015) |
| Φ12γ3 | *S. aureus* | Wild type | Modell et al., Nature (2017) |
| ΦJ1 | *S. aureus* | Wild type | This study; isolated from induction of prophage from *S. aureus* CC5 strain NRS52 |
| ΦJ2 | *S. aureus* | Wild type | This study; isolated from induction of prophage from *S. aureus* CC25 strain NRS102 |
| ΦJ4 | *S. aureus* | Wild type | This study; isolated from induction of prophage from *S. aureus* CC5 strain NRS110 |

**Supplementary Methods Table 3.** Plasmids used in this study.

| **Plasmid** | **Description** | **Source** | **Construction Notes** |
| --- | --- | --- | --- |
| pDVB08 | *S. epidermidis* RP62a type III-A CRISPR-Cas system with programmed spacer targeting Φ80α *cI* gene | This study | Ligation of BsaI-digested pGG78 and annealed oDVB16/oDVB17 |
| pDVB301 | Ssc-CdnE03 (cyclase only) with native promoter on pC194-based vector | This study | Gibson Assembly: oDVB401+oDVB402 (pDVB47 template), oDVB405+oDVB406 (*S. schleiferi* 2142-05 genomic DNA template) |
| pDVB302 | Ssc-Cap15 (effector only) with native promoter on pC194-based vector | This study | Gibson Assembly: oDVB401+oDVB402 (pDVB47 template), oDVB407+oDVB408 (*S. schleiferi* 2142-05 genomic DNA template) |
| pDVB303 | Ssc-CBASS (full system) with native promoter on pC194-based vector | This study | Gibson Assembly: oDVB401+oDVB402 (pDVB47 template), oDVB405+oDVB408 (*S. schleiferi* 2142-05 genomic DNA template) |
| pDVB313 | Ssc-CBASS with native promoter and catalytically inactive Ssc-CdnE03 (D86A, D88A mutation) on pC194-based vector | This study | Gibson Assembly: oDVB459+oDVB460 (pDVB303 template) |
| pDVB317 | His6-MBP Ssc-CdnE03 on IPTG-inducible pET-based vector for recombinant protein expression | This study | Gibson Assembly: oDVB479+oDVB480 (pET His6 MBP TEV LIC cloning vector template),  oDVB481+oDVB482 (*S. schleiferi* 2142-05 genomic DNA template) |
| pDVB318 | His6-MBP Ssc-CdnE03 (D86A, D88A mutation) on IPTG-inducible pET-based vector for recombinant protein expression | This study | Gibson Assembly: oDVB459+oDVB460 (pDVB317 template) |
| pDVB374 | Φ80α *gp46* on IPTG-inducible pE194-based vector with strong RBS (also denoted as “pGp46”) | This study | Gibson Assembly: oCR95+oCR96 (pPM134 template), oDVB624+oDVB625 (Φ80α-vir genomic DNA template) |
| pDVB377 | Φ80α *terS* on IPTG-inducible pE194-based vector with strong RBS (also denoted “pTerS”) | This study | Gibson Assembly: oCR95+oCR96 (pPM134 template), oDVB628+oDVB629 (Φ80α-vir genomic DNA template) |
| pDVB377 (*terS^S74F^)* | Φ80α *terS* S74F on IPTG-inducible pE194-based vector with strong RBS (also denoted “pTerS^S74F^”) | This study | Gibson Assembly: oCR95+oCR96 (pPM134 template), oDVB628+oDVB629 (Φ80α-vir *terS^S74F^* genomic DNA template) |
| pDVB378 | Φ80α *gp40-gp47* on IPTG-inducible pE194-based vector with strong RBS | This study | Gibson Assembly: oCR95+oCR96 (pPM134 template), oDVB626+oDVB628 (Φ80α-vir genomic DNA template) |
| pDVB392 | Ssc-CBASS with native promoter and Ssc-CdnE03 (K9E mutation) on pC194-based vector | This study | Gibson Assembly: oDVB56+oDVB695 (pDVB303 template), oDVB57+oDVB696 (pDVB303 template) |
| pDVB393 | Ssc-CBASS with native promoter and Ssc-CdnE03 (K13E mutation) on pC194-based vector | This study | Gibson Assembly: oDVB56+oDVB697 (pDVB303 template), oDVB57+oDVB698 (pDVB303 template) |
| pDVB396 | His6-MBP Ssc-CdnE03 (K9E mutation) on IPTG-inducible pET-based vector for recombinant protein expression | This study | Gibson Assembly: oDVB695+oDVB705 (pDVB317 template), oDVB696+oDVB706 (pDVB317 template) |
| pDVB397 | His6-MBP Ssc-CdnE03 (K13E mutation) on IPTG-inducible pET-based vector for recombinant protein expression | This study | Gibson Assembly: oDVB697+oDVB705 (pDVB317 template), oDVB698+oDVB706 (pDVB317 template) |
| pDVB400 | 400-bp cabRNA from Φ80α-vir (sense direction) on aTc-inducible pE194-based vector with terminators  (also denoted “pcabRNA”) | This study | Gibson Assembly:  oDVB461+oDVB462 (pJTR162 template), oDVB679+oDVB680 (Φ80α-vir genomic DNA template) |
| pDVB401 | 400-bp RNA from Φ80α-vir (antisense direction) on aTc-inducible pE194-based vector with terminators | This study | Gibson Assembly:  oDVB461+oDVB462 (pJTR162 template), oDVB681+oDVB682 (Φ80α-vir genomic DNA template) |
| pDVB434 | Recombination plasmid harboring *gfp* gene and strong RBS with 500-nt upstream and downstream homology arms corresponding to Φ80α *gp18* and *gp19*, respectively | This study | Gibson Assembly:  oDVB768+oDVB769 (pCFB55 template), oDVB770+oDVB771 (Φ80α-vir genomic DNA template), oDVB772+oDVB773 (pCFB55 template), oDVB774+oDVB775 (Φ80α-vir genomic DNA template) |
| pDVB435 | *S. aureus* M06/0171 type II-A CRISPR-Cas system with programmed spacer targeting wild-type Φ80α-vir but not Φ80α-vir::GFP (*gfp* insertion between protospacer and PAM) | This study | Ligation of BsaI-digested pDVB47 and annealed oDVB776/oDVB777 |
| pDVB437 | His6-MBP Ssc-CdnE03 (K9E and K13E mutations) on IPTG-inducible pET-based vector for recombinant protein expression | This study | Gibson Assembly:  oDVB705+oDVB778 (pDVB317 template), oDVB706+oDVB779 (pDVB317 template) |
| pDVB442 | Recombination plasmid harboring Φ80α cabRNA sequence with 100 silent recoding mutations at the wobble position with 500-nt upstream and downstream homology arms | This study | Gibson Assembly:  oDVB768+oDVB769 (pDVB434 template), oDVB787+oDVB788 (Φ80α-vir genomic DNA template), oDVB789+oDVB791 (synthesized Gene block for recoded Φ80α-vir cabRNA^100^), oDVB792+oDVB793 (Φ80α-vir genomic DNA template) |
| pDVB443 | Recombination plasmid harboring Φ80α cabRNA sequence with 57 silent recoding mutations at the wobble position with 500-nt upstream and downstream homology arms | This study | Gibson Assembly:  oDVB768+oDVB769 (pDVB434 template), oDVB787+oDVB788 (Φ80α-vir genomic DNA template), oDVB790+oDVB791 (synthesized Gene block for recoded Φ80α-vir cabRNA^57^), oDVB792+oDVB793 (Φ80α-vir genomic DNA template) |
| pDVB444 | *S. aureus* M06/0171 type II-A CRISPR-Cas system with programmed spacer targeting wild-type Φ80α cabRNA but not recoded sequences (NNGRRT PAM mismatch) | This study | Ligation of BsaI-digested pDVB47 and annealed oDVB794/oDVB795 |
| pDVB445 | Sha-CBASS (full system) on IPTG-inducible pE194-based vector with strong RBS | This study | Gibson Assembly:  oCR95+oCR96  (pDVB377 template), oDVB800+oDVB801 (synthesized Gene block for Sha-CBASS harboring CdnE01 and Cap15) |
| pDVB455 | His6-TEV Sha-CdnE01 on IPTG-inducible pET-based vector for recombinant protein expression | This study | Gibson Assembly:  oDVB705+oDVB815 (pDVB412 template), oDVB706+oDVB816 (pDVB412 template), oDVB817+oDVB818 (pDVB445 template) |
| pDVB460 | Recombination plasmid harboring Φ80α cabRNA sequence with 122 silent recoding mutations at the wobble position with 500-nt upstream and downstream homology arms | This study | Gibson Assembly:  oDVB56+oDVB827 (pDVB442 template), oDVB57+oDVB828 (pDVB442 template) |
| pCR35 | Ssc-CdnE03 (cyclase only) on IPTG-inducible pE194-based vector | This study | Gibson Assembly: oCR95+oCR96 (pPM134 template), oCR98+oCR100 (*S. schleiferi* 2142-05 genomic DNA template) |
| pCR36 | Ssc-CBASS (full system) on IPTG-inducible pE194-based vector | This study | Gibson Assembly: oCR95+oCR96 (pPM134 template), oCR99+oCR100 (*S. schleiferi* 2142-05 genomic DNA template) |
| pCR37 | Ssc-Cap15 (effector only) on IPTG-inducible pE194-based vector | This study | Gibson Assembly: oCR95+oCR96 (pPM134 template), oCR99+oCR111 (*S. schleiferi* 2142-05 genomic DNA template) |

**Supplementary Methods Table 4.** Oligonucleotide primers used in this study.

| **Primer** | **Sequence** |
| --- | --- |
| oDVB16 | GAACATTGCTCGTTTGCATAGTTAAGCACATTTTG |
| oDVB17 | CGATCAAAATGTGCTTAACTATGCAAACGAGCAAT |
| oDVB56 | ATCATAAAACTCTTTGAAGTCATTCTTTACAGGAGTCCAAATACCAGAGAATGTTTTAGA |
| oDVB57 | GTAAAGAATGACTTCAAAGAGTTTTATGATTTATACCTTTCTGATGTAGAGAAATATAAT |
| oDVB401 | CAAACGAAAATTGGATAAAGTGGGA |
| oDVB402 | TCGTTTGTTGAACTAATGGGTGCTT |
| oDVB405 | AAGCACCCATTAGTTCAACAAACGAGACTTTTCTTGTGATTTTCTTTGCGAAAAATATTG |
| oDVB406 | TCCCACTTTATCCAATTTTCGTTTGTCATTCATATTTTTTCTCACCACTATATTCAAAAT |
| oDVB407 | AAGCACCCATTAGTTCAACAAACGATAGATGTAAAGAATTATTTTGAATATAGTGGTGAG |
| oDVB408 | TCCCACTTTATCCAATTTTCGTTTGAAGCTTATCATAAATGATGTGGTTTTTGATAAGGT |
| oDVB426 | GCGCCTTCACGAATTTGTTC |
| oDVB427 | CACCGTTCTGGTTCGAGTTT |
| oDVB459 | CACTGCAATTGCAACTGCACTATTTTGTCTTACATTGGTATTTGTTGCATACG |
| oDVB460 | CAAAATAGTGCAGTTGCAATTGCAGTGGTAAAAGAAAGTGAGTTTTTTGATAAATAT |
| oDVB461 | CTCCTAGGTCATTTGATATGCCTCCGGATATCACTCTATCAATGATAGAGAGCTTATTTT |
| oDVB462 | CTAGATAAACCTTCAGACGTACATACATTTACTCCTAAATACCATAAATTAGCTGAGGCG |
| oDVB479 | GGATTGGAAGTACAGGTTTTCCTCGATCCCATTAGTCTGCGCGTCTTTCAGGGCTTCATC |
| oDVB480 | GATCCGGCTGCTAACAAAGCCCGAAAGGAAGCTGAGTTGGCTGCTGCCACCGCTGAGCAA |
| oDVB481 | GGGATCGAGGAAAACCTGTACTTCCAATCCTTGTTATTTACTGAAGAACAATTAAAATTA |
| oDVB482 | TTCCTTTCGGGCTTTGTTAGCAGCCGGATCTCATTCATATTTTTTCTCACCACTATATTC |
| oDVB565 | GGTGTTGAAACGCGATACTTTTCTAATAATGATAGCGAACTATTGAAGAGTCACATGTTTTATTGGAGTGGACTTTTCTTGTGATTTTCT |
| oDVB566 | GCTAATTGACAAGGTCTCATAAATGACTCAGCAAACGATTGCAATGTATTGATACGGTTATTCTGTTTATTTATAAAAGCCAGTCATTAG |
| oDVB610 | TGTTTCTAGTAATAAGCCTCCGTATC |
| oDVB611 | CCTCTTCTTACCTCACTTCTTCC |
| oDVB614 | TCATGTACGGTATGGCAAATAGG |
| oDVB615 | ACAATTTCCACTTCCACATAAACC |
| oDVB624 | TTAAGCTTGTACTTAGGAGGATGATTATTTATGGAAGAAAATAAACTTAAGTTTAATTTG |
| oDVB625 | CCGATTGCAGTATAAATTTAACGATCACTCTTAATTTTTAATAATTCTTTTTTGTCTAGC |
| oDVB626 | CCGATTGCAGTATAAATTTAACGATCACTCTTAAACTTCTCCTGGAACTGAATCTGTTCT |
| oDVB628 | TTAAGCTTGTACTTAGGAGGATGATTATTTATGAACGAAAAACAAAAGAGATTCGCAGAT |
| oDVB629 | CCGATTGCAGTATAAATTTAACGATCACTCTTAACTTTCGTCATCGTACTCACCAATATT |
| oDVB679 | TATCCGGAGGCATATCAAATGACCTAGGAGAGAGGAGAACCTCAAGAGGCTTACAGTAAG |
| oDVB680 | AAATGTATGTACGTCTGAAGGTTTATCTAGTCTAAGCCATAGTATACGCCTAGGATATTT |
| oDVB681 | AAATGTATGTACGTCTGAAGGTTTATCTAGAGAGGAGAACCTCAAGAGGCTTACAGTAAG |
| oDVB682 | TATCCGGAGGCATATCAAATGACCTAGGAGTCTAAGCCATAGTATACGCCTAGGATATTT |
| oDVB691 | TAATACGACTCACTATAATGACTAAAAAGAAATATGG |
| oDVB695 | CTGAAGAACAATTAGAATTATATTCTAAACCATTGTCAGAATCTGAAAAAGAAAA |
| oDVB696 | TTCTGACAATGGTTTAGAATATAATTCTAATTGTTCTTCAGTAAATAACAA |
| oDVB697 | ACAATTAAAATTATATTCTGAACCATTGTCAGAATCTGAAAAAGAAAAGT |
| oDVB698 | ATTCTGACAATGGTTCAGAATATAATTTTAATTGTTCTTCAGTAAATAACAA |
| oDVB705 | TTGCATTCGATTCCTGTTTGTAATTGTCC |
| oDVB706 | GGACAATTACAAACAGGAATCGAATGCAA |
| oDVB768 | ATATATTTATGTTACAGTAATATTGACTTTTAAAAAAGGATTGA |
| oDVB769 | ATTTTAAAAATATCCCACTTTATCCAATTTTCGT |
| oDVB770 | AAATTGGATAAAGTGGGATATTTTTAAAATAGGTCAACCAATGGACTATATGC |
| oDVB771 | CTTTACTCATTGATTAACTCCTCCTTAAAAAGGAGTGCTATTTTTATTATTCTTAGTCTA |
| oDVB772 | TTTTAAGGAGGAGTTAATCAATGAGTAAAGG |
| oDVB773 | TTATTTGTATAGTTCATCCATGCCATGT |
| oDVB774 | ACACATGGCATGGATGAACTATACAAATAAAATCGTCATCTTGGCGGAA |
| oDVB775 | TTTTAAAAGTCAATATTACTGTAACATAAATATATAAATATAGAGCGGTTTTAAACCCAA |
| oDVB776 | AGACTTTCTAATCTCTTCCGCCAAGATGACGATTG |
| oDVB777 | AAAACAATCGTCATCTTGGCGGAAGAGATTAGAAA |
| oDVB778 | AACAATTAGAATTATATTCTGAACCATTGTCAGAATCTGAAAAAG |
| oDVB779 | GACAATGGTTCAGAATATAATTCTAATTGTTCTTCAGTAAATAAC |
| oDVB787 | AAATTGGATAAAGTGGGATATTTTTAAAATATGACATACACATTTCTGATAGAACTG |
| oDVB788 | AGCAATAGAAGCAGATAACGC |
| oDVB789 | GAAGCTTTAGCGTTATCTGCTTCTATTGCTAGGGGGGAGCCCCAAGAA |
| oDVB790 | GAAGCTTTAGCGTTATCTGCTTCTATTGCTAGAGGAGAACCTCAAGAGGCT |
| oDVB791 | CCTGAGCCACAGTATGCGTC |
| oDVB792 | AAGTACCCCAGACGCATACTGTGGCTCAGGAAAGTCCAATCAACAATTAAAGATAGTTTA |
| oDVB793 | TTTTAAAAGTCAATATTACTGTAACATAAATATATAATTTGTCTAGTGTAGAAAATTCAC |
| oDVB794 | AGACTCAGTGAAGTTATCGTAATTGGTTAGTATTG |
| oDVB795 | AAAACAATACTAACCAATTACGATAACTTCACTGA |
| oDVB800 | TTAAGCTTGTACTTAGGAGGATGATTATTTATGACTATACCTACAAAAAAATTGGATTAT |
| oDVB801 | TGCAGTATAAATTTAACGATCACTCTTATGATTCAATAACTTTTTTTAAATTTAGCTTAC |
| oDVB815 | GATCCGGCTGCTAACAAAGC |
| oDVB816 | GGATTGGAAGTACAGGTTTTCAGAAG |
| oDVB817 | TTCTGAAAACCTGTACTTCCAATCCATGACTATACCTACAAAAAAATTGGAT |
| oDVB818 | TTCGGGCTTTGTTAGCAGCCGGATCTTACCACTCATTCCAAAATTTATTTATTTCAAA |
| oDVB827 | AGGAGATCACCCAAAAAAACATCGAAATCAACATCGGCGAATATGATGACGAAAGTTAAGTTGAATTTCAA |
| oDVB828 | TCGATGTTTTTTTGGGTGATCTCCTTCTTATCAATGTATGCGCCGTGGACCTTCAGGATATGATCGATGG |
| oCR95 | AAATAATCATCCTCCTAAGTACAAGCTTAATTGTTATCCGCTCACAATTCCACACATTAT |
| oCR96 | GAGTGATCGTTAAATTTATACTGCAATCGGATGCGATTATTGAATAAAAGATATGAGAGA |
| oCR98 | CCGATTGCAGTATAAATTTAACGATCACTCTCATTCATATTTTTTCTCACCACTATATTC |
| oCR99 | CCGATTGCAGTATAAATTTAACGATCACTCTTATTTTAGTTTTTTGAGTCTTAGATCACC |
| oCR100 | TTAAGCTTGTACTTAGGAGGATGATTATTTTTGTTATTTACTGAAGAACAATTAAAATTA |
| oCR111 | TTAAGCTTGTACTTAGGAGGATGATTATTTATGAATGACAAAATAAATCATTTGATTAAA |
| oCR190 | TAATACGACTCACTATAAGAGGAGAACCTCAAGAGGC |
| oCR191 | TAATACGACTCACTATATCTAAGCCATAGTATACGCCTAGG |
| oCR192 | AGAGGAGAACCTCAAGAGGC |
| oCR193 | TCTAAGCCATAGTATACGCCTAGG |

**Supplementary Methods Table 5.** RNA oligonucleotides used in this study.

| **RNA oligo** | **Sequence** | **Description** |
| --- | --- | --- |
| oCR114 | GCUAAACAAACAGCAAUAGAGUACGUACAAGGCUUCUCUACAAAA | Random 45-nt hairpin-forming ssRNA for *in vitro* nucleotide synthesis assays |
| oCR150 | AAUAAACAAACAGCAAUAGAGUACGUACAAGGCUUGUCUACAAAA | ssRNA for *in vitro* nucleotide synthesis assays |
| oCR151 | UUUUGUAGACAAGCCUUGUACGUACUCUAUUGCUGUUUGUUUAUU | dsRNA for *in vitro* nucleotide synthesis assays; sequence of the bottom strand is displayed |
| oCR198 | UACGAUAACUUCACUGAAGUACAUUACGGUGGAGGUUCGAG | ssRNA hairpin #1 from Φ80α-vir cabRNA for *in vitro* nucleotide synthesis assays |
| oCR199 | AUUACUCAGAAGAAUAUUGAGAUUAAUAUUGGUGAGUACG | ssRNA hairpin #2 from Φ80α-vir cabRNA for *in vitro* nucleotide synthesis assays |
| oCR320 | GAAUGUUAUGGCAAUGCUACUAAGUCAGCUGUGC | Synthesized ΦJ1/ΦJ2 cabRNA (34-nt) corresponding to the sense direction |
| oCR321 | GCACAGCUGACUUAGUAGCAUUGCCAUAACAUUC | Synthesized ΦJ1/ΦJ2 cabRNA (34-nt) corresponding to the antisense direction |
| oCR342 | UUCCGGAAGAUGAUUAGUCAUGUAUGAAAUACUUGAUCUAAAAAAUAAA | Synthesized ΦJ4 cabRNA (49-nt) corresponding to the sense direction |
| oCR343 | UUUAUUUUUUAGAUCAAGUAUUUCAUACAUGACUAAUCAUCUUCCGGAA | Synthesized ΦJ4 cabRNA (49-nt) corresponding to the antisense direction |
