# Supplementary material for: Bacterial cGAS senses a viral RNA to initiate immunity
Source: Nature. 2023 Nov 15;623(7989):1001–8. doi: 10.1038/s41586-023-06743-9 (PMC10686824; doi:10.1038/s41586-023-06743-9)

SUPPLEMENTARY SEQUENCES FILE.

<u>Sequences of RNA species pulled down from infected cells using Ssc-CdnE03</u>

400-nt bacteriophage RNA pulled down during Φ80α-vir infection (cabRNA$_{\Phi 80\alpha}$):
5'-
AGAGGAGAACCUCAAGAGGCUUACAGUAAGAAAUAUGACCAUUUAAACGAUGAA
GUGGAAAAAGAGGUUACUUACACAAUCACACCAACUUUUGAAGAGCGUCAGAGA
UCUAUUGACCACAUACUAAAAGUUCAUGGUGCGUAUAUCGACAAAAAAGAAAUUA
CUCAGAAGAAUAUUGAGAUUAAUAUUGGUGAGUACGAUGACGAAAGUUAAAUUA
AACUUUAACAAACCAUCUAAUGUUUUCAACAGAAACAUAUUCGAAAUACUAACCA
AUUACGAUAACUUCACUGAAGUACAUUACGGUGGAGGUUCGAGUGGUAAGUCUC
ACGGCGUUAUACAAAAAGUUGUACUUAAAGCAUUGCAAGACUGGAAAUAUCCUA
GGCGUAUACUAUGGCUUAGA-3'

Predicted structure:

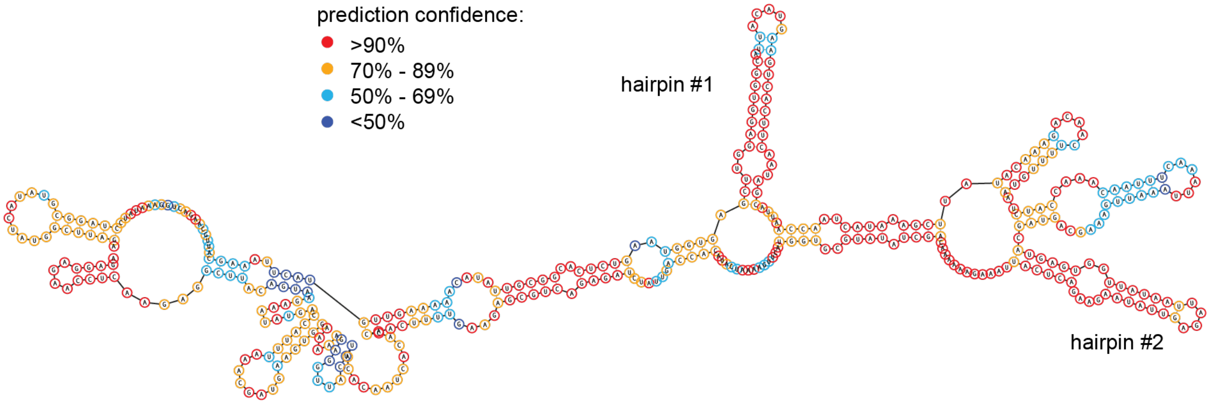

Hairpin #1 sequence:
5'-UACGAUAACUUCACUGAAGUACAUUACGGUGGAGGUUCGAG-3'

Predicted structure:

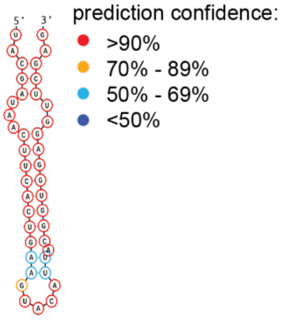

Hairpin #1 stem sequences:
5'-CUUCACUGAA-3'
3'-GAGGUGGCAUU-3'

Predicted structure:

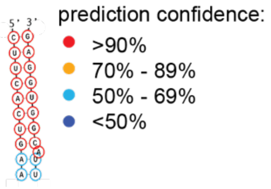

Hairpin #2 sequence:
5'-UACUCAGAAGAAUAUUGAGAUUAAUAUUGGUGAGUA-3'

Predicted structure:

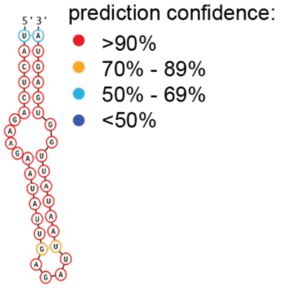

1237-nt bacteriophage RNA pulled down during Φ80α-vir *gp40*$_{\mathbf{S74F}}$ infection: 5'-
AUGACUAAAAAGAAAUAUGGAUUAAAAUUAUCAACAGUUCGAAAGUUAGAAGAUG
AGUUGUGUGAUUAUCCUAAUUAUCAUAAGCAACUCGAAGAUUUAAGAAGUGAAA
UAAUGACACCAUGGAUUCCAACAGAUACAAAUAUAGGCGGGGAGUUUGUACCGU
CUAAUACAUCGAAAACAGAAAUGGCAGUAACUAAUUAUCUUUGUAGUAUACGAAG
AGGUAAAAUCCUUGAGUUUAAGAGCGCUAUUGAACGUAUAAUCAACACAUCAAG
UAGGAAAGAACGCGAAUUCAUUCAAGAGUAUUAUUUUAAUAAAAAGGAAUUAGU
GAAAGUUUGUGAUGACAUACACAUUUCUGAUAGAACUGCUCAUAGAAUCAAAAG
GAAAAUCAUAUCUAGAUUGGCGGAAGAGUUAGGGGAAGAGUGAAAUUGGCAGUA
AAGUGGCAGUUUUUGAUACCUAAAAUGAGAUAUUAUGAUAGUGUAGGAUAUUGA
CUAUCUUACUGCGUUUCCCUUAUCGCAAUUAGGAAUAAAGGAUCUAUGUGGGUU
GGCUGAUUAUAGCCAAUCCUUUUUUAAUUUUAAAAAGCGUAUAGCGCGAGAGUU
GGUGGUAAAUGAAAUGAACGAAAAACAAAAGAGAUUCGCAGAUGAAUAUAUAAUG
AAUGGAUGUAAUGGUAAAAAAGCAGCAAUUUCAGCAGGUUAUAGUAAGAAAACA
GCAGAGUCUUUAGCAAGUCGAUUGUUAAGAAAUGUUAAUGUUUCGGAAUAUAUU
AAAGAACGAUUAGAACAGAUACAAGAAGAGCGUUUAAUGAGCAUUACAGAAGCU
UUAGCGUUAUCUGCUU**U**UAUUGCUAGAGGAGAACCUCAAGAGGCUUACAGUAAG
AAAUAUGACCAUUUAAACGAUGAAGUGGAAAAAGAGGUUACUUACACAAUCACAC
CAACUUUUGAAGAGCGUCAGAGAUCUAUUGACCACAUACUAAAAGUUCAUGGUG
CGUAUAUCGACAAAAAAGAAAUUACUCAGAAGAAUAUUGAGAUUAAUAUUGGUGA
GUACGAUGACGAAAGUUAAAUUAAACUUUAACAAACCAUCUAAUGUUUUCAACAG
AAACAUAUUCGAAAUACUAACCAAUUACGAUAACUUCACUGAAGUACAUUACGGU
GGAGGUUCGAGUGGUAAGUCUCACGGCGUUAUACAAAAAGUUGUACUUAAAGCA
UUGCAAGACUGGAAAUAUCCUAGGCGUAUACUAUGGCUUAGA-3'

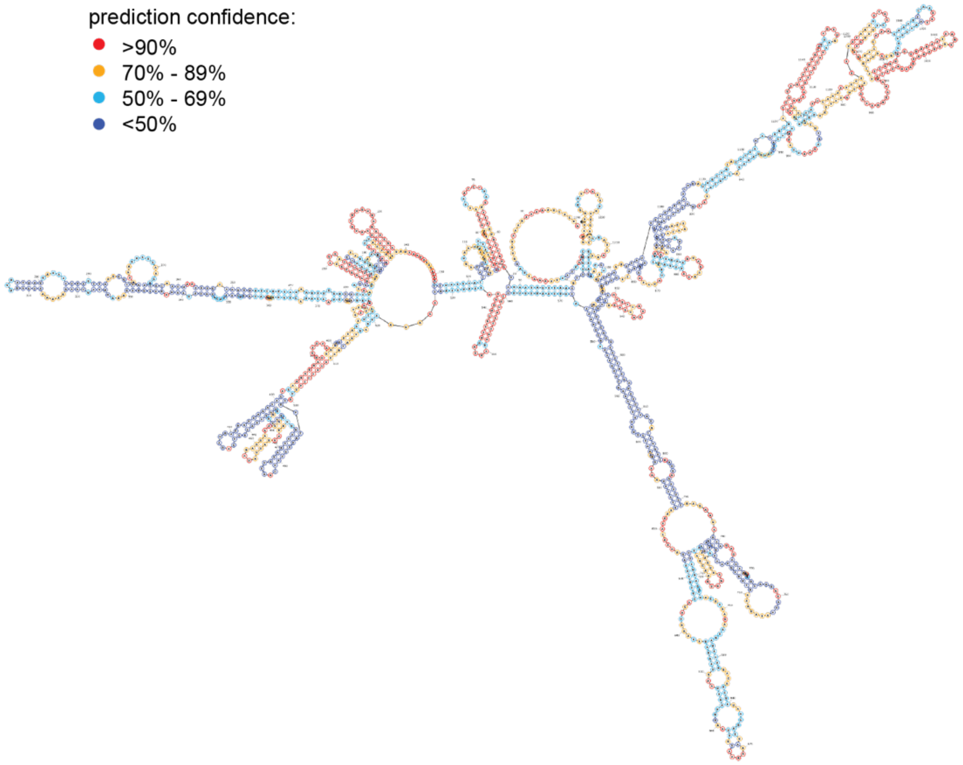

400-nt bacteriophage RNA pulled down during ΦNM1γ6 infection (cabRNA$_{\Phi NM1\gamma 6}$):
5'-
AGAGGAGAACCUCAAGAGGCUUACAGUAAGAAAUAUGACCAUUUAAACGAUGAA
GUGGAAAAAGAGGUUACUUACACAAUCACACCAACUUUUGAAGAGCGUCAGAGA
UCUAUUGACCACAUACUAAAAGUACAUGGUGCGUAUAUCGAUAAAAAAGAAAUUA
CUCAGAAGAAUAUUGAGAUUAAUAUUGGUGAGUACGAUGACGAAAGUUAAAUUA
AACUUUAACAAACCAUCUAAUGUUUUCAAUAGAAACAUAUUCGAAAUACUAACCA
AUUACGAUAACUUCACUGAAGUACAUUACGGUGGAGGUUCGAGCGGUAAGUCUC
ACGGCGUUAUACAAAAAGUUGUACUUAAAGCAUUGCAAGACUGGAAAUAUCCUA
GGCGUAUACUAUGGCUUAGA-3'

80-nt bacteriophage RNA pulled down during Φ80α-vir(cabRNA^{100}):5'-
AUGGUGCGUAUAUCGACAAAAAAGAAAUUACUCAGAAGAAUAUUGAGAUUAAUAU
UGGUGAGUACGAUGACGAAAGUUAA-3'

Predicted structure:

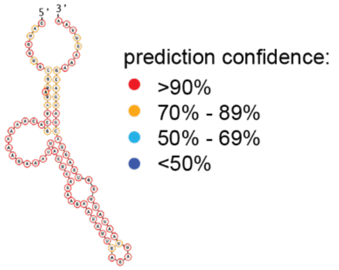

200-nt bacteriophage RNA pulled down during Φ80α-vir(cabRNA^{57}):5’-AGAGGAGAACCUCAAGAGGCUUACAGUAAGAAAUAUGACCAUUUAAACGAUGAA
GUGGAAAAAGAGGUUACUUACACAAUCACACCAACUUUUGAAGAGCGUCAGAGA
UCUAUUGACCACAUACUAAAAGUUCAUGGUGCGUAUAUCGACAAAAAAGAAAUUA
CUCAGAAGAAUAUUGAGAUUAAUAUUGGUGAGUACGA-3’

Predicted structure:

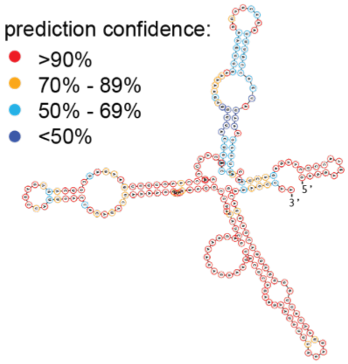

34-nt bacteriophage RNA pulled down during ΦJ1 and ΦJ2 infection (cabRNA$_{\Phi J1/2}$):

5’-GAATGTTATGGCAATGCTACTAAGTCAGCTGTGC-3’

Predicted structure:

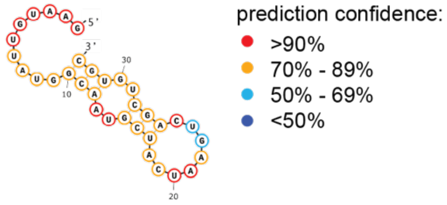

49-nt bacteriophage RNA pulled down during ΦJ4 infection (cabRNA$_{\Phi J4}$):

5’-TTCCGGAAGATGATTAGTCATGTATGAAATACTTGATCTAAAAAATAAA-3’

Predicted structure:

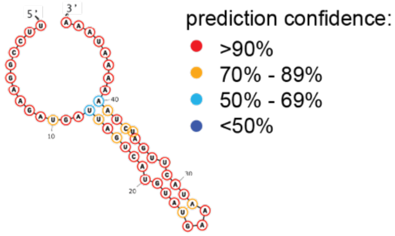

<u>Sequence of the RNA species pulled down from infected cells using Sha-CdnE01</u>

400-nt bacteriophage RNA pulled down during Φ80α-vir infection (cabRNA$_{\Phi80\alpha}$):
5'-
AGAGGAGAACCUCAAGAGGCUUACAGUAAGAAAUAUGACCAUUUAAACGAUGAA
GUGGAAAAAGAGGUUACUUACACAAUCACACCAACUUUUGAAGAGCGUCAGAGA
UCUAUUGACCACAUACUAAAAGUUCAUGGUGCGUAUAUCGACAAAAAAGAAAUUA
CUCAGAAGAAUAUUGAGAUUAAUAUUGGUGAGUACGAUGACGAAAGUUAAAUUA
AACUUUAACAAACCAUCUAAUGUUUUCAACAGAAACAUAUUCGAAAUACUAACCA
AUUACGAUAACUUCACUGAAGUACAUUACGGUGGAGGUUCGAGUGGUAAGUCUC
ACGGCGUUAUACAAAAAGUUGUACUUAAAGCAUUGCAAGACUGGAAAUAUCCUA
GGCGUAUACUAUGGCUUAGA-3'

70-nt bacteriophage RNA pulled down during Φ80α-vir infection (hairpin #1): 5'-
UAUUCGAAAUACUAACCAAUUACGAUAACUUCACUGAAGUACAUUACGGUGGAG
GUUCGAGUGGUAAGUC -3'

Predicted structure:

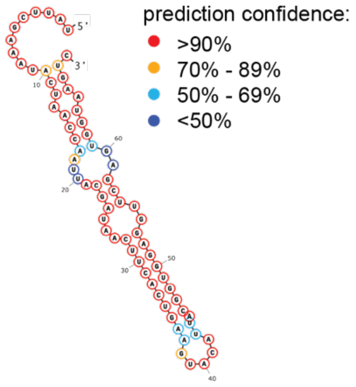

<u>Sequences of *gp40* from bacteriophages used in this study:</u>

***gp40* sequence from Φ80α-vir:** 5’-
ATGAACGAAAAACAAAAGAGATTCGCAGATGAATATATAATGAATGGATGTAATGGT
AAAAAAGCAGCAATTTCAGCAGGTTATAGTAAGAAAACAGCAGAGTCTTTAGCAAG
TCGATTGTTAAGAAATGTTAATGTTTCGGAATATATTAAAGAACGATTAGAACAGAT
ACAAGAAGAGCGTTTAATGAGCATTACAGAAGCTTTAGCGTTATCTGCTTCTATTGC
TAGAGGAGAACCTCAAGAGGCTTACAGTAAGAAATATGACCATTTAAACGATGAAG
TGGAAAAAGAGGTTACTTACACAATCACACCAACTTTTGAAGAGCGTCAGAGATCT
ATTGACCACATACTAAAAGTTCATGGTGCGTATATCGACAAAAAAGAAATTACTCAG
AAGAATATTGAGATTAATATTGGTGAGTACGATGACGAAAGTTAA-3’

***gp40* sequence from Φ80α-vir *gp40*$_{S74F}$:** 5’-
ATGAACGAAAAACAAAAGAGATTCGCAGATGAATATATAATGAATGGATGTAATGGT
AAAAAAGCAGCAATTTCAGCAGGTTATAGTAAGAAAACAGCAGAGTCTTTAGCAAG
TCGATTGTTAAGAAATGTTAATGTTTCGGAATATATTAAAGAACGATTAGAACAGAT
ACAAGAAGAGCGTTTAATGAGCATTACAGAAGCTTTAGCGTTATCTGCTT**T**TATTGC
TAGAGGAGAACCTCAAGAGGCTTACAGTAAGAAATATGACCATTTAAACGATGAAG
TGGAAAAAGAGGTTACTTACACAATCACACCAACTTTTGAAGAGCGTCAGAGATCT
ATTGACCACATACTAAAAGTTCATGGTGCGTATATCGACAAAAAAGAAATTACTCAG
AAGAATATTGAGATTAATATTGGTGAGTACGATGACGAAAGTTAA-3’

***gp40* sequence from ΦNM1γ6:** 5’-
ATGAACGAAAAACAAAAGAGATTCGCAGATGAATATATAATGAATGGATGTAATGGT
AAAAAAGCAGCAATTACAGCAGGTTATAGTAAGAAAACAGCAGAGTCTTTAGCAAG
TCGATTGTTAAGAAATGTTAATGTTTCGGAATATATTAAAGAACGATTAGAACAGAT
ACAAGAAGAGCGTTTAATGAGTATTACAGAAGCTTTAGCGTTATCTGCTTCTATTGC
TAGAGGAGAACCTCAAGAGGCTTACAGTAAGAAATATGACCATTTAAACGATGAAG
TGGAAAAAGAGGTTACTTACACAATCACACCAACTTTTGAAGAGCGTCAGAGATCT
ATTGACCACATACTAAAAGTACATGGTGCGTATATCGATAAAAAAGAAATTACTCAG
AAGAATATTGAGATTAATATTGGTGAGTACGATGACGAAAGTTAA-3’

***gp40* sequence from ΦNM1γ6 *gp40*$_{S74F}$ recombinant:** 5’-
ATGAACGAAAAACAAAAGAGATTCGCAGATGAATATATAATGAATGGATGTAATGGT
AAAAAAGCAGCAATTTCAGCAGGTTATAGTAAGAAAACAGCAGAGTCTTTAGCAAG
TCGATTGTTAAGAAATGTTAATGTTTCGGAATATATTAAAGAACGATTAGAACAGAT
ACAAGAAGAGCGTTTAATGAGCATTACAGAAGCTTTAGCGTTATCTGCTT**T**TATTGC
TAGAGGAGAACCTCAAGAGGCTTACAGTAAGAAATATGACCATTTAAACGATGAAG
TGGAAAAAGAGGTTACTTACACAATCACACCAACTTTTGAAGAGCGTCAGAGATCT
ATTGACCACATACTAAAAGTTCATGGTGCGTATATCGACAAAAAAGAAATTACTCAG
AAGAATATTGAGATTAATATTGGTGAGTACGATGACGAAAGTTAA-3’

Mutated cabRNA DNA sequences (5'-3') from Φ80α used in this study. The sequence coding TerS C-terminal amino acid residues (DDES*) and TerL N-terminus (MMTKVK) is underlined; mutations introduced are in red.

Φ80α-vir

AGAGGAGAACCTCAAGAGGCTTACAGTAAGAAATATGACCATTTAAACGATGAAGT
GGAAAAAGAGGTTACTTACACAATCACACCAACTTTTGAAGAGCGTCAGAGATCTA
TTGACCACATACTAAAAGTTCATGGTGCGTATATCGACAAAAAAGAAATTACTCAGA
AGAATATTGAGATTAATATTGGTGAGTACGATGACGAAAGTTAAATTAAACTTTAAC
AAACCATCTAATGTTTTCAACAGAAACATATTCGAAATACTAACCAATTACGATAACT
TCACTGAAGTACATTACGGTGGAGGTTCGAGTGGTAAGTCTCACGGCGTTATACAA
AAAGTTGTACTTAAAGCATTGCAAGACTGGAAATATCCTAGGCGTATACTATGGCTT
AGA

Φ80α-vir(cabRNA[122])

AGGGGGGAGCCCCAAGAAGCCTATAGCAAAAAGTACGATCACTTGAATGACGAGG
TAGAGAAGGAAGTCACCTATACGATTACGCCGACCTTCGAGGAACGCCAAAGGTC
CATCGATCATATCCTGAAGGTCCACGGCGCATACATTGATAAGAAGGAGATCACCC
AAAAAAACATCGAAATCAACATCGGCGAATATGATGACGAAAGTTAAGTTGAATTTC
AATAAGCCGTCCAACGTCTTTAATAGGAATATATTTGAGATACTGACTAACTATGAC
AATTTTACCGAGGTGCACTATGGCGGGGGCTCTAGCGGCAAATCCCATGGTGTCA
TACAGAAGGTCGTGCTCAAGGCGTTACAGGATTGGAAGTACCCCAGACGCATACT
GTGGCTCAGG

Predicted RNA structure:

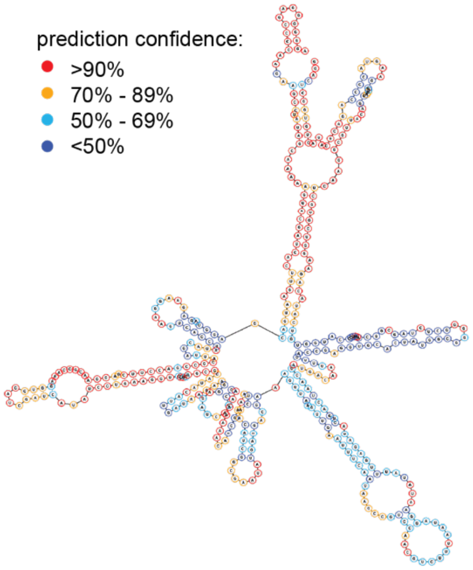

Φ80α-vir(cabRNA[100])

AGGGGGGAGCCCCAAGAAGCCTATAGCAAAAAGTACGATCACTTGAATGACGAGG
TAGAGAAGGAAGTCACCTATACGATTACGCCGACCTTCGAGGAACGCCAAAGGTC
CATCGATCATATCCTGAAGGTCCATGGTGCGTATATCGACAAAAAAGAAATTACTC

AGAAGAATATTGAGATTAATATTGGTGAGTACGATGACGAAAGTTAAGTTGAATTTC
AATAAGCCGTCCAACGTCTTTAATAGGAATATATTTGAGATACTGACTAACTATGAC
AATTTTACCGAGGTGCACTATGGCGGGGGCTCTAGCGGCAAATCCCATGGTGTCA
TACAGAAGGTCGTGCTCAAGGCGTTACAGGATTGGAAGTACCCCAGACGCATACT
GTGGCTCAGG

Predicted RNA structure:

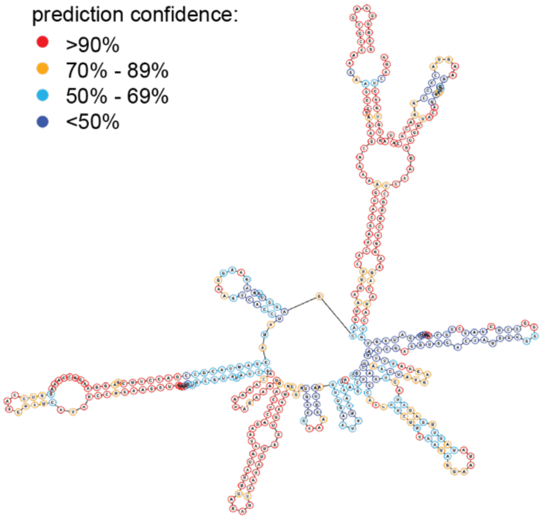

Φ80α-vir(cabRNA^{57})

AGAGGAGAACCTCAAGAGGCTTACAGTAAGAAATATGACCATTTAAACGATGAAGT
GGAAAAAGAGGTTACTTACACAATCACACCAACTTTTGAAGAGCGTCAGAGATCTA
TTGACCACATACTAAAAGTTCATGGTGCGTATATCGACAAAAAAGAAATTACTCAGA
AGAATATTGAGATTAATATTGGTGAGTACGATGACGAAAGTTAAGTTGAATTTCAAT
AAGCCGTCCAACGTCTTTAATAGGAATATATTTGAGATACTGACTAACTATGACAAT
TTTACCGAGGTGCACTATGGCGGGGGCTCTAGCGGCAAATCCCATGGTGTCATAC
AGAAGGTCGTGCTCAAGGCGTTACAGGATTGGAAGTACCCCAGACGCATACTGTG
GCTCAGG

Predicted RNA structure:

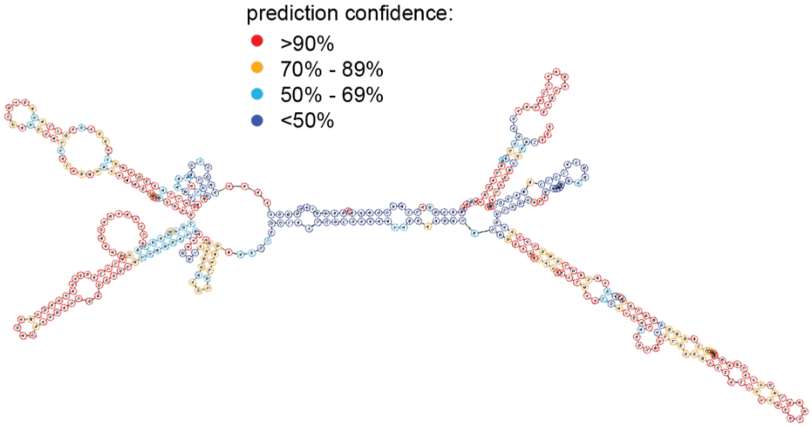

Supplement: Supplementary file 6 — Sequences and structures of the different RNAs described in this study. [file 41586_2023_6743_MOESM6_ESM.pdf]
